# Manganese(I)‐Catalyzed β‐Methylation of Alcohols Using Methanol as C_1_ Source

**DOI:** 10.1002/anie.201909035

**Published:** 2019-11-28

**Authors:** Akash Kaithal, Pit van Bonn, Markus Hölscher, Walter Leitner

**Affiliations:** ^1^ Institut für Technische und Makromolekulare Chemie RWTH Aachen University Worringer Weg 2 52074 Aachen Germany; ^2^ Max-Planck-Institut für chemische Energiekonversion Stiftstraße 34–36 45470 Mülheim a.d. Ruhr Germany

**Keywords:** alcohols, hydrogen borrowing, manganese catalysis, methanol, methylation

## Abstract

Highly selective β‐methylation of alcohols was achieved using an earth‐abundant first row transition metal in the air stable molecular manganese complex [Mn(CO)_2_Br[HN(C_2_H_4_P^*i*^Pr_2_)_2_]] **1** ([HN(C_2_H_4_P^*i*^Pr_2_)_2_]=MACHO‐^*i*^Pr). The reaction requires only low loadings of **1** (0.5 mol %), methanolate as base and MeOH as methylation reagent as well as solvent. Various alcohols were β‐methylated with very good selectivity (>99 %) and excellent yield (up to 94 %). Biomass derived aliphatic alcohols and diols were also selectively methylated on the β‐position, opening a pathway to “biohybrid” molecules constructed entirely from non‐fossil carbon. Mechanistic studies indicate that the reaction proceeds through a borrowing hydrogen pathway involving metal–ligand cooperation at the Mn‐pincer complex. This transformation provides a convenient, economical, and environmentally benign pathway for the selective C−C bond formation with potential applications for the preparation of advanced biofuels, fine chemicals, and biologically active molecules

## Graphical abstract

**Why not manganese?** A molecular catalyst based on manganese as the central metal allows the use of methanol as a benign reagent to install methyl groups in β‐position to alcohol functionalities, opening novel synthetic pathways to methyl‐branched products in line with green chemistry principles.

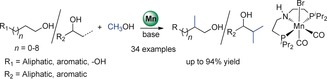

The formation of carbon–carbon bonds using commercially available building blocks is an important transformation for the toolbox of organic synthesis.^1^ In particular, the introduction of methyl groups in aliphatic carbon chains could open useful pathways for late stage generation of chain branches in functionalized molecules. As a structurally very important chemical motif, many pharmaceuticals and biologically active molecules contain at least one methyl group.^2^ Methyl branches are also favorable structural units in tailor‐made fuel components with advanced combustion properties.^3^ Conventionally, methylation reactions are performed using reagents such as Grignard reagents, methyl iodide or methyl sulfate, and diazomethane, which in general are highly flammable, toxic, and explosive (Scheme 1).^4^ Methanol provides a highly attractive alternative in the framework of the green chemistry principles, in particular when produced from biomass or from CO_2_ and H_2_.^5^ The methylation of biogenic substrates with CO_2_‐based methanol opens pathways to “biohybrid” molecules^6^ bridging between the bioeconomy and power‐to‐X concepts.

**Scheme 1:**
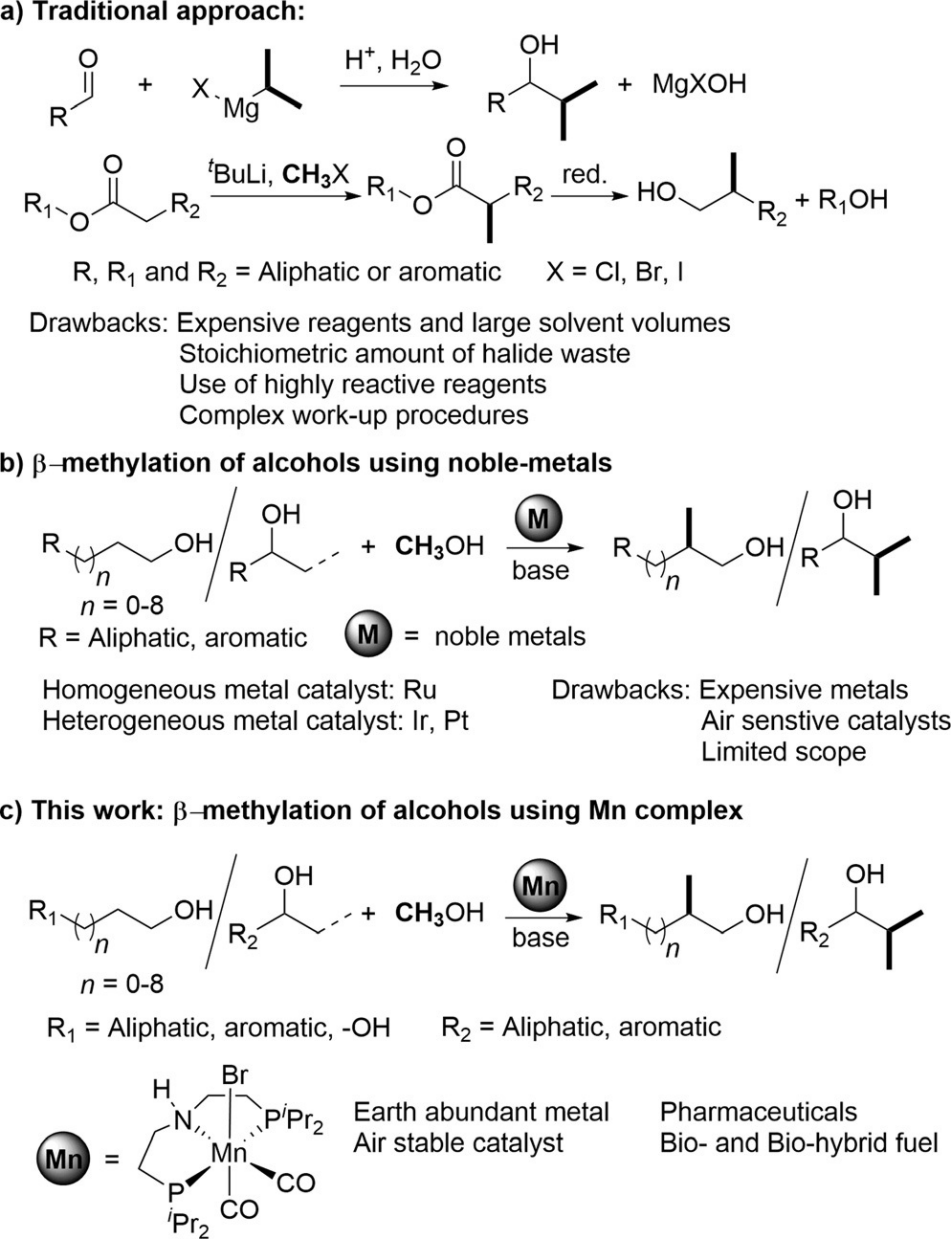
Traditional and metal‐catalyzed approach for the preparation of β‐methylated alcohols.

Recently, the selective β‐methylation of primary and secondary alcohols with methanol received increasing attention, but reports concerning this potentially useful reaction are still rare. Beller and co‐workers reported a homogeneous catalytic system for the selective β‐methylation of 2‐aryl ethanols using a mixture of two Ru‐complex catalyst.^7^ Heterogeneous catalyst reports based on Ir and Pt metals also showed promising activity for this transformation.^8^ We demonstrated that the reaction can be achieved by a single Ru^II^ pincer complex bearing the MACHO ligand.9a Mechanistic studies suggest that the Ru^II^ catalyst affects the dehydrogenation of alcohol and MeOH to the corresponding aldehyde/ketone and formaldehyde. A base mediates the aldol condensation between the carbonyl compounds. Subsequently the catalyst re‐hydrogenates the carbonyl bond and C−C coupled bond to provide the final β‐methylated product and complete the catalytic cycle.9b The metal–ligand cooperativity is of vital importance for the dehydrogenation/rehydrogenation steps in the complex reaction network.

Increasing efforts are currently devoted to the development of synthetic protocols where precious platinum group metals are replaced by earth abundant and cheap 3d‐metals in homogeneously catalyzed organic transformations.^10^ Lately, there is an increasing evidence suggesting that Ru^II^ centers can be replaced by more economical and environmental benign Mn^I^.10a, 11 A number of reactions such as hydrogenation,^12^ transfer‐hydrogenation,^13^ hydro‐elementation,5e, 14 and borrowing hydrogen reactions^15^ were reported using Mn^I^ complexes. In particular, selective alkylation reactions of secondary alcohols using primary alcohols as an alkylating reagent were reported with manganese and other 3d metal catalysts.15b, 16 The selective α‐methylation of ketones was also shown using Mn^I^ pincer complexes by Sortais and Rueping et al.^17^ Intrigued by these studies, we assumed that the Ru^II^/Mn^I^ substitution is particularly favorable due to the diagonal relationship between the two ions in the periodic table. Thus, we decided to explore whether this approach might allow the development of Mn^I^ catalysts for the selective β‐methylation of alcohols. While this manuscript was under revision, we became aware of an independent parallel study by the group of Morrill. They reported the selective β‐methylation of substituted aryl alcohols using an iron complex.^18^ The complex showed good reactivity towards the selective β‐methylation of substituted 2‐arylethanols. However, the reaction with secondary alcohols or aliphatic alcohols revealed very low reactivity towards the desired methylated product.

At the outset, 2‐phenyl ethanol (**6 a**) was selected as a benchmark substrate to validate the rational of catalyst selection and for optimization of the reaction conditions (Table 1). As predicted, the Mn^I^‐MACHO complex [Mn(CO)_2_Br[HN(C_2_H_4_P^*i*^Pr_2_)_2_]] (**1**) showed by far the highest activity and selectivity from a range of pre‐catalysts **1**–**5**. Variation of reaction parameters such as catalyst loading, temperature, and amount of base lead to the definition of a standard set of conditions using 0.5 mol % of complex **1** with NaOMe (2 equiv with respect to **6 a**) at 150 °C for 24 h. Conversion of **6 a** reached 97 % under these conditions forming selectively the β‐methylated product **7 a** with 92 % yield, as evidenced by NMR analysis of the reaction mixture. Product **7 a** was isolated in 85 % yield after column chromatography.

**Table 1:** Mn^I^ catalyzed β‐methylation of **6 a** with methanol: Influence of catalyst precursors and reaction conditions.^[a,b]^ 
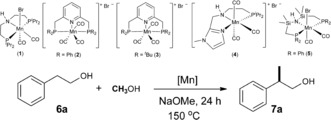

#	Catalyst	Conv. [%]	Yield [%]
1	1 (0.5 mol %)	97	92
2	2 (0.5 mol %)	42	21
3	3 (0.5 mol %)	51	24
4	4 (0.5 mol %)	23	6
5	5 (0.5 mol %)	10	0
6	1 (0.2 mol %)	66	52
7^[c]^	1 (0.5 mol %)	76	70
8^[d]^	1 (0.5 mol %)	77	70

[a] **6 a** (1 mmol), MeOH (1 mL as a reagent and solvent), Mn precatalyst (0.5 mol %), and NaOMe (2 mmol) at 150 °C for 24 h. [b] Conversion and yield were measured by ^1^HNMR and mesitylene was used as an internal standard. [c] Reaction was carried out at 125 °C. [d] 1 mmol of NaOMe was used.

Using the standard conditions, various 2‐aryl ethanols were investigated as substrates for selective β‐methylation (Table 2). Similar high conversions and good yields as for **6 a** were observed for substrates with electron‐donating substituents such as 2‐(*p*‐tolyl)ethanol (**6 b**) and 2‐(4‐methoxyphenyl)ethanol (**6 d**). Somewhat lower yield was obtained for **6 e** bearing the methoxy substituent in *meta*‐position, while 2‐(4‐chlorophenyl)ethanol (**6 g**) and 2‐(4‐fluorophenyl)ethanol (**6 h**) were also converted with 81 % and 87 % yield, respectively. Heterocyclic alcohol such as thiophene substituted ethanol (**6 i**) was well tolerated and afforded 77 % yield to the corresponding methylated product **7 i**. The reaction with pyridine substituted 2‐(pyridin‐2‐yl)ethan‐1‐ol gave a mixture of products (not shown). However, indole substituted ethanol (**6 j**) yielded 54 % of the desired β‐methylated product. Notably, pharmaceutically important molecules such as ibuprofen alcohol and naproxen alcohol could be prepared also with very good yields (**7 c** and **7 f**, respectively).

**Table 2:** Mn^I^ catalyzed β‐methylation of 2‐arylethanols with methanol.^[a,b]^ 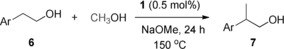

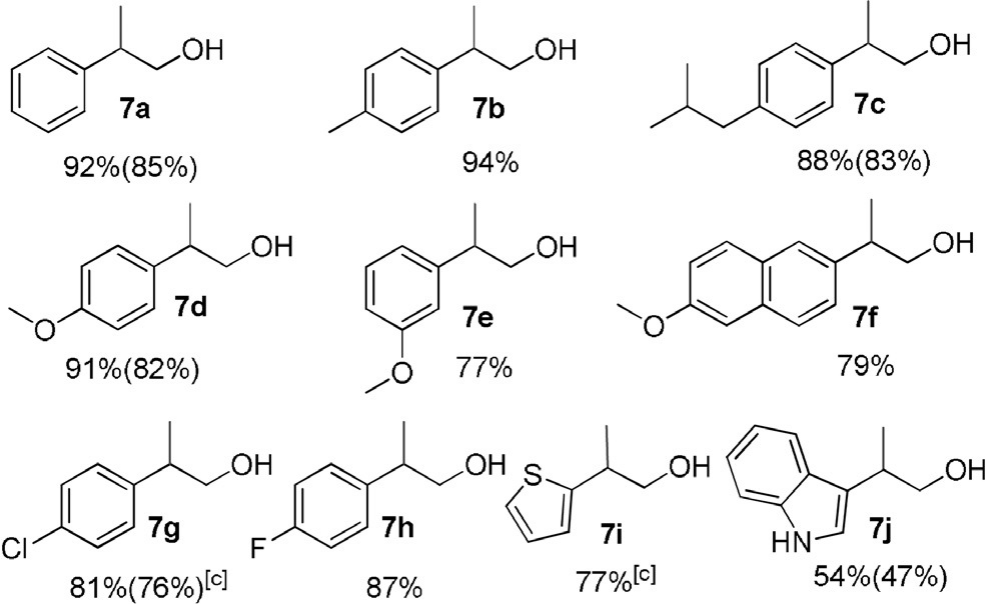

[a] **6** (1 mmol), MeOH (1 mL as a reagent and solvent), Mn precatalyst **1** (0.5 mol %), and NaOMe (2 mmol) at 150 °C for 24 h. [b] Yields were determined by ^1^HNMR analysis using mesitylene as an internal standard. Yields in parenthesis correspond to the isolated product after performing column chromatography. [c] Reaction time: 14 h.

Next, we focused on the selective β‐methylation of secondary alcohols (Table 3). 1‐Phenyl ethanol (**8 a**) was chosen as parent substrate for this class of compounds. Upon increasing the amount of a base to 4 equivalents and using 0.5 mol % of **1** at 150 °C for 36 h, the selectively di‐methylated product **9 a** was observed with a 77 % yield. Reaction of 1‐(naphthalen‐2‐yl)ethan‐1‐ol (**8 b**) and 1‐(*p*‐tolyl)ethanol (**8 c**) with methanol under these conditions led to selective formation to the di‐methylated product with a yield of 82 % and 73 %, respectively. However, substrate **8 d** resulted only in 29 % di‐methylated product, probably reflecting an unfavorable steric influence of the *ortho*‐methyl substituent. The methoxy‐substituent in *para*‐position of 1‐phenyl ethanol (**8 e**) yielded 57 % of the di‐methylated product **9 e**. The electron withdrawing fluoro substituent in *para*‐substituted (**8 f**) resulted in 44 % yield of the di‐methylated product under standard reaction conditions. Prolonging the reaction time to 42 h increased the yield of the desired di‐methylated product **9 f** to 61 %. Furan was tolerated as substituent affording the desired product **9 g** in 63 % yield. 1‐Cyclohexylethan‐1‐ol (**8 h**) exhibited lower reactivity with 31 % yield of di‐methylated product **9 h**. Complex **1** showed excellent activity for the selective β‐methylation of 5‐, 6‐, and 7‐ membered ring alcohols (**8 i**–**8 k**), with preferential formation of the *trans* isomers.

**Table 3:** Mn^I^ catalyzed β‐methylation of secondary alcohols with methanol.^[a,b]^ 
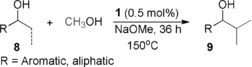

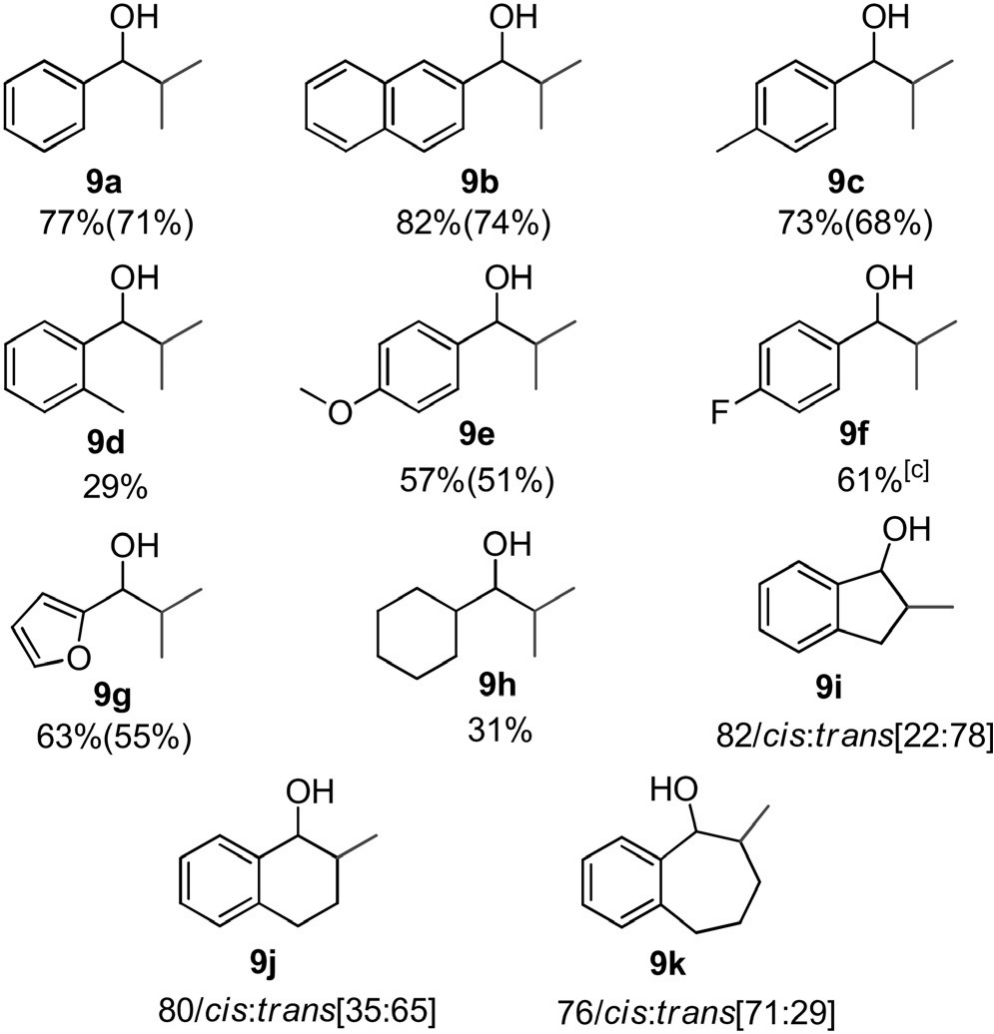

[a] **8** (1 mmol), MeOH (1 mL as a reagent and solvent), Mn precatalyst **1** (0.5 mol %), and NaOMe (4 mmol) at 150 °C for 36 h. [b] Yields were determined by ^1^HNMR using mesitylene as an internal standard. Yields in parenthesis correspond to the isolated product after performing column chromatography. [c] Reaction time was increased to 42 h.

Encouraged by these results, we further investigated the selective β‐methylation of aliphatic alcohols (Table 4). It turned out that 2 equivalents of methanolate with respect to the substrate worked best for this transformation. Under these conditions, 3‐phenyl‐ethanol (**10 a**) was selectively converted with 71 % yield to the desired β‐methylated product. The chloro‐substituent in *para*‐position of 3‐phenyl‐ethanol (**10 b**) was well tolerated resulting in 64 % yield. Upon increasing the length of the carbon chain between the aromatic ring and the reactive position, yields dropped slightly, but around 60 % of the desired product was consistently observed (**10 c**, **10 d**). Gratifyingly, these conditions could be applied also to biomass‐derived aliphatic alcohols and diols. Reacting ethanol in presence of complex **1** in methanol/methanolate, selective formation of isobutanol (**11 e**) was obtained with 46 % yield. These conditions were also implemented to other aliphatic alcohols such as 1‐butanol (**10 f**) and 1‐pentanol (**10 g**) which also revealed good to moderate yield of the selective β‐methylated product. Interestingly, when the length of the carbon chain was increased, yields also increased up to 75 % for long chain alcohols as derived from fatty acids. Diols were also methylated at both possible positions with nearly equal efficiency, as shown for 1,6‐hexanediol (**10 l**) and 1,10‐decanediol (**10 m**).

**Table 4:** Mn^I^ catalyzed β‐methylation of aliphatic alcohols and diols with methanol.^[a,b]^ 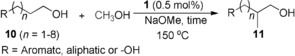

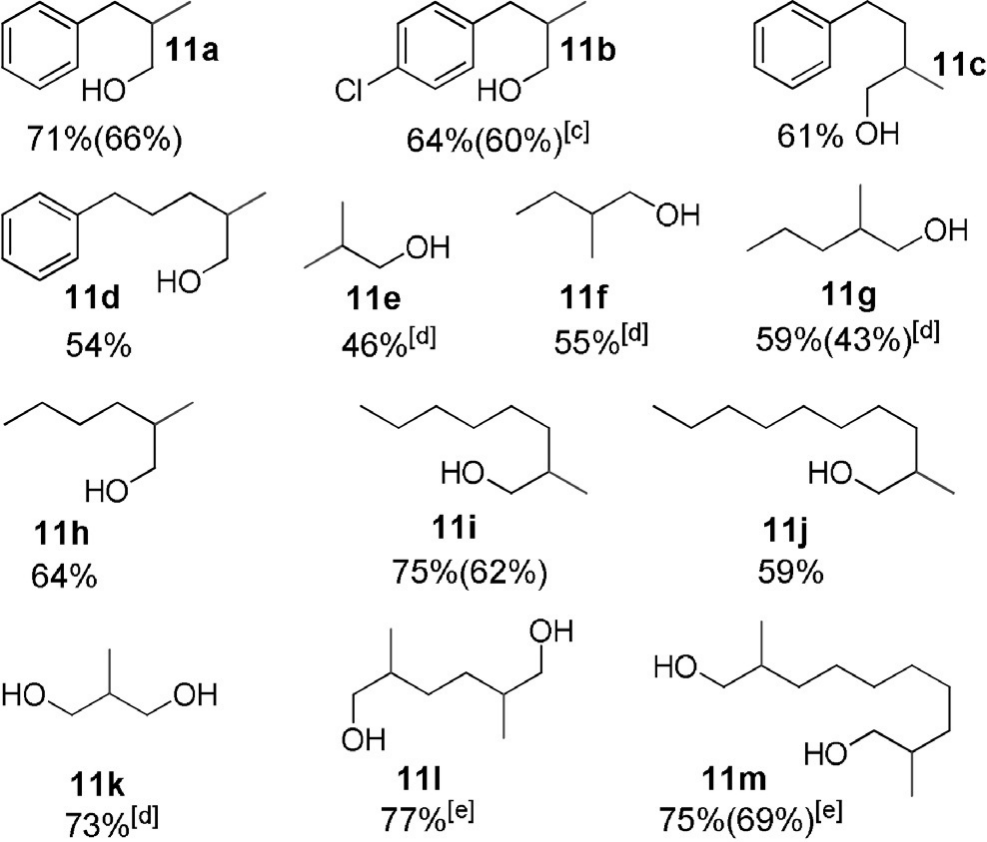

[a] **10** (1 mmol), MeOH (1 mL as a reagent and solvent), Mn precatalyst **1** (0.5 mol %), and NaOMe (2 mmol) at 150 °C for 24 h. [b] Yields were determined by ^1^H NMR using mesitylene as an internal standard. Yields in parenthesis correspond to the isolated product after performing column chromatography. [c] Reaction time: 14 h. [d] Reaction time: 36 h. [e] 4 mmol of NaOMe was used and reaction time was increased to 48 h.

When 1,3‐propanediol (**10 k**), was selectively β‐methylated using Mn‐complex **1**, 2‐methyl‐1,3‐propanediol (MPO, **11 k**) was obtained in 73 % yield under standard conditions. MPO is an important large volume product with chemical and consumer use, which is currently produced exclusively from fossil feedstocks. The new pathway demonstrated herein makes this product accessible as “biohybrid” molecule entirely from renewable carbon sources.

In order to validate the concept of a mechanistically driven substitution of Ru^II^ catalysts by Mn^I^ complexes, a series of control experiments was carried out (Scheme 2). Using ^13^CH_3_OH together with sodium *tert*‐butoxide as the base, in toluene as the solvent, unequivocally showed that methanol was the source of the methyl group. Using CD_3_OD led to deuterium incorporation in β‐ as well as α‐position, supporting a borrowing hydrogen mechanism. Reaction of 2‐phenyl ethanol (**6 a**) with paraformaldehyde in presence of hydrogen gave 17 % yield of the β‐methylated product **7 a**. Considering the limited solubility of paraformaldehyde in toluene, this result supports the assumption that formaldehyde is intermediate. The transformation of acetophenone (**14**) with methanol also led to the dimethylated product **9 a** with a yield of 7 %. A variety of side products were observed in the ^1^H NMR spectrum resulting from aldol‐type side reactions at the high concentration of **14**. Stoichiometric reactions of complex **1** with base, followed by addition of alcohol **6 a** or methanol confirmed the formation of alcoholate complexes **16** and **17** via the unsaturated Mn‐amide complex **15** (Scheme 3).12b, 19 In case of methanol, formation of the hydride complex **18** together with formaldehyde and H_2_ clearly demonstrated the dehydrogenation activity of the active species **15**.

**Scheme 2:**
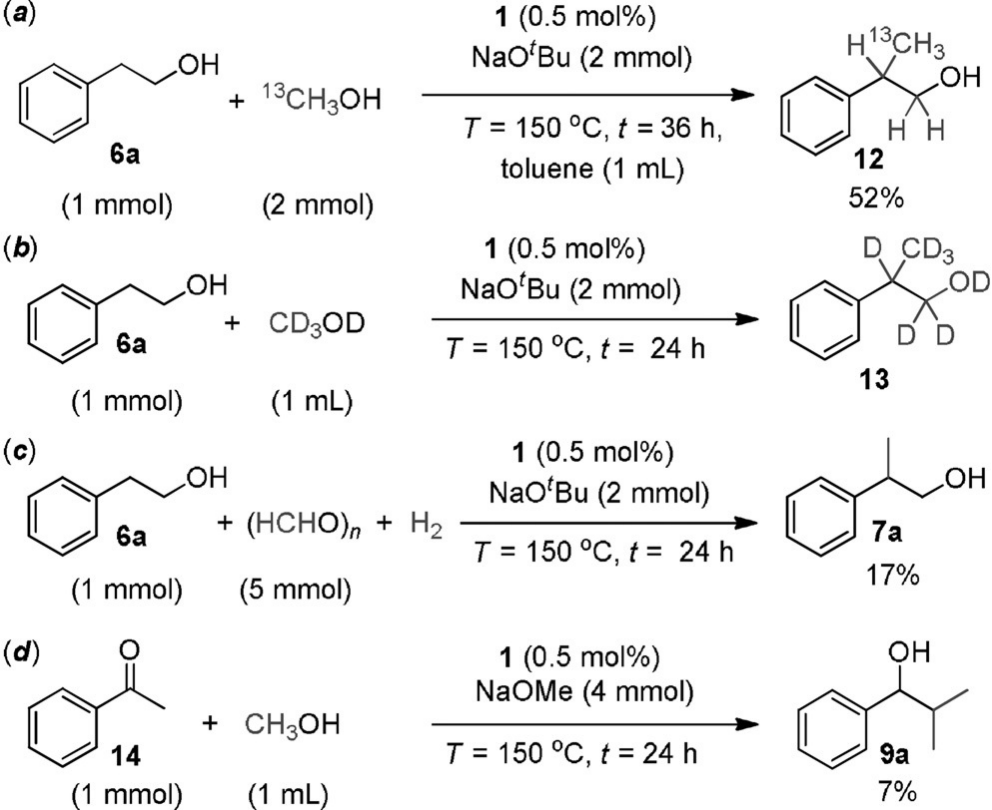
Labeling experiments with ^13^C and ^2^H labeled methanol and reactivity of plausible intermediates.

**Scheme 3:**
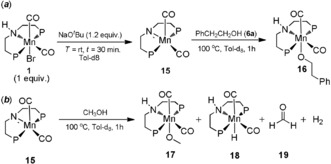
Spectroscopically identified Mn^I^ intermediates obtained by stoichiometric reaction with Mn complex **1**.

Analysis of the composition of reaction mixtures after different reaction times for the selective β‐methylation of 2‐phenyl‐ethanol **6 a** using complex **1** under standard conditions showed a very distinct development of conversion and yield over time (Figure 1).

**Figure 1:**
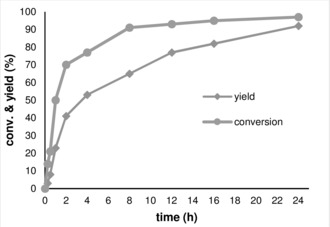
Conversion/time profile for the β‐methylation of 1‐phenyletahnol (**6 a**) using Mn^I^‐MACHO complex **1** in methanol as solvent as derived from ^1^HNMR analysis of reaction mixtures at given time intervals. **6 a** (1 mmol), MeOH (1 mL), Mn precatalyst **1** (0.5 mol %), and NaOMe (2 mmol), 150 °C.

Within the first 2 h of the reaction, conversion reaches close to 70 %, while the yield of the methylated product is only 41 %. In general, there is a significant and growing gap between conversion and yield during the first 10 h of reaction until the reaction reaches close to full consumption of the starting material **6 a**. Then, the selectivity starts to catch up until conversion and yield are both close to 90 % after 24 h. This can be rationalized assuming that 2‐phenyl ethanol (**6 a**) and methanol are both converted relatively fast to phenylacetaldehyde (**6 a′**) and formaldehyde with the liberation of H_2_. With the base‐catalyzed aldol condensation being the slowest step in the catalytic network, the formation of the final product is delayed relative to substrate conversion.9b Consequently, the use of (over‐)stoichiometric amounts of base is beneficial to achieve practical reaction rates. While only small amounts of base would be necessary to activate the Mn‐catalyst, its role to accelerate the aldol condensation is critical. Otherwise, the build‐up of the carbonyl intermediates in solution and hydrogen in the gas phase shifts the first equilibria back to the substrates. This would block the pathway to the desired products completely as encountered for example in other studies on alkylation reactions based on the borrowing hydrogen concept.^7^ The formation of alkene‐type aldol coupling intermediates was observed in ^1^H NMR while performing the time‐conversion profile.

Based on these observations and in accord with literature knowledge on Ru^II^‐based borrowing hydrogen catalysis^7^, ^20^ and Mn^I^ H‐transfer reactions,12b, 12d, 13a, 19, 21 a plausible mechanism for the Mn^I^‐MACHO (**1**) catalyzed β‐methylation can be proposed as shown in Figure 2. Ligand‐assisted de‐/re‐hydrogenation is the prominent feature for the metal‐catalyzed steps. Further experimental and computational efforts are required to elucidate these catalytic networks in detail.

**Figure 2:**
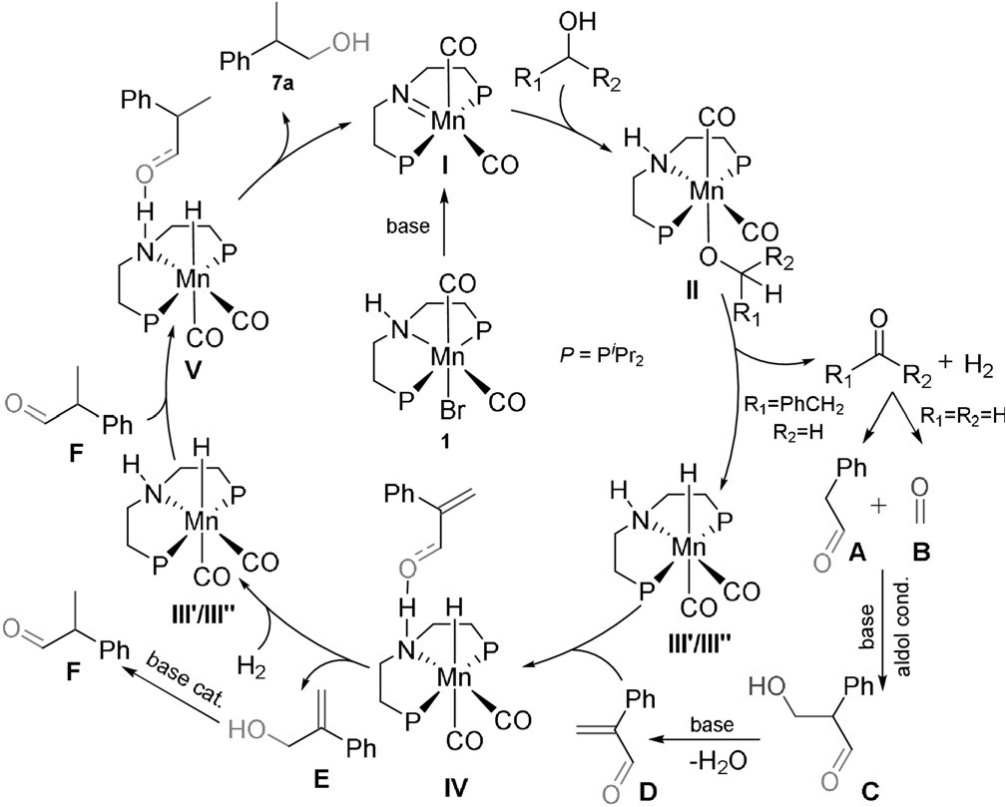
Plausible reaction mechanism for Mn^I^‐catalyzed β‐methylation of alcohols using complex **1** in methanol as solvent.

In conclusion, the β‐methylation of alcohols can be achieved using the earth abundant first row transition metal manganese in form of its pincer complex **1** as pre‐catalyst and methanol as solvent and C_1_ source. The activity of **1** for β‐methylation is applicable to a large variety of alcohols such as secondary, primary, and cyclic alcohols with very good yield and excellent selectivity using a low loading of catalyst. Biomass‐derived alcohols and diols are shown, for the first time, to be selectively methylated to the corresponding product, including the preparation of MPO from 1,3‐propane diol and methanol. The yields of methylated products match and even surpass in many cases those achieved with noble metal catalysts. Mechanistic studies confirmed that the reaction follows a borrowing hydrogen pathway, fostering the concept of exploiting the diagonal relationship between Ru^II^ and Mn^I^ for the design of highly efficient catalysts based on manganese as benign metal component.

## Conflict of interest

The authors declare no conflict of interest.

## Supporting information

As a service to our authors and readers, this journal provides supporting information supplied by the authors. Such materials are peer reviewed and may be re‐organized for online delivery, but are not copy‐edited or typeset. Technical support issues arising from supporting information (other than missing files) should be addressed to the authors.

Supplementary
