# Supplementary material for: Manganese(I)‐Catalyzed β‐Methylation of Alcohols Using Methanol as C1 Source
Source: Angew Chem Int Ed Engl. 2019 Nov 28;59(1):215–20. doi: 10.1002/anie.201909035 (PMC6973237; doi:10.1002/anie.201909035)
Supplement: Supplementary file 1 — Supplementary [file ANIE-59-215-s001.pdf]

Angewandte Chemie
Eine Zeitschrift der Gesellschaft Deutscher Chemiker

Supporting Information

Manganese(I)-Catalyzed β-Methylation of Alcohols using Methanol as C_1 Source

*Akash Kaithal, Pit van Bonn, Markus Hölscher, and Walter Leitner**

anie_201909035_sm_miscellaneous_information.pdf

Contents

1. General Experimental 2
2. Synthesis of Manganese pincer complexes 3
3. General procedure for screening of Mn-catalysts 4
4. General procedure for the catalytic selective β-methylation of 2-aryl ethanols 5
5. General procedure for the catalytic selective β-methylation of secondary alcohols 9
6. General procedure for catalytic selective β-methylation of aliphatic alcohols 13
7. General procedure for the catalytic selective β-methylation of diols 17
8. ^{1}H NMR spectra of reaction mixtures for β-methylated alcohols 19
9. NMR spectra of isolated products 38
10. Conversion/time profile from experiments for the β-methylation of 6a with MeOH at different time intervals 53
11. Labeling experiments and observation of potential intermediates 55
    - 11.1. Procedure for β-methylation of 6a with $^{13}CH_3OH$ 55
    - 11.2. Procedure for selective β-methylation of 6a with CD_3OD 56
    - 11.3. Procedure for β-methylation of 6a with paraformaldehyde and H_2 57
    - 11.4. Procedure for β-methylation of Acetophenone (14) with methanol 58
    - 11.5. Preparation of complex 15 59
    - 11.6. Reaction of Mn-complex (1) with 2-phenyl ethanol (6a) 60
    - 11.7. Reaction of Mn-complex (15) with methanol 62

References 64

1. General Experimental

All catalytic and stoichiometric reactions were performed under argon atmosphere using a combination of Schlenk and glove box techniques. Chemicals were purchased from Sigma-Aldrich, Alfa-Aesar, TCI chemicals and used without further purification. Dry solvents were prepared according to standard procedures. Glasswares were dried under vacuum at high temperatures, evacuated, and refilled with argon at least three times. ^{1}H, ^{13}C, and ^{31}P NMR spectra were recorded with spectrometers Bruker AV300 or AV400 at room temperature. The solvent signals were used as references and the chemical shifts converted to the TMS scale ($CDCl_3$: δ_H = 7.26 ppm, δ_C = 77.3 ppm; C_6D_6: δ_H = 7.16 ppm, δ_C = 127.6 ppm; THF-d_8: δ_H = 1.72 ppm, δ_C = 24.2 ppm; Toluene-d_8: δ_H = 2.1 ppm, δ_C = 21.4 ppm). Chemical shifts for ^{31}P are reference against H_3PO_4 as external standard. Multiplicity is abbreviated as: s, singlet; d, doublet; t, triplet; q, quartet; sext, sextet; m, multiplet; br, broad.

Catalytic reactions involving high pressure gases were carried out in home built stainless steel reactors equipped with pressure transducer and external electrical heating.

Safety advice: High-pressure experiments represent a significant risk and must be conducted with appropriate safety procedures and in conjunction with the use of suitable equipment.

2. Synthesis of Manganese pincer complexes

Manganese pincer complexes **1**, **2**, **3** and **4** were prepared according to the reported literature: A. Kaithal, M. Hölscher, W. Leitner. Catalytic Hydrogenation of Cyclic Carbonates using Manganese Complexes. *Angew. Chem. Int. Ed.* **2018**, *57*(41): 13449-13453.[1]

Manganese pincer complex **5** was prepared according to the reported literature: A. Kaithal, S. Sen, C. Erken, T. Weyhermüller, M. Hölscher, C. Werlé, W. Leitner. Manganese-catalyzed hydroboration of carbon dioxide and other challenging carbonyl groups. *Nat. Commun.,* DOI: 10.1038/s41467-018-06831-9.[2]

3. General procedure for screening of Mn-catalysts

Mn-precursor and NaOMe (108.04 mg, 2 mmol) were measured into a glass inlet equipped with a stirring bar inside a glovebox. The glass inlet was closed with a septum and transferred into the bottom part of the 10 mL steel autoclave, where it was opened under a stream of argon. After sealing, the autoclave was purged with argon three times. 2-phenyl ethanol **6a** (122.2 mg, 1 mmol) and methanol (1 mL) were added at room temperature through a valve under argon. The autoclave was sealed and heated to at certain temperature. After 24 h, the autoclave was cooled to room temperature and slowly vented while stirring continued. Mesitylene was added as an internal standard to the reaction mixture that was then passed through a short path of acidic alumina before the composition was analyzed by NMR spectroscopy.

Table S1: Mn (I) catalyzed *β*-methylation of 6a with methanol: Influence of catalyst precursors and reaction conditions.[a, b]

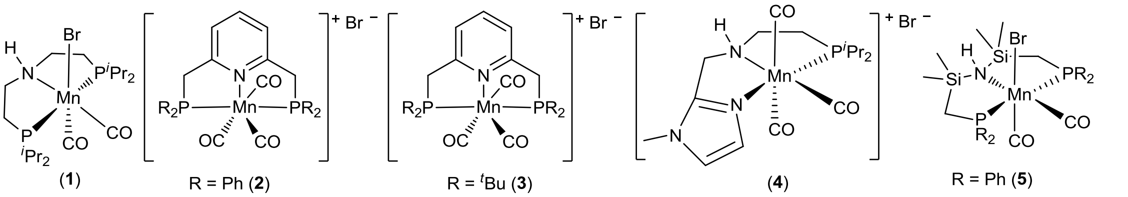

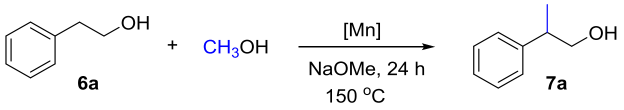

#	Catalyst	Conv. (%)	Yield (%)
1	**1** (0.5 mol%)	97	92
2	**2** (0.5 mol%)	42	21
3	**3** (0.5 mol%)	51	24
4	**4** (0.5 mol%)	23	6
5	**5** (0.5 mol%)	10	0
6	**1** (0.2 mol%)	66	52
7[c]	**1**(0.5 mol%)	76	70
8[d]	**1**(0.5 mol%)	77	70

[a] **6a** (1 mmol), MeOH (1 mL as a reagent and solvent), Mn precatalyst (0.5 mol%), and NaOMe (2 mmol) at 150 °C for 24 h. [b] Conversion and yield were measured by ^{1}H NMR and mesitylene was used as an internal standard. [c] Reaction was carried out at 125 °C. [d] 1 mmol of NaOMe was used.

4. General procedure for the catalytic selective β-methylation of 2-aryl ethanols

Mn-MACHO **1** (2.48 mg, 0.5 mol%) and NaOMe (108.04 mg, 2 mmol) were measured into a glass inlet equipped with a stirring bar inside a glovebox. The glass inlet was closed with a septum and transferred into the bottom part of the 10 mL steel autoclave, where it was opened under a stream of argon. After sealing, the autoclave was purged with argon three times. 2-aryl ethanol (1 mmol) and methanol (1 mL) were added at room temperature through a valve under argon. The autoclave was sealed and heated to 150 ^{o}C temperature for 24 h. After completion of the reaction, the autoclave was cooled to room temperature and slowly vented while stirring continued. Mesitylene was added as an internal standard to the reaction mixture that was then passed through a short path of acidic alumina before the composition was analyzed by NMR spectroscopy. The isolation of pure product was carried out using column chromatography over silica gel (100-200 mesh) using ethyl acetate/petroleum ether (12 : 88) mixture as eluent.

2-phenylpropan-1-ol (7a): Prepared by following the general experimental procedure with:

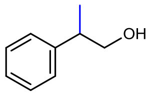

1 (2.48 mg, 0.5 mol%), 2-phenylethan-1-ol **6a** (122.2 mg, 1 mmol), NaOMe (108.04 mg, 2 mmol), MeOH (1 mL). Yield was determined by ^{1}H NMR spectrum using mesitylene (120 mg, 1 mmol) as an internal standard ($\delta_{\text{Mesitylene(standard)}}$ = 6.72 (s, 3H), δ_{product} = 3.63 (d, 2H)).

^{1}H NMR (400 MHz, $CDCl_3$, 298 K) δ = 7.24-7.28 (m, 2H, ArC*H*), 7.14-7.18 (m, 3H, ArC*H*), 3.63 (d, 2H, *J* = 6.78 Hz, CH_2), 2.83-2.90 (sext, 1H, *J* = 6.94 Hz, C*H*), 1.40 (br. s, 1H, O*H*), 1.21 (d, 3H, *J* = 6.97 Hz, CH_3). **^{13}C{^{1}H}-NMR** (101 MHz, $CDCl_3$, 298 K) δ = 143.77 (quat-*C*), 128.77 (Ar*C*H), 127.61 (Ar*C*H), 126.81 (Ar*C*H), 68.85 (CH_2), 42.57 (*C*H), 17.71 (CH_3). Isolated yield: 85%. The obtained analytical data is consistent with those previously reported in the literature.[3]

2-(*p*-tolyl)propan-1-ol (7b): Prepared by following the general experimental procedure with: **1** (2.48 mg, 0.5 mol%), 2-(*p*-tolyl)ethan-1-ol **6b** (136.2 mg, 1 mmol), NaOMe (108.04 mg, 2 mmol), MeOH

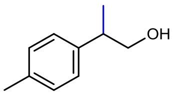

(1 mL). Yield was determined by ^{1}H NMR spectrum using mesitylene (120 mg, 1 mmol) as an internal standard ($\delta_{\text{Mesitylene(standard)}}$ = 6.82 (s, 3H), δ_{product} = 3.69 (d, 2H)).

^{1}H NMR (300 MHz, $CDCl_3$, 298 K) δ = 7.15 (s, 4H, ArC*H*), 3.69 (d, 2H, *J* = 9 Hz, CH_2), 2.90-2.97 (m, 1H, C*H*), 2.35 (s, 3H, CH_3), 1.27 (d, 3H, *J* = 9 Hz, CH_3) ppm. The obtained analytical data is consistent with those previously reported in the literature.[3]

2-(4-*iso*-butylphenyl)propan-1-ol. (7c): Prepared by following the general experimental procedure with:

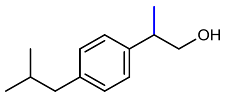

1 (2.48 mg, 0.5 mol%), 2-(4-isobutylphenyl)ethan-1-ol **6c** (178.3 mg, 1 mmol), NaOMe (108.04 mg, 2 mmol), MeOH (1 mL). Yield was determined by ^{1}H NMR spectrum using mesitylene (120 mg, 1 mmol) as an internal standard ($\delta_{Mesitylene(standard)}$ = 6.73 (s, 3H), $\delta_{product}$ = 3.60 (d, 2H)).

^{1}H NMR (400 MHz, $CDCl_3$, 298 K) δ = 7.10-7.16 (m, 4H, ArC*H*), 3.69 (d, 2H, *J* = 6.84 Hz, CH_2), 2.93 (sext, 1H, *J* = 6.94 Hz,.C*H*), 2.45 (d, 2H, *J* = 7.13 Hz, CH_2), 1.80-1.90 (m, 1H, C*H*), 1.43 (br. s, 1H, OH), 1.27 (d, 3H, *J* = 7.03 Hz, CH_3), 0.91 (d, 6H, *J* = 6.61 Hz, CH_3). **^{13}C{^{1}H}-NMR** (101 MHz, $CDCl_3$, 298 K) δ = 140.83 (quat-*C*), 140.20 (quat-*C*), 129.51 (Ar*C*H), 127.29 (Ar*C*H), 68.93 (*C*H_2), 45.17 (*C*H_2), 42.17 (*C*H), 30.35 (*C*H), 22.55 (*C*H_3), 17.75 (*C*H_3). Isolated yield: 83%. The obtained analytical data is consistent with those previously reported in the literature.[4]

2-(4-methoxyphenyl)propan-1-ol (7d): Prepared by following the general experimental procedure with:

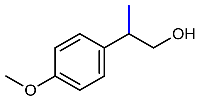

1 (2.48 mg, 0.5 mol%), 2-(4-methoxyphenyl)ethan-1-ol **6d** (152.2 mg, 1mmol), NaOMe (108.04 mg, 2 mmol), MeOH (1 mL). Yield was determined by ^{1}H NMR spectrum using mesitylene (120 mg, 1 mmol) as an internal standard ($\delta_{Mesitylene(standard)}$ = 2.31 (s, 9H), $\delta_{product}$ = 1.25 (d, 3H)).

^{1}H NMR (400 MHz, $CDCl_3$, 298 K) δ = 7.16 (d, 2H, *J* = 8.65 Hz, ArC*H*), 6.88 (d, 2H, *J* = 8.68 Hz, ArC*H*), 3.80 (s, 3H, OCH_3), 3.66 (dd, 2H, *J* = 6.86 Hz, 3.58 Hz, CH_2), 2.90 (sext, 1H, *J* = 6.90 Hz, C*H*), 1.87 (br. s, 1H, O*H*), 1.25 (d, 3H, *J* = 7 Hz, CH_3). **^{13}C{^{1}H}-NMR** (101 MHz, $CDCl_3$, 298 K) δ = 158.48 (quat-*C*), 135.70 (quat-*C*), 128.52 (Ar*C*H), 114.19 (Ar*C*H), 68.95 (*C*H_2), 55.41 (O*C*H_3), 41.71 (*C*H), 17.87 (*C*H_3). Isolated yield: 82%. The obtained analytical data is consistent with those previously reported in the literature.[3]

2-(3-methoxyphenyl)propan-1-ol (7e): Prepared by following the general experimental procedure with:

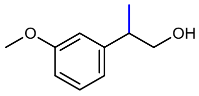

1 (2.48 mg, 0.5 mol%), 2-(3-methoxyphenyl)ethan-1-ol **6e** (152.2 mg, 1 mmol), NaOMe (108.04 mg, 2 mmol), MeOH (1 mL). Yield was determined by ^{1}H NMR spectrum using mesitylene (120 mg, 1 mmol) as an internal standard ($\delta_{Mesitylene(standard)}$ = 2.30 (s, 9H), $\delta_{product}$ = 1.29 (d, 3H)).

^{1}H NMR (300 MHz, $CDCl_3$, 298 K) δ = 7.24-7.30 (m, 1H, ArC*H*), 6.78-6.87 (m, 3H, ArC*H*), 3.82 (s, 3H, CH_3), 3.71 (d, 2H, *J* = 6 Hz, CH_2), 2.86-2.95 (m, 1H, C*H*),1.28 (d, 3H, *J* = 9 Hz, CH_3) ppm. The obtained analytical data is consistent with those previously reported in the literature.[4]

2-(6-methoxynaphthalen-2-yl)propan-1-ol (7f): Prepared by following the general experimental

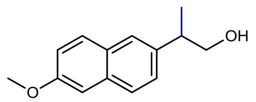

procedure with: **1** (2.48 mg, 0.5 mol%), 2-(6-methoxynaphthalen-2-yl)ethan-1-ol **6f** (202.3 mg, 1 mmol), NaOMe (108.04 mg, 1 mmol), MeOH (1 mL). Yield was determined by ^{1}H NMR spectrum using mesitylene (120 mg, 1 mmol) as an internal standard ($\delta_{Mesitylene(standard)}$ = 6.72 (s, 3H), $\delta_{product}$ = 1.26 (d, 3H)).

^{1}H NMR (300 MHz, $CDCl_3$, 298 K) δ = 7.60-7.64 (m, 2H, ArC*H*), 7.52 (s, 1H, ArC*H*), 7.24-7.26 (m, 1H, ArC*H*), 7.02-7.07 (m, 2H, ArC*H*), 3.82 (s, 3H, OCH_3), 3.68 (d, 2H, *J* = 6 Hz, CH_2), 2.96-3.03 (m, 1H, C*H*), 1.26 (d, 3H, *J* = 6 Hz, CH_3). The obtained analytical data is consistent with those previously reported in the literature.[5]

2-(4-chlorophenyl)propan-1-ol (7g): Prepared by following the general experimental procedure with:

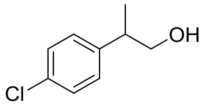

1 (2.48 mg, 0.5 mol%), 2-(4-chlorophenyl)ethan-1-ol **6g** (156.6 mg, 1 mmol), NaOMe (108.04 mg, 2 mmol), MeOH (1 mL). Reaction time: 14 h. Yield was determined by ^{1}H NMR spectrum using mesitylene (120 mg, 1 mmol) as an internal standard ($\delta_{Mesitylene(standard)}$ = 6.83 (s, 3H), $\delta_{product}$ = 3.68 (dd, 2H)).

^{1}H NMR (400 MHz, $CDCl_3$, 298 K) δ = 7.30 (d, 2H, *J* = 8.41 Hz, ArC*H*), 7.17 (d, 2H, *J* = 8.40 Hz, ArC*H*), 3.68 (dd, 2H, *J* = 6.79 Hz, 2.14 Hz, CH_2), 2.93 (sext, 1H, *J* = 6.95 Hz, C*H*), 1.55 (br. s, 1H, O*H*), 1.25 (d, 3H, *J* = 6.99 Hz, CH_3). **^{13}C{^{1}H}-NMR** (101 MHz, $CDCl_3$, 298 K) δ = 142.35 (quat-*C*), 132.44 (quat-*C*), 128.96 (Ar*C*H), 128.86 (Ar*C*H), 68.61 (CH_2), 41.98 (*C*H), 17.67 (CH_3). Isolated yield: 76%. The obtained analytical data is consistent with those previously reported in the literature.[6]

2-(4-fluorophenyl)propan-1-ol (7h): Prepared by following the general experimental procedure with:

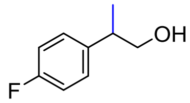

1 (2.48 mg, 0.5 mol%), 2-(4-fluorophenyl)ethan-1-ol **6h** (140.2 mg, 1 mmol), NaOMe (108.04 mg, 2 mmol), MeOH (1 mL). Yield was determined by ^{1}H NMR spectrum using mesitylene (124 mg, 1.03 mmol) as an internal standard ($\delta_{Mesitylene(standard)}$ = 6.83 (s, 3H), $\delta_{product}$ = 3.68 (d, 2H)).

^{1}H NMR (300 MHz, $CDCl_3$, 298 K) δ = 7.19-7.26 (m, 2H, ArC*H*), 6.83-7.06 (m, 2H, ArC*H*), 3.68 (d, 2H, *J* = 9 Hz, CH_2), 2.91-2.98 (m, 1H, C*H*), 1.28 (d, 3H, *J* = 6 Hz, CH_3) ppm. The obtained analytical data is consistent with those previously reported in the literature.[7]

2-(thiophen-2-yl)propan-1-ol (7i): Prepared by following the general experimental procedure with:

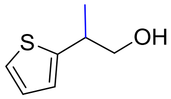

1 (2.48 mg, 0.5 mol%), 2-(thiophen-2-yl)ethan-1-ol **6i** (128.2 mg, 1 mmol), NaOMe (108.04 mg, 2 mmol), MeOH (1 mL). Yield was determined by ^{1}H NMR spectrum using

mesitylene (122 mg, 1.02 mmol) as an internal standard ($\delta_{Mesitylene(standard)}$ = 6.82 (s, 3H), $\delta_{product}$= 1.37 (d, 3H)).

^{1}H NMR (300 MHz, $CDCl_3$, 298 K) δ = δ 7.20 (dd, 1H, *J* = 5.02, 1.20, ArC*H*), 6.97-6.99 (m, 1H, ArC*H*), 6.90-6.91 (m, 1H, ArC*H*), 3.70 (dd, 2H, *J* = 6.39, 2.46, CH_2), 3.21-3.28 (m, 1H, C*H*), 1.37 (d, 3H, *J* = 6.96 Hz, CH_3), 1.36.[8]

2-(1H-indol-3-yl)propan-1-ol (7j): Prepared by following the general experimental procedure with:

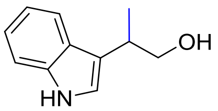

1 (2.48 mg, 0.5 mol%), 2-(1H-indol-3-yl)ethan-1-ol **6j** (161.2 mg, 1 mmol), NaOMe (108.04 mg, 2 mmol), MeOH (1 mL). Yield was determined by ^{1}H NMR spectrum using mesitylene (120 mg, 1 mmol) as an internal standard ($\delta_{Mesitylene(standard)}$ = 6.83 (s, 3H), $\delta_{product}$=3.32 (sext, 1H)).

^{1}H NMR (300 MHz, $CDCl_3$, 298 K) δ = 8.06 (br. s, 1H, NH), 7.67 (dt, 1H, *J* = 7.82, 0.95 Hz, ArC*H*), 7.38 (dt, 1H, *J* = 8.13, 0.99 Hz, ArC*H*), 7.21 (ddd, 1H, *J* = 8.15, 6.99, 1.28 Hz, ArC*H*), 7.13 (ddd, 1H, *J* = 7.99, 7.01, 1.11 Hz, ArC*H*), 7.06 (br s, 1H, ArC*H*), 3.77-3.88 (m, 2H, CH_2), 3.32 (sext, 1H, *J* = 6.78 Hz, C*H*), 1.52 (br. s, 1H, O*H*), 1.41 (d, 3H, *J* = 7.06 Hz, CH_3). **^{13}C{^{1}H}-NMR** (75 MHz, $CDCl_3$, 298 K) δ = 136.71 (quat-*C*), 126.88 (quat-*C*), 122.37 (Ar*C*H), 121.37 (quat-*C*), 119.56 (Ar*C*H), 119.39 (Ar*C*H), 118.15 (Ar*C*H), 111.42 (Ar*C*H), 68.05 (CH_2), 34.07 (*C*H), 17.42 (CH_3). Isolated yield: 47%. The obtained analytical data is consistent with those previously reported in the literature.[8]

5. General procedure for the catalytic selective β-methylation of secondary alcohols

Mn-MACHO **1** (2.48 mg, 0.5 mol%) and NaOMe (216.08 mg, 4 mmol) were measured into a glass inlet equipped with a stirring bar inside a glovebox. The glass inlet was closed with a septum and transferred into the bottom part of the 10 mL steel autoclave, where it was opened under a stream of argon. After sealing, the autoclave was purged with argon three times. Secondary alcohol (1 mmol) and methanol (1 mL) were added at room temperature through a valve under argon. The autoclave was sealed and heated to 150 °C temperature. After 36 h, the autoclave was cooled to room temperature and slowly vented while stirring continued. Mesitylene was added as an internal standard to the reaction mixture that was then passed through a short path of acidic alumina before the composition was analyzed by NMR spectroscopy. The isolation of pure product was carried out using column chromatography over silica gel (100-200 mesh) using ethyl acetate/petroleum ether (12 : 88) mixture as eluent.

2-methyl-1-phenylpropan-1-ol (9a): Prepared by following the general experimental procedure with:

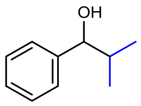

1 (2.48 mg, 0.5 mol%), 1-phenylethan-1-ol **8a** (122.2 mg, 1 mmol), NaOMe (216.08 mg, 4 mmol), MeOH (1 mL). Yield was determined by ^{1}H NMR spectrum using mesitylene (120 mg, 1 mmol) as an internal standard ($\delta_{Mesitylene(standard)}$ = 6.82 (s, 3H), $\delta_{product}$ = 0.81 (d, 3H)).

^{1}H NMR (400 MHz, $CDCl_3$, 298 K) δ = 7.27-7.38 (m, 5H, ArC*H*), 4.37 (d, 1H, *J* = 6.90 Hz, C*H*), 1.93-2.01 (sext, 1H, *J* = 6.77 Hz, C*H*), 1.90 (br. S, 1H, O*H*), 1.01 (d, 3H, *J* = 6.74 Hz, CH_3), 0.81 (d, 3H, *J* = 6.74 Hz, CH_3) ppm. **^{13}C{^{1}H}-NMR** (101 MHz, $CDCl_3$, 298 K) δ = 143.76 (quat-*C*), 128.31 (Ar*C*H), 127.53 (Ar*C*H), 126.69 (Ar*C*H), 80.17 (*C*H), 35.38 (*C*H), 19.12 (*C*H_3), 18.37 (*C*H_3). Isolated yield: 71%. The obtained analytical data is consistent with those previously reported in the literature.[3]

2-methyl-1-(naphthalen-2-yl)propan-1-ol (9b): Prepared by following the general experimental

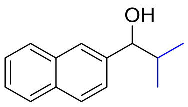

procedure with: **1** (2.48 mg, 0.5 mol%), 1-(naphthalen-2-yl)ethan-1-ol **8b** (172.2 mg, 1 mmol), NaOMe (216.08 mg, 4 mmol), MeOH (1 mL). Yield was determined by ^{1}H NMR spectrum using mesitylene (120 mg, 1 mmol) as an internal standard ($\delta_{Mesitylene(standard)}$ = 6.84 (s, 3H), $\delta_{product}$ = 4.54 (d, 1H)).

^{1}H NMR (400 MHz, $CDCl_3$, 298 K) δ = 7.81-7.85 (m, 3H, ArC*H*), 7.75-7.76 (m, 1H, ArC*H*), 7.45-7.50 (m, 3H, ArC*H*), 4.54 (d, 1H, *J* = 6.84 Hz, C*H*), 2.02-2.16 (m, 1H, C*H*), 1.90 (br. s, 1H, O*H*), 1.05 (d, 3H, *J* = 6.69 Hz, CH_3), 0.84 (d, 3H, *J* = 6.80 Hz. CH_3) ppm. **^{13}C{^{1}H}-NMR** (101 MHz, $CDCl_3$, 298 K) δ = 141.24 (Ar*C*H), 133.29 (quat-*C*), 133.08 (Ar*C*H), 128.09 (Ar*C*H), 128.06 (Ar*C*H), 127.79 (Ar*C*H), 126.19 (Ar*C*H), 125.87 (Ar*C*H),

125.54 (Ar*C*H), 124.76 (quat-*C*), 80.30 (*C*H), 35.34 (*C*H), 19.29 (CH_3), 18.38 (CH_3). Isolated yield: 74%.The obtained analytical data is consistent with those previously reported in the literature.[9]

2-methyl-1-(*p*-tolyl)propan-1-ol (9c): Prepared by following the general experimental procedure with:

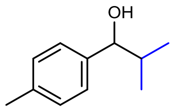

1 (2.48 mg, 0.5 mol%), 1-(*p*-tolyl)ethan-1-ol **8c** (136.1 mg, 1 mmol), NaOMe (216.08 mg, 4 mmol), MeOH (1 mL). Yield was determined by ^{1}H NMR spectrum using mesitylene (125 mg, 1.04 mmol) as an internal standard ($\delta_{Mesitylene(standard)}$ = 6.81 (s, 3H), $\delta_{product}$ = 1.01 (d, 3H)).

^{1}H NMR (400 MHz, $CDCl_3$, 298 K) δ = 7.20 (d, 2H, *J* = 8.09 Hz, ArC*H*), 7.15 (d, 2H, *J* = 7.92 Hz, ArC*H*), 4.32 (d, 1H, *J* = 6.98 Hz, C*H*), 2.35 (s, 3H, CH_3), 1.95 (sext, 1H, *J* = 6.74 Hz, C*H*), 1.76 (br. s, 1H, O*H*), 1.01 (d, 3H, *J* = 6.64 Hz, CH_3), 0.79 (d, 3H, *J* = 6.79 Hz, CH_3). **^{13}C{^{1}H}-NMR** (75 MHz, $CDCl_3$, 298 K) δ = 140.82 (quat-*C*), 137.17 (quat-*C*), 129.01 (ArCH), 126.63 (Ar*C*H), 80.10 (*C*H), 35.35 (*C*H), 21.26 (CH_3), 19.15 (CH_3), 18.51 (CH_3). Isolated yield: 73%. The obtained analytical data is consistent with those previously reported in the literature.[3]

2-methyl-1-(*o*-tolyl)propan-1-ol (9d): Prepared by following the general experimental procedure with:

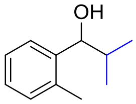

1 (2.48 mg, 0.5 mol%), 1-(*o*-tolyl)ethan-1-ol **8d** (136.1 mg, 1 mmol), NaOMe (216.08 mg, 4 mmol), MeOH (1 mL). Yield was determined by ^{1}H NMR spectrum using mesitylene (120 mg, 1 mmol) as an internal standard ($\delta_{Mesitylene(standard)}$ = 6.82 (s, 3H), $\delta_{product}$ = 1.05 (d, 3H)).

^{1}H NMR (300 MHz, $CDCl_3$, 298 K) δ = 7.53–7.12 (m, 4H, ArC*H*), 4.63 (d, 1H, *J* = 6 Hz, C*H*), 2.35 (s, 3H, CH_3), 2.04–1.93 (m, 1H, C*H*), 1.05 (d, 3H, *J* = 6 Hz, CH_3), 0.87 (d, 3H, *J* = 6 Hz, CH_3) ppm. The obtained analytical data is consistent with those previously reported in the literature.[10]

1-(4-methoxyphenyl)-2-methylpropan-1-ol (9e): Prepared by following the general experimental

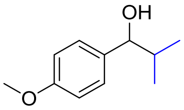

procedure with: **1** (2.48 mg, 0.5 mol%), 1-(4-methoxyphenyl)ethan-1-ol **8e** (152.2 mg, 1 mmol), NaOMe (216.08 mg, 4 mmol), MeOH (1 mL). Yield was determined by ^{1}H NMR spectrum using mesitylene (120 mg, 1 mmol) as an internal standard ($\delta_{Mesitylene(standard)}$ = 6.81 (s, 3H), $\delta_{product}$ = 4.30 (d, 1H)).

^{1}H NMR (400 MHz, $CDCl_3$, 298 K) δ = 7.23 (d, 2H, *J* = 8.62 Hz, ArC*H*), 6.87 (d, 2H, *J* = 8.66 Hz, ArC*H*), 4.30 (d, 1H, *J* = 7.13 Hz, C*H*), 3.81 (s, 3H, OCH_3), 1.89-1.97 (m, 1H, C*H*), 1.79 (br. s, 1H, O*H*), 1.01 (d, 3H, *J* = 6.64 Hz, CH_3), 0.77 (d, 3H, 6.79 Hz, CH_3). **^{13}C{^{1}H}-NMR** (101 MHz, $CDCl_3$, 298 K) δ = 159.06 (quat-*C*), 135.98 (quat-*C*), 127.84 (ArCH), 113.70 (Ar*C*H), 79.90 (*C*H), 55.40 (OCH_3), 35.42 (*C*H), 19.10 (CH_3), 18.65 (CH_3). Isolated yield: 51%. The obtained analytical data is consistent with those previously reported in the literature.[11]

1-(4-fluorophenyl)-2-methylpropan-1-ol (9f): Prepared by following the general experimental procedure with: **1** (2.48 mg, 0.5 mol%), 1-(4-fluorophenyl)ethan-1-ol **8f** (140.2 mg, 1 mmol), NaOMe (216.1 mg, 4 mmol), MeOH (1 mL). Reaction time: 42 h. Yield was determined by ^{1}H NMR spectrum using mesitylene (117 mg, 0.97 mmol) as an internal standard ($\delta_{Mesitylene(standard)}$ = 6.82 (s, 3H), $\delta_{product}$ = 4.31 (d, 1H)).

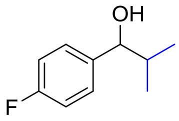

^{1}H NMR (400 MHz, $CDCl_3$, 298 K) δ = 7.24 (d, 2H, *J* = 8.64 Hz, ArC*H*), 6.88 (d, 2H, *J* = 8.60 Hz, ArC*H*), 4.29 (d, 1H, *J* = 7.15 Hz, C*H*), 1.86-1.99 (m, 1H, C*H*), 1.02 (d, 3H, *J* = 6.61 Hz, CH_3), 0.78 (d, 3H, *J* = 6.76 Hz, CH_3). The obtained analytical data is consistent with those previously reported in the literature.[12]

1-(furan-2-yl)-2-methylpropan-1-ol (9g): Prepared by following the general experimental procedure with: **1** (2.48 mg, 0.5 mol%), 1-(furan-2-yl)ethan-1-ol **8g** (112.10 mg, 1 mmol), NaOMe (216.08 mg, 4 mmol), MeOH (1 mL). Yield was determined by ^{1}H NMR spectrum using mesitylene (116 mg, 0.96 mmol) as an internal standard ($\delta_{Mesitylene(standard)}$ = 6.81 (s, 3H), $\delta_{product}$ = 0.86 (d, 3H).

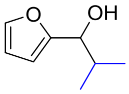

^{1}H NMR (400 MHz, $CDCl_3$, 298 K) δ = 7.37 (br. s, 1H, ArC*H*), 6.33 (dd, 1H, *J* = 3.22 Hz, 1.83 Hz, ArCH), 6.22 (d, 1H, *J* = 3.23 hz), 4.37 (d, 1H, *J* = 7.06 hz, CH), 2.06-2.15 (m, 1H, C*H*), 1.84 (br. s, 1H, O*H*), 1.02 (d, 3H, *J* = 6.75 Hz, CH_3), 0.86 (d, 3H, *J* = 6.75 Hz, CH_3). **^{13}C{^{1}H}-NMR** (101 MHz, $CDCl_3$, 298 K) δ = 156.28 (quat-*C*), 141.82 (Ar*C*H), 110.17 (Ar*C*H), 106.60 (Ar*C*H), 73.67 (*C*H), 33.49 (*C*H), 18.85 (*C*H_3), 18.36 (*C*H_3). Isolated yield: 55%. The obtained analytical data is consistent with those previously reported in the literature.[13]

1-cyclohexyl-2-methylpropan-1-ol (9h): Prepared by following the general experimental procedure with: **1** (2.48 mg, 0.5 mol%), 1-cyclohexylethan-1-ol **8h** (128.21 mg, 1 mmol), NaOMe (216.08 mg, 4 mmol), MeOH (1 mL). Yield was determined by ^{1}H NMR (400 MHz, $CDCl_3$, 298 K) spectrum using mesitylene (120 mg, 1 mmol) as an internal standard ($\delta_{Mesitylene(standard)}$ = 6.82 (s, 3H), $\delta_{product}$ = 3.05 (t, 1H). The obtained analytical data is consistent with those previously reported in the literature.[14]

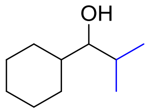

2-methyl-2,3-dihydro-1H-inden-1-ol (9i): Prepared by following the general experimental procedure with: **1** (2.48 mg, 0.5 mol%), 2,3-dihydro-1H-inden-1-ol **8i** (134.2 mg, 1 mmol), NaOMe (216.08 mg, 4 mmol), MeOH (1 mL). Yield was determined by ^{1}H NMR (400 MHz, $CDCl_3$, 298 K) spectrum using mesitylene (120 mg, 1 mmol) as an internal standard ($\delta_{Mesitylene(standard)}$ = 6.72 (s, 3H), $\delta_{product}$= 4.63 (d, 1H), $\delta_{product}$ = 4.91 (d, 1H)). The obtained analytical data is consistent with those previously reported in the literature.[15]

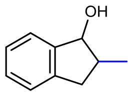

2-methyl-1,2,3,4-tetrahydronaphthalen-1-ol (9j): Prepared by following the general experimental

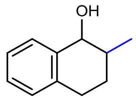

procedure with: **1** (2.48 mg, 0.5 mol%), 1,2,3,4-tetrahydronaphthalen-1-ol **8j** (148.2 mg, 1 mmol), NaOMe (216.08 mg, 4mmol), MeOH (1 mL). Yield was determined by ^{1}H NMR (300 MHz, $CDCl_3$, 298 K) spectrum using mesitylene (120 mg, 1 mmol) as an internal standard ($\delta_{Mesitylene(standard)}$ = 6.82 (s, 3H), $\delta_{product}$ = 4.33 (d, 1H), $\delta_{product}$ = 4.56 (d, 1H)). The obtained analytical data is consistent with those previously reported in the literature.[16]

6-methyl-6,7,8,9-tetrahydro-5H-benzo[7]annulen-5-ol (9k): Prepared by following the general

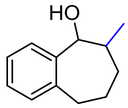

experimental procedure with: **1** (1.24 mg, 0.5 mol%), 6,7,8,9-tetrahydro-5H-benzo[7]annulen-5-ol **8k** (81.1 mg, 0.5 mmol), NaOMe (108.04 mg, 2 mmol), MeOH (0.5 mL). Yield was determined by ^{1}H NMR (300 MHz, $CDCl_3$, 298 K) spectrum using mesitylene (120 mg, 1 mmol) as an internal standard ($\delta_{Mesitylene(standard)}$ = 6.82 (s, 3H), $\delta_{product}$ = 4.60 (d, 1H), $\delta_{product}$ = 4.95 (d, 1H)). The obtained analytical data is consistent with those previously reported in the literature.[9, 15b]

6. General procedure for catalytic selective *β*-methylation of aliphatic alcohols

Mn-MACHO **1** (2.48 mg, 0.5 mol%) and NaOMe (108.04 mg, 2 mmol) were measured into a glass inlet equipped with a stirring bar inside a glovebox. The glass inlet was closed with a septum and transferred into the bottom part of the 10 mL steel autoclave, where it was opened under a stream of argon. After sealing, the autoclave was purged with argon three times. Aliphatic alcohol (1 mmol) and methanol (1 mL) were added at room temperature through a valve under argon. The autoclave was sealed and heated to 150 ^{o}C temperature. After the desired reaction time, the autoclave was cooled to room temperature and slowly vented while stirring continued. Mesitylene was added as an internal standard to the reaction mixture that was then passed through a short path of acidic alumina before the composition was analyzed by NMR spectroscopy. The isolation of pure product was carried out using column chromatography over silica gel (100-200 mesh) using ethyl acetate/petroleum ether (12 : 88) mixture as eluent.

2-methyl-3-phenylpropan-1-ol (11a): Prepared by following the general experimental procedure with:

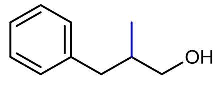

1 (2.48 mg, 0.5 mol%), 3-phenylpropan-1-ol **10a** (136.2 mg, 1 mmol), NaOMe (108.04 mg, 2 mmol), MeOH (1 mL), reaction time: 24 h. Yield was determined by ^{1}H NMR spectrum using mesitylene (120 mg, 1 mmol) as an internal standard ($\delta_{Mesitylene(standard)}$ = 6.82 (s, 3H), $\delta_{product}$ = 0.93 (d, 3H)).

^{1}H NMR (300 MHz, $CDCl_3$, 298 K) δ = 7.16-7.32 (m, 5H, ArC*H*), 3.51 (dq, 2H, *J* = 10.57 Hz, 5.95 Hz, CH_2), 2.77 (dd, 1H, *J* = 13.42 Hz, 6.33 Hz, C*H*), 2.43 (dd, 1H, *J* = 13.41 Hz, 8.04 Hz, C*H*), 1.90-2.01 (m, 1H, C*H*), 1.59 (s, 1H, O*H*), 0.93 (d, 3H, *J* = 6.75 Hz, CH_3). **^{13}C{^{1}H}-NMR** (101 MHz, $CDCl_3$, 298 K) δ = 140.75 (quat-*C*), 129.27 (Ar*C*H), 128.39 (Ar*C*H), 126.00 (Ar*C*H), 67.79 (CH_2), 39.84 (CH_2), 37.92 (*C*H), 16.60 (CH_3). Isolated yield: 66%.The obtained analytical data is consistent with those previously reported in the literature.[17]

3-(4-chlorophenyl)-2-methylpropan-1-ol (11b): Prepared by following the general experimental

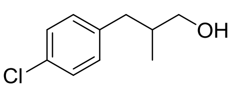

procedure with: **1** (2.48 mg, 0.5 mol%), 3-(4-chlorophenyl)propan-1-ol **10b** (170.6 mg, 1 mmol), NaOMe (108.04 mg, 2 mmol), MeOH (1 mL), reaction time: 14 h. Yield was determined by ^{1}H NMR spectrum using mesitylene (120 mg, 1 mmol) as an internal standard ($\delta_{Mesitylene(standard)}$ = 6.72 (s, 3H), $\delta_{product}$ = 0.83 (d, 3H)).

^{1}H NMR (400 MHz, $CDCl_3$, 298 K) δ = 7.12-7.24 (m, 3H, ArC*H*), 7.01-7.05 (m, 1H, ArC*H*), 3.38-3.46 (m, 2H, CH_2), 2.68 (dd, 1H, *J* = 13.50 Hz, 6.17 Hz, C*H*), 2.32 (dd, 1H, *J* = 13.49 Hz, 8.13 Hz, C*H*), 1.83-1.87 (m, 1H,

C*H*), 1.42 (br. s, 1H, O*H*), 0.83 (d, 3H, *J* = 6.74 Hz, C*H*$_3$). **^{13}C{^{1}H}-NMR** (75 MHz, $CDCl_3$, 298 K) δ = 139.19 (quat-*C*), 130.61 (Ar*C*H), 130.27 (quat-*C*), 128.49 (Ar*C*H), 67.55 (*C*H$_2$), 39.05 (*C*H$_2$), 37.82 (*C*H), 16.43 (*C*H$_3$). Isolated yield: 60%. The obtained analytical data is consistent with those previously reported in the literature.[18]

2-methyl-4-phenylbutan-1-ol (11c): Prepared by following the general experimental procedure with:

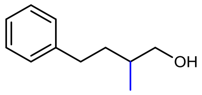

1 (2.48 mg, 0.5 mol%), 4-phenylbutan-1-ol **10c** (150.2 mg, 1 mmol), NaOMe (108.04 mg, 2 mmol), MeOH (1 mL), reaction time: 24 h. Yield was determined by ^{1}H NMR spectrum using mesitylene (120 mg, 1 mmol) as an internal standard ($\delta_{\text{Mesitylene(standard)}}$ = 6.82 (s, 3H), δ_{product} = 1.0 (d, 3H)).

^{1}H NMR (300 MHz, $CDCl_3$, 298 K) δ = 7.17-7.32 (m, 5H, ArC*H*), 3.54- 3.46 (m, 2H, C*H*$_2$), 2.77-2.58 (m, 2H, C*H*$_2$), 1.65-1.82 (m, 2H, C*H*$_2$), 1.49-1.41 (m, 1H, C*H*), 1.00 (d, *J* = 6.7 Hz, 3H, C*H*$_3$) ppm. The obtained analytical data is consistent with those previously reported in the literature.[19]

2-methyl-5-phenylpentan-1-ol (11d): Prepared by following the general experimental procedure with:

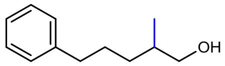

1 (2.48 mg, 0.5 mol%), 5-phenylpentan-1-ol **10d** (164.2 mg, 1 mmol), NaOMe (108.04 mg, 2 mmol), MeOH (1 mL) , reaction time: 24 h. Yield was determined by ^{1}H NMR spectrum using mesitylene (120 mg, 1 mmol) as an internal standard ($\delta_{\text{Mesitylene(standard)}}$ = 6.72 (s, 3H), δ_{product} = 0.83 (d, 3H)).

^{1}H NMR (400 MHz, $CDCl_3$, 298 K) δ = 6.72-7.21 (m, 5H, ArC*H*), 3.30-3.43 (m, 2H, C*H*$_2$), 2.50-2.56 (m, 2H, C*H*$_2$), 1.48-1.62 (m, 3H, C*H*$_2$ & C*H*), 1.28-1.42 (m, 2H, C*H*$_2$), 0.83 (d, 3H, *J* = 6 Hz, C*H*$_3$). The obtained analytical data is consistent with those previously reported in the literature.[20]

2-methylpropan-1-ol (11e): Prepared by following the general experimental procedure with:

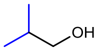

1 (4.95 mg, 0.5 mol%), ethanol **10e** (92.1 mg, 2 mmol), NaOMe (216.08 mg, 4 mmol), MeOH (1 mL), reaction time: 36 h. Yield was determined by ^{1}H NMR spectrum using mesitylene (170 mg, 1.41 mmol) as an internal standard ($\delta_{\text{Mesitylene(standard)}}$ = 6.82 (s, 3H), δ_{product} =0.94 (d, 6H)).

^{1}H NMR (300 MHz, $CDCl_3$, 298 K) δ = 3.42 (d, 2H, *J* = 6 Hz, C*H*$_2$), 1.85-1.71 (m, 1H, C*H*), 0.94 (d, 6H. *J* = 6 Hz, C*H*$_3$) ppm. The obtained analytical data is consistent with those previously reported in the literature.[21]

2-methylbutan-1-ol (11f): Prepared by following the general experimental procedure with:

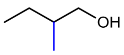

1 (2.48 mg, 0.5 mol%), 1-butanol **10f** (74.1 mg, 1 mmol), NaOMe (108.04 mg, 2 mmol),

MeOH (1 mL), reaction time: 36 h. Yield was determined by ^{1}H NMR spectrum using mesitylene (120 mg, 1 mmol) as an internal standard ($\delta_{Mesitylene(standard)}$ = 6.82 (s, 3H), $\delta_{product}$ =3.42-3.55 (m, 2H)).

^{1}H NMR (300 MHz, $CDCl_3$, 298 K) δ = 3.48 (m, 2H, CH_2), 1.61-1.51 (m, 1H, C*H*), 1.51-1.38 (m, 1H, CH_2), 0.98-0.92 (m, 6H, CH_3). The obtained analytical data is consistent with those previously reported in the literature.[22]

2-methylpentan-1-ol (11g): Prepared by following the general experimental procedure with:

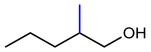

1 (2.48 mg, 0.5 mol%), 1-pentanol **10g** (88.2 mg, 1 mmol), NaOMe (108.04 mg, 2 mmol), MeOH (1 mL), reaction time: 36 h. Yield was determined by ^{1}H NMR spectrum using mesitylene (120 mg, 1 mmol) as an internal standard ($\delta_{Mesitylene(standard)}$ = 6.82 (s, 3H), $\delta_{product}$ = 3.48 (d, 1H)).

^{1}H NMR (300 MHz, $CDCl_3$, 298 K) δ = 3.48 (dd, 1H, *J* = 10.46, 5.81 Hz, C*H*), 3.37 (dd, 1H, *J* = 10.53, 6.57 Hz, 1H), 1.89-1.98 (m, 1H, O*H*), 1.53-1.68 (m, 1H, C*H*), 1.23-1.40 (m, 3H, CH_2 & C*H*), 1.05-1.11 (m, 1H, C*H*), 0.85-0.90 (m, 6H, CH_3). **^{13}C{^{1}H}-NMR** (75 MHz, $CDCl_3$, 298 K) δ = 68.42 (*C*H_2), 35.58 (*C*H), 35.53 (*C*H_2), 20.18 (*C*H_2), 16.64 (*C*H_3), 14.43 (*C*H_3). Isolated yield: 43%.The obtained analytical data is consistent with those previously reported in the literature.[23]

2-methylhexan-1-ol (11h): Prepared by following the general experimental procedure with:

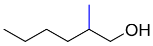

1 (2.48 mg, 0.5 mol%), 1-hexanol **10h** (102.2 mg, 1 mmol), NaOMe (108.04 mg, 2 mmol), MeOH (1 mL), reaction time: 24 h. Yield was determined by ^{1}H NMR spectrum using mesitylene (120 mg, 1 mmol) as an internal standard ($\delta_{Mesitylene(standard)}$ = 6.72 (s, 3H), $\delta_{product}$ = 3.29-3.44 (dq, 2H)).

^{1}H NMR (300 MHz, $CDCl_3$, 298 K) δ = 3.29-3.44 (dq, 2H, *J*= 30, 6 Hz, CH_2), 1.14-1.53 (m, 7H, CH_2 & C*H*), 0.79-0.86 (m, 6H, CH_3). The obtained analytical data is consistent with those previously reported in the literature.[24]

2-methyloctan-1-ol (11i): Prepared by following the general experimental procedure with:

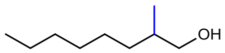

1 (2.48 mg, 0.5 mol%), 1-octanol **10i** (130.2 mg, 1 mmol), NaOMe (108.04 mg, 2 mmol), MeOH (1 mL), reaction time: 24 h. Yield was determined by ^{1}H NMR spectrum using mesitylene (120 mg, 1 mmol) as an internal standard ($\delta_{Mesitylene(standard)}$ = 6.73 (s, 3H), $\delta_{product}$ = 3.31-3.46 (dq, 2H)).

^{1}H NMR (300 MHz, $CDCl_3$, 298 K) δ = 3.44 (dd, 1H, *J* = 10.43 hz, 6.47 Hz, C*H*), 3.35 (dd, 1H, *J* = 10.43 Hz, 6.47 Hz, C*H*), 1.48-1.56 (m, 1H, C*H*), 1.44 (br s, 1H, O*H*), 1.20-1.30 (m, 10H, CH_2), 0.80-0.85 (m, 6H, CH_3). **^{13}C{^{1}H}-NMR** (101 MHz, $CDCl_3$, 298 K) δ = 68.59 (*C*H_2), 35.91 (*C*H), 33.29 (*C*H_2), 32.00 (*C*H_2), 29.74 (*C*H_2),

27.08 (CH_2), 22.80 (CH_2), 16.72 (CH_3), 14.23 (CH_3). Isolated yield: 62%. The obtained analytical data is consistent with those previously reported in the literature.[25]

2-Methyldecan-1-ol (11j): Prepared by following the general experimental procedure with:

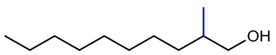

1 (2.48 mg, 0.5 mol%), 1-decanol **10j** (158.3 mg, 1 mmol), NaOMe (108.04 mg, 2 mmol), MeOH (1 mL), reaction time: 24 h. Yield was determined by ^{1}H NMR spectrum using mesitylene (120 mg, 1 mmol) as an internal standard ($\delta_{Mesitylene(standard)}$ = 6.72 (s, 3H), $\delta_{product}$ = 3.29-3.45 (dq, 2H)).

^{1}H NMR (300 MHz, $CDCl_3$, 298 K) δ = 3.29-3.45 (dq, 2H, *J* = 30, 6 Hz, CH_2), 1.43-1.51 (m, 1H, C*H*), 0.99-1.37 (m, 14H, CH_2), 0.78-0.84 (m, 6H, CH_3). The obtained analytical data is consistent with those previously reported in the literature.[26]

7. General procedure for the catalytic selective β-methylation of diols

Mn-MACHO **1** (2.48 mg, 0.5 mol%) and NaOMe were measured into a glass inlet equipped with a stirring bar inside a glovebox. The glass inlet was closed with a septum and transferred into the bottom part of the 10 mL steel autoclave, where it was opened under a stream of argon. After sealing, the autoclave was purged with argon three times. Diol (1 mmol) and methanol (1 mL) were added at room temperature through a valve under argon. The autoclave was sealed and heated to 150 ^{o}C temperature. After the desired reaction time, the autoclave was cooled to room temperature and slowly vented while stirring continued. Mesitylene was added as an internal standard to the reaction mixture which was further analyzed by NMR spectroscopy.

2-methylpropane-1,3-diol (11k): Prepared by following the general experimental procedure with:

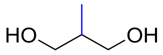

1 (2.48 mg, 0.5 mol%), propane-1,3-diol **10k** (76.0 mg, 1 mmol), NaOMe (108.04 mg, 2 mmol), MeOH (1 mL), reaction time: 36 h. Yield was determined by ^{1}H NMR spectrum using mesitylene (118 mg, 0.97 mmol) as an internal standard ($\delta_{Mesitylene(standard)}$ = 6.76 (s, 3H), $\delta_{product}$ = 0.84 (d, 3H)).

^{1}H NMR (300 MHz, DMSO-d_6, 298 K) δ = 3.44-3.51 (m, 4H, CH_2), 1.58-1.68 (m, 1H, C*H*), 0.83 (d, 3H, *J* = 9 Hz, CH_3). The obtained analytical data are consistent with those previously reported in the literature.[27]

2,5-dimethylhexane-1,6-diol (11l): Prepared by following the general experimental procedure with:

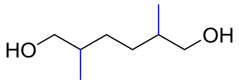

1 (2.48 mg, 0.5 mol%), hexane-1,6-diol **10l** (118.0 mg, 1 mmol), NaOMe (216.08 mg, 4 mmol), MeOH (1 mL), reaction time: 48 h. Yield was determined by ^{1}H NMR spectrum using mesitylene (111 mg, 0.93 mmol) as an internal standard ($\delta_{Mesitylene(standard)}$ = 6.77 (s, 3H), $\delta_{product}$ = 0.82 (d, 3H)).

^{1}H NMR (400 MHz, DMSO-d_6, 298 K) δ = 3.20-3.24 (m, 4H, CH_2), 1.30-1.43 (m, 4H, CH_2 & C*H*), 0.93-1.05 (m, 2H, CH_2), 0.82 (d, 3H, *J* = 3 Hz, CH_3), 0.80 (d, 3H, *J* = 3 Hz, CH_3). The obtained analytical data are consistent with those previously reported in the literature.[28]

2,9-dimethyldecane-1,10-diol (11m): Prepared by following the general experimental procedure with:

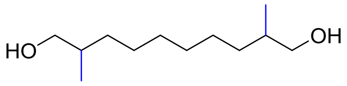

1 (2.48 mg, 0.5 mol%), decane-1,10-diol **10m** (174.0 mg, 1 mmol), NaOMe (216.08 mg, 4 mmol), MeOH (1 mL), reaction time: 48 h. Yield was determined by ^{1}H NMR spectrum using mesitylene (117 mg, 0.97 mmol) as an internal standard ($\delta_{Mesitylene(standard)}$ = 6.76 (s, 3H), $\delta_{product}$ = 0.82 (d, 6H)).

^{1}**H NMR** (400 MHz, DMSO-d_6, 298 K) δ = 3.14-3.28 (m, 4H, CH_2), 1.40-1.50 (m, 2H, C*H*), 1.13-1.28 (m, 10H, CH_2), 0.96-1.03 (m, 2H, CH_2), 0.82 (d, 6H, *J* = 6.64 Hz, CH_3). ^{13}**C{^{1}H}-NMR** (101 MHz, DMSO-d_6) δ = 66.73 (CH_2), 35.82 (*C*H), 33.37 (CH_2), 29.95 (CH_2), 26.98 (CH_2), 17.19 (CH_3). **HRMS** (ESI-) found $[M-H]^-$ = 201.185960; $C_{12}H_{25}O_2$ requires 201.186005. Isolated yield: 69%.

8. ^{1}H NMR spectra of reaction mixtures for β-methylated alcohols

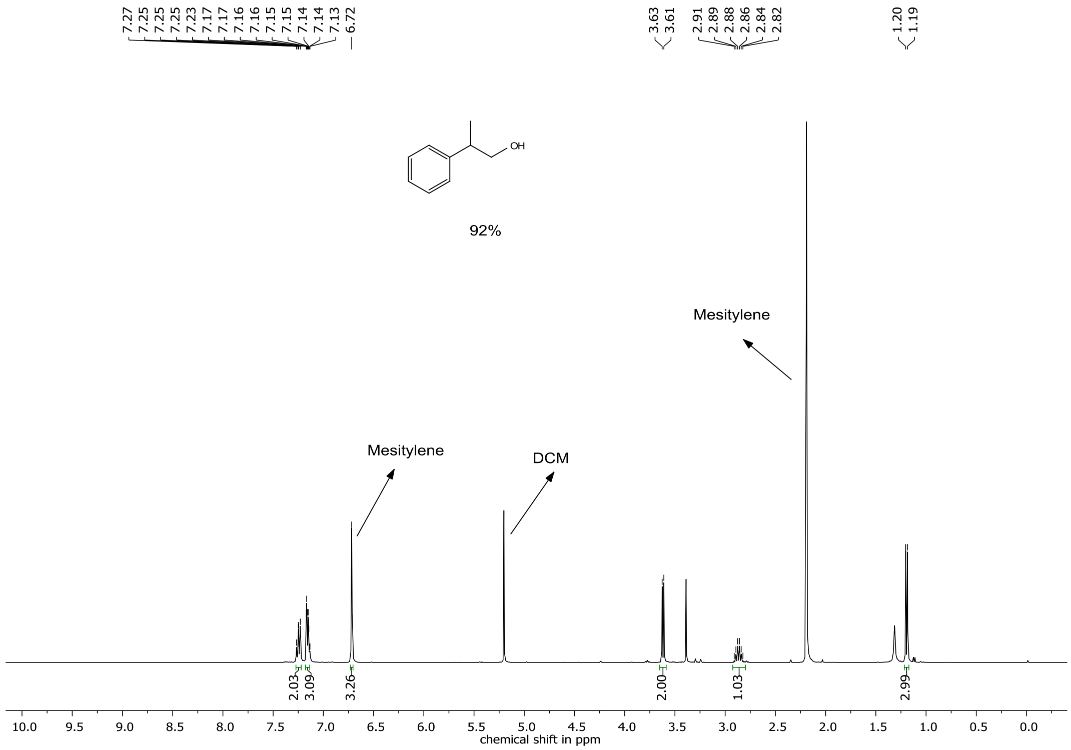

Figure S1: ^{1}H NMR (400 MHz, $CDCl_3$, 298 K) spectrum for 7a

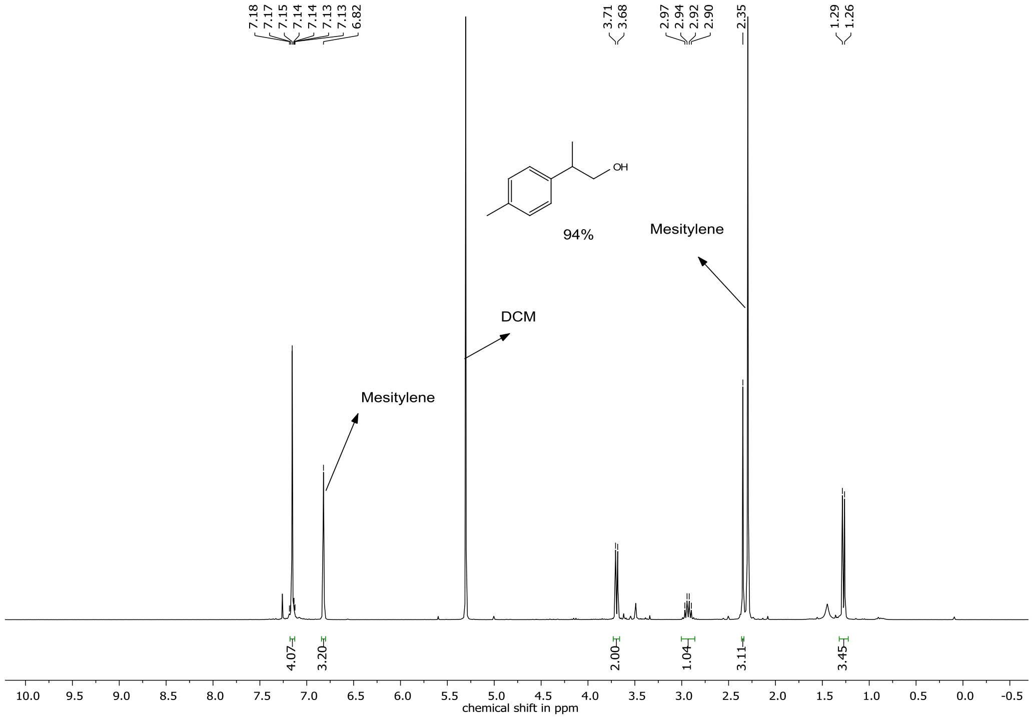

Figure S2: ^{1}H NMR (300 MHz, $CDCl_3$, 298 K) spectrum for 7b

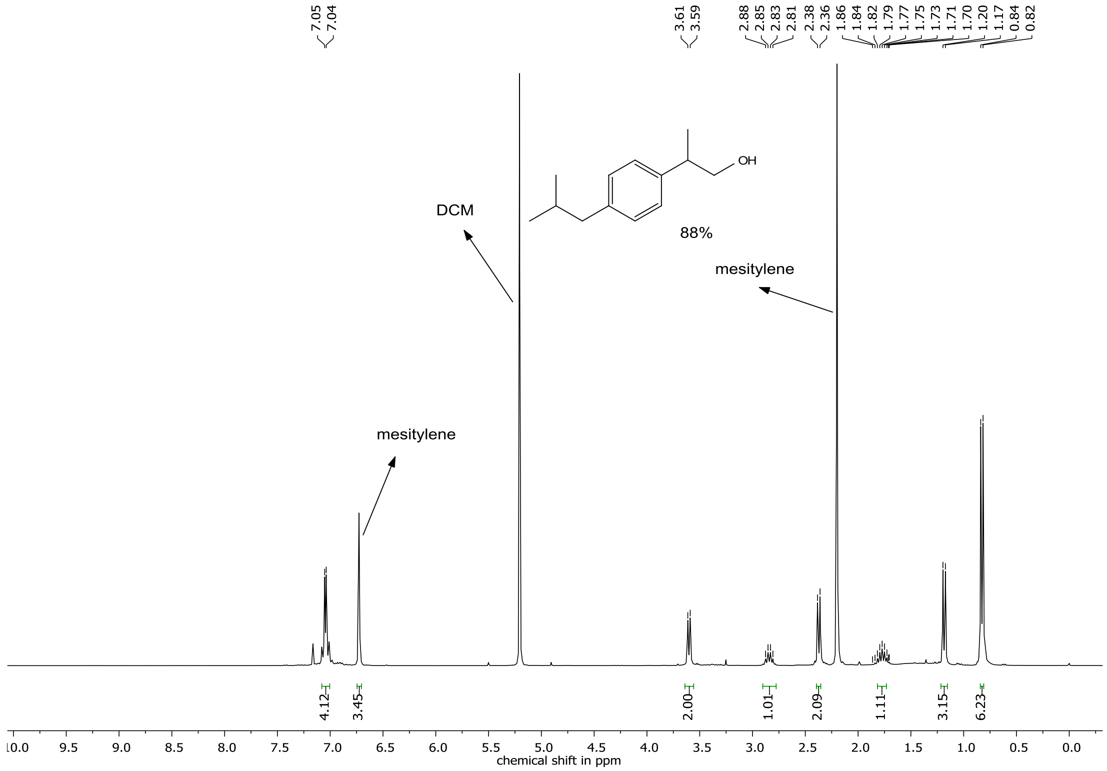

Figure S3: ^{1}H NMR (300 MHz, $CDCl_3$, 298 K) spectrum for 7c

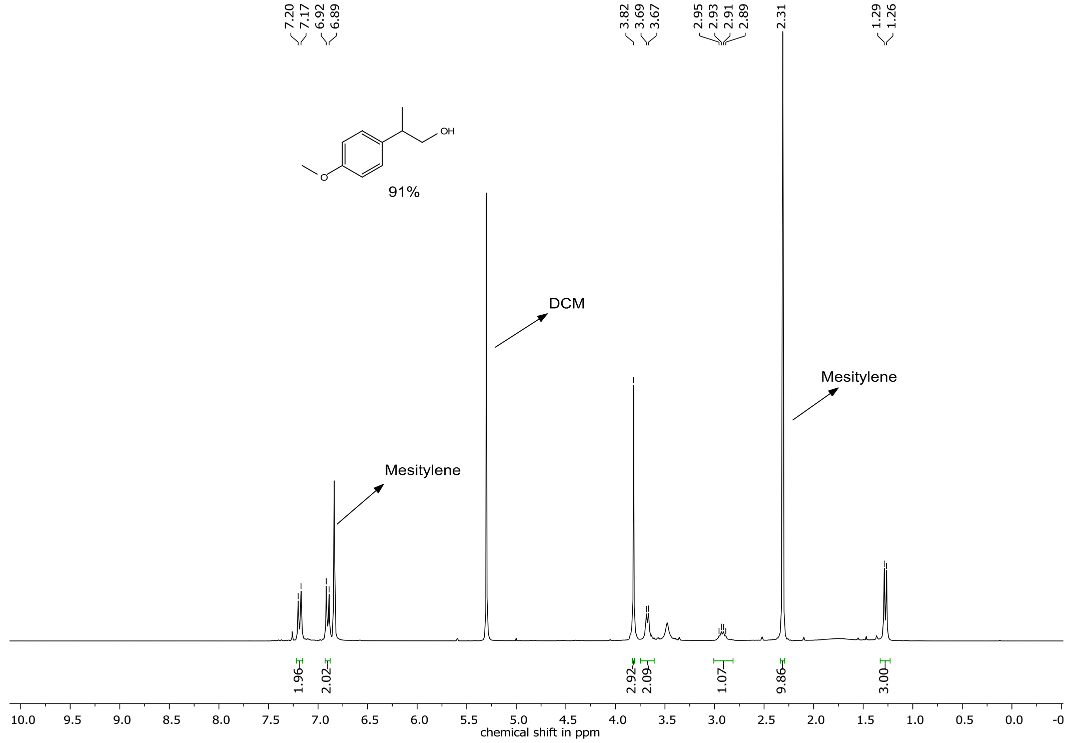

Figure S4: ^{1}H NMR (300 MHz, $CDCl_3$, 298 K) spectrum for 7d

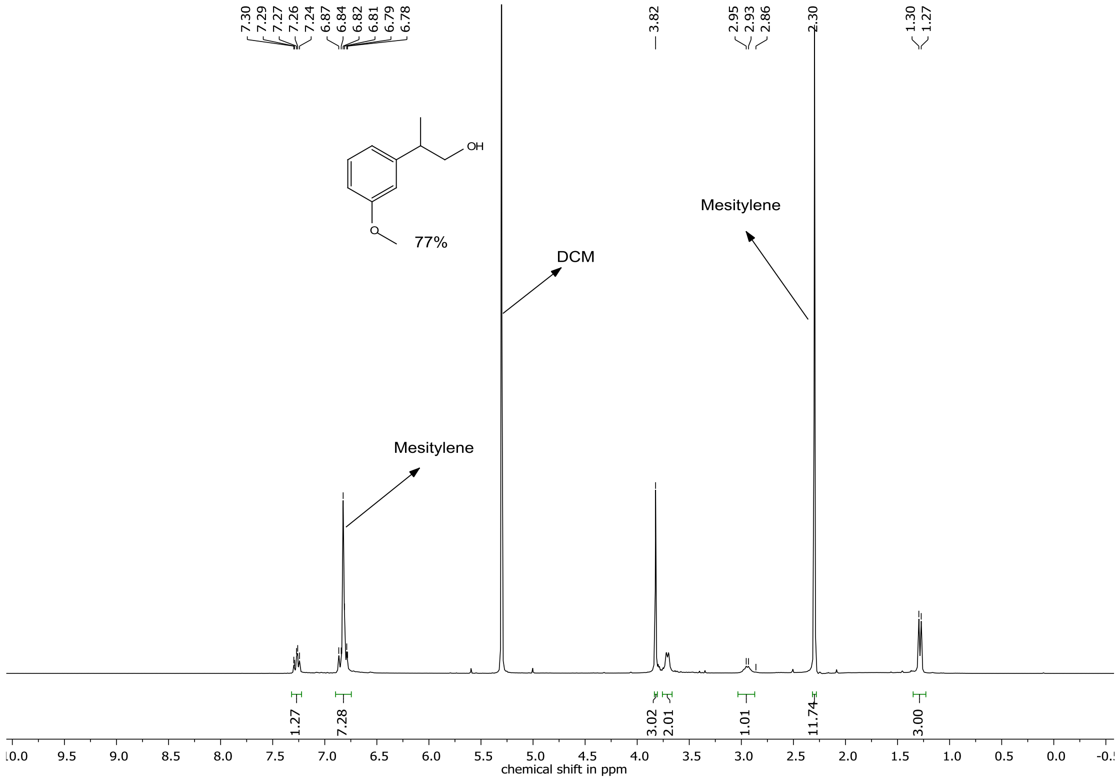

Figure S5: ^{1}H NMR (300 MHz, $CDCl_3$, 298 K) spectrum for 7e

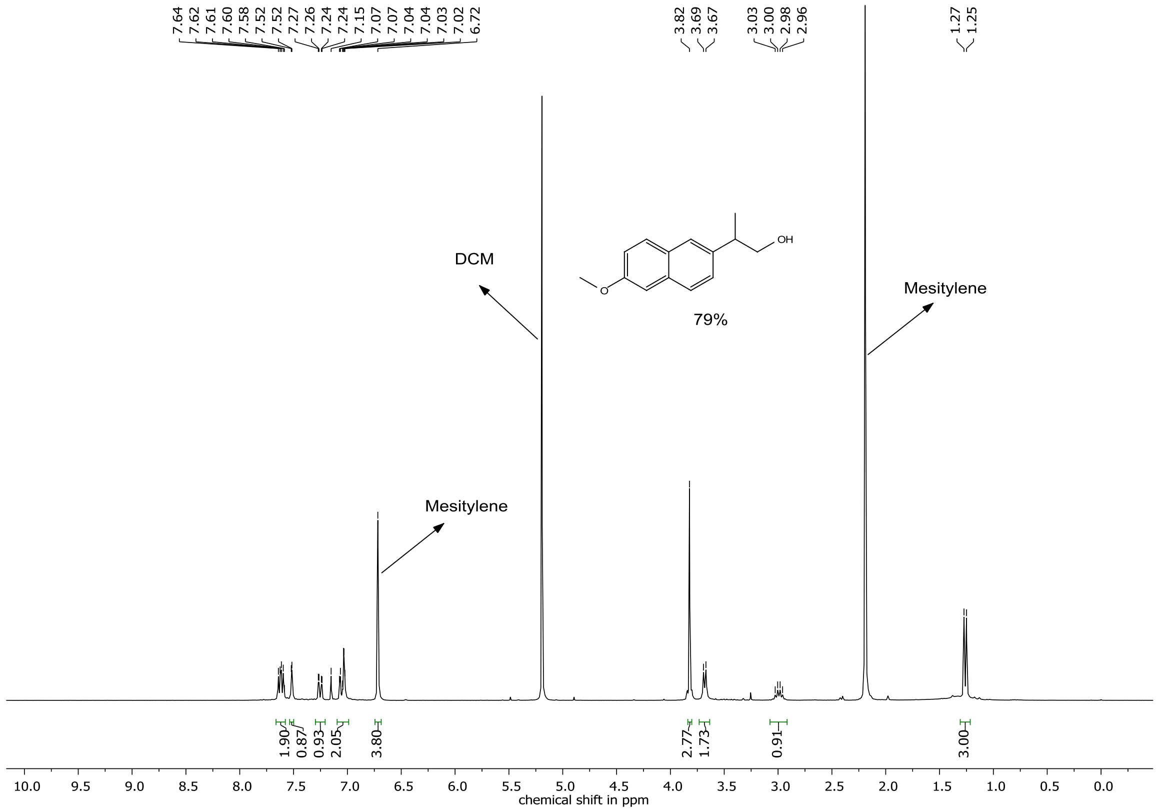

Figure S6: ^{1}H NMR (300 MHz, $CDCl_3$, 298 K) spectrum for 7f

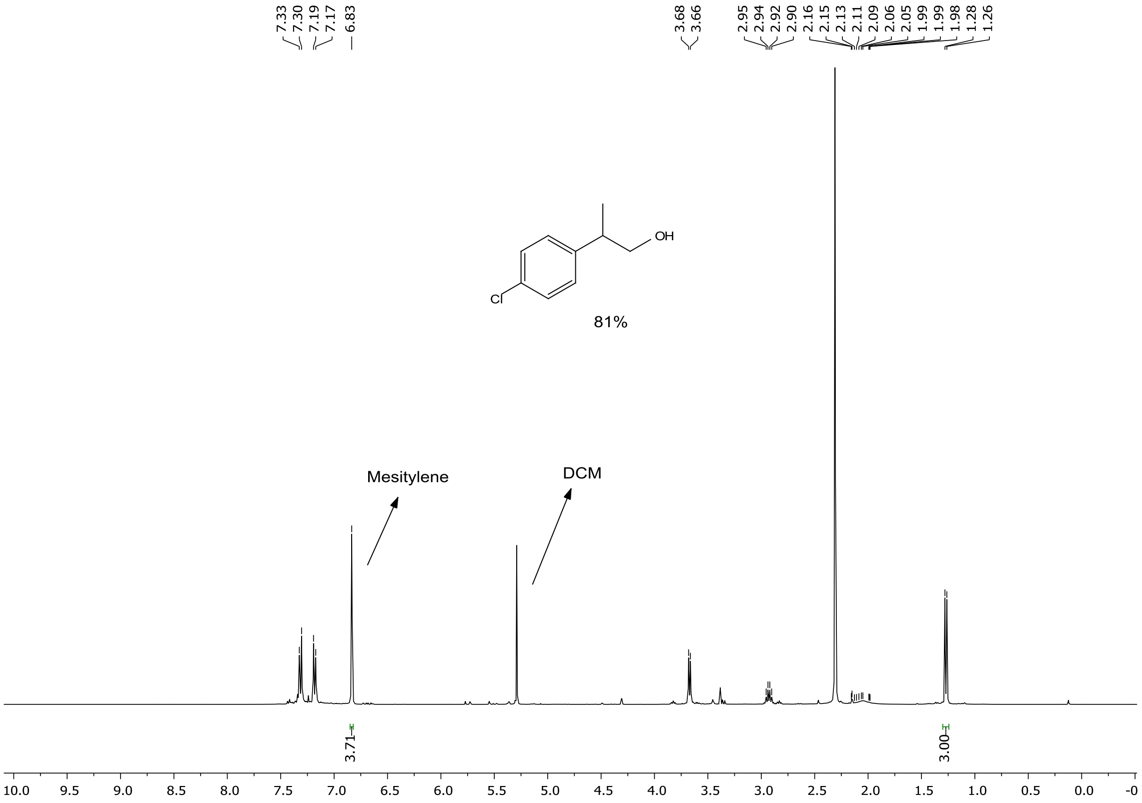

Figure S7: ^{1}H NMR (300 MHz, $CDCl_3$, 298 K) spectrum for 7g

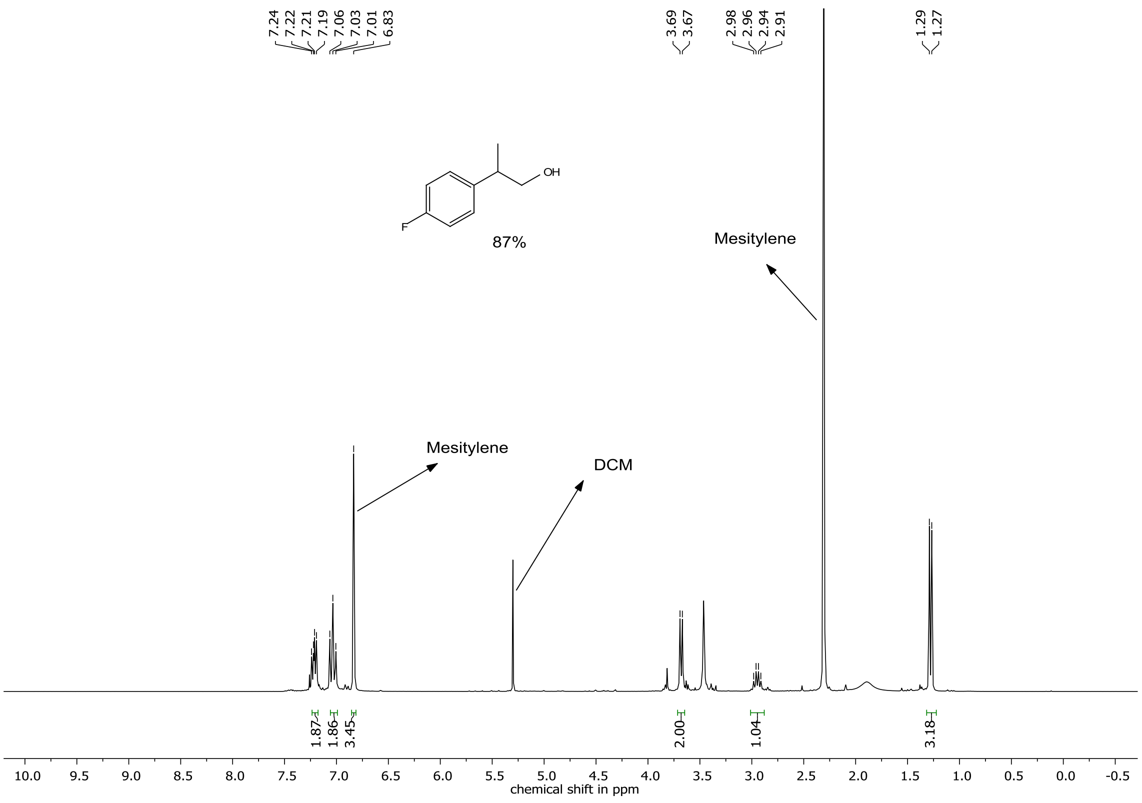

Figure S8: ^{1}H NMR (300 MHz, $CDCl_3$, 298 K) spectrum for 7h

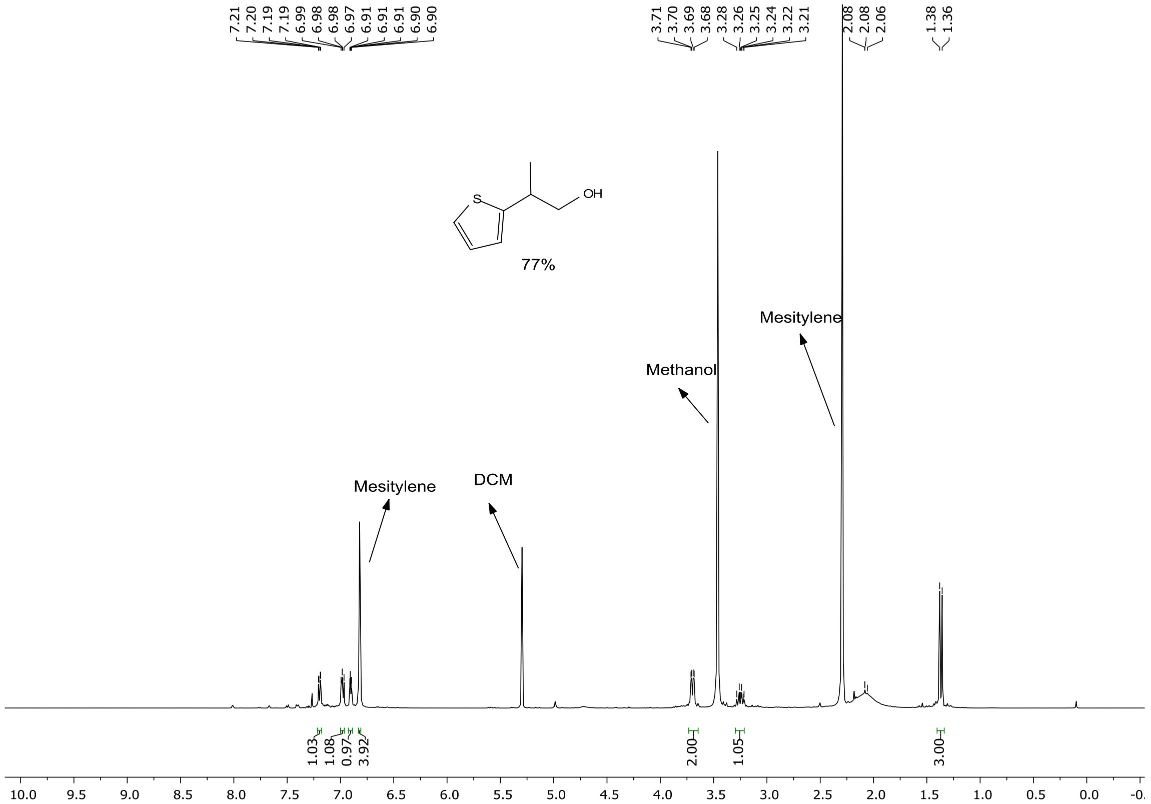

Figure S9: ^{1}H NMR (300 MHz, $CDCl_3$, 298 K) spectrum for 7i

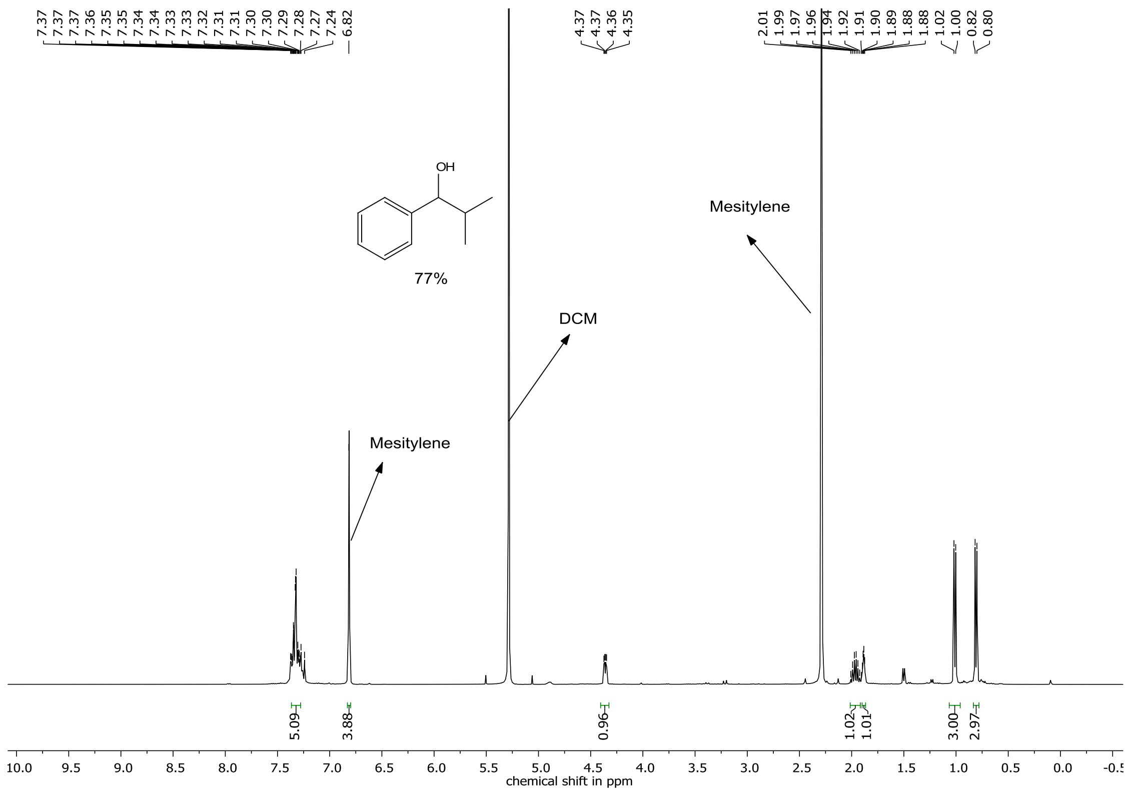

Figure S10: ^{1}H NMR (400 MHz, $CDCl_3$, 298 K) spectrum for 9a

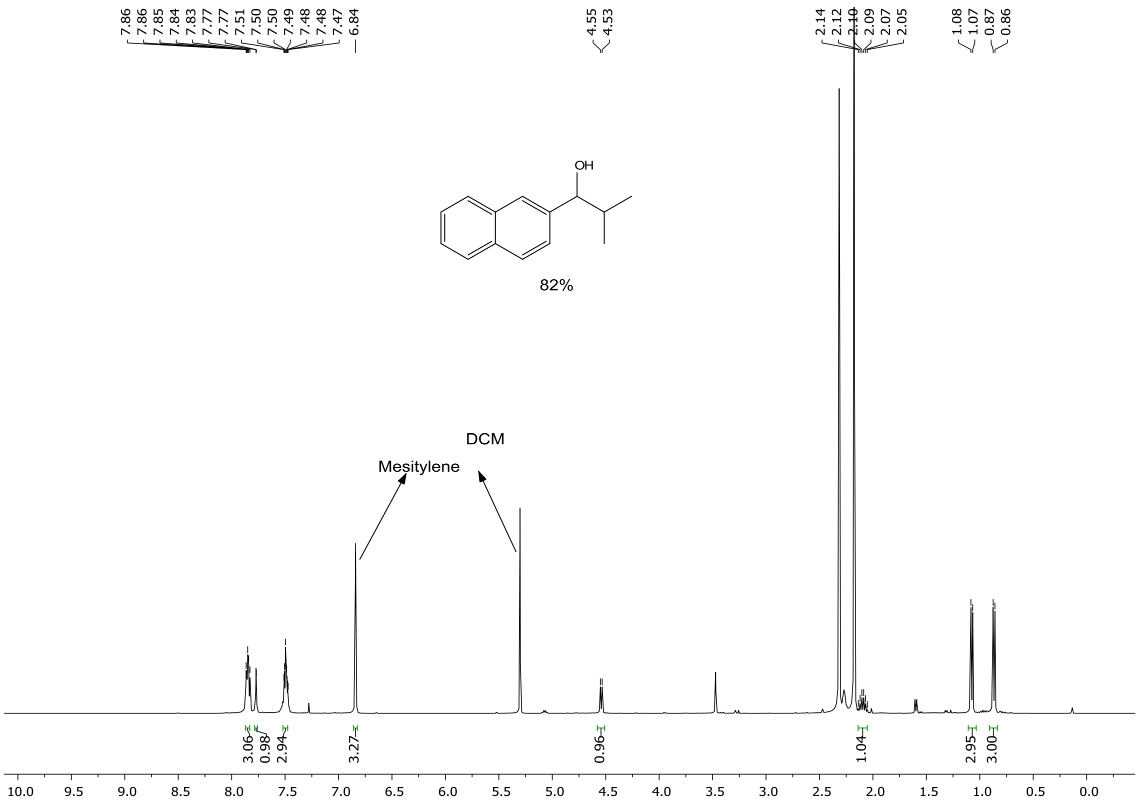

Figure S11: ^{1}H NMR (400 MHz, $CDCl_3$, 298 K) spectrum for 9b

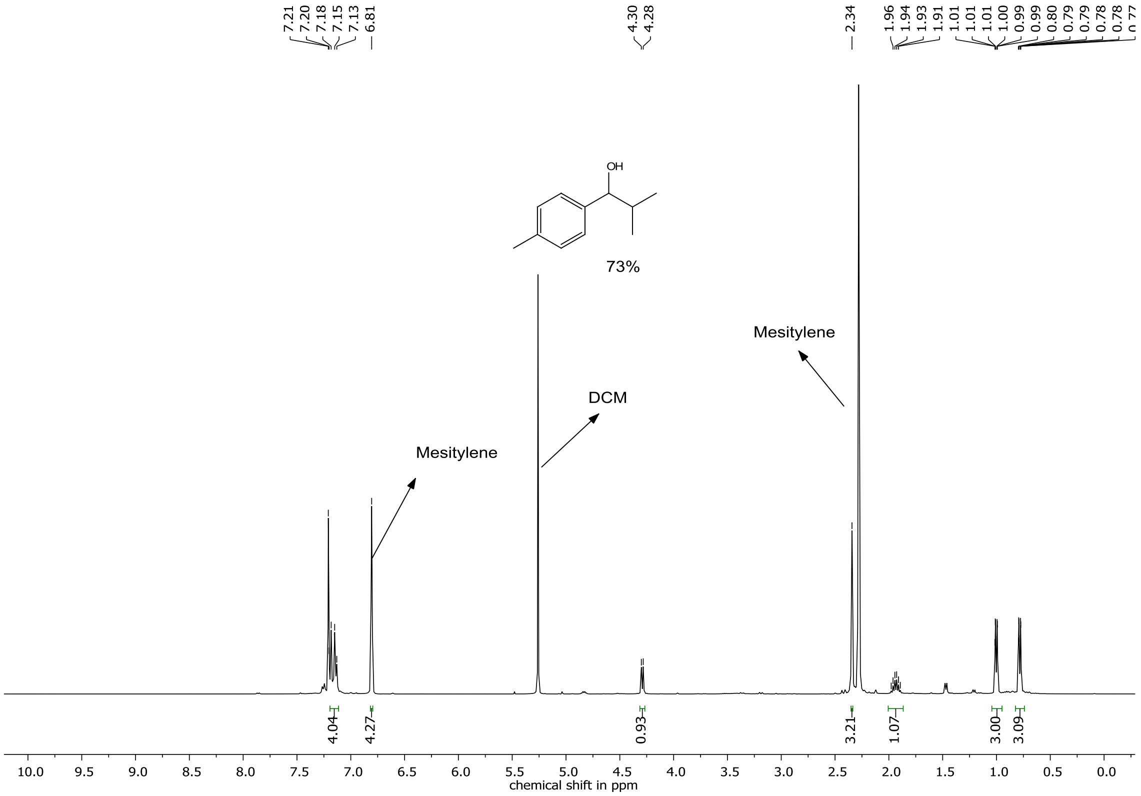

Figure S12: ^{1}H NMR (400 MHz, $CDCl_3$, 298 K) spectrum for 9c

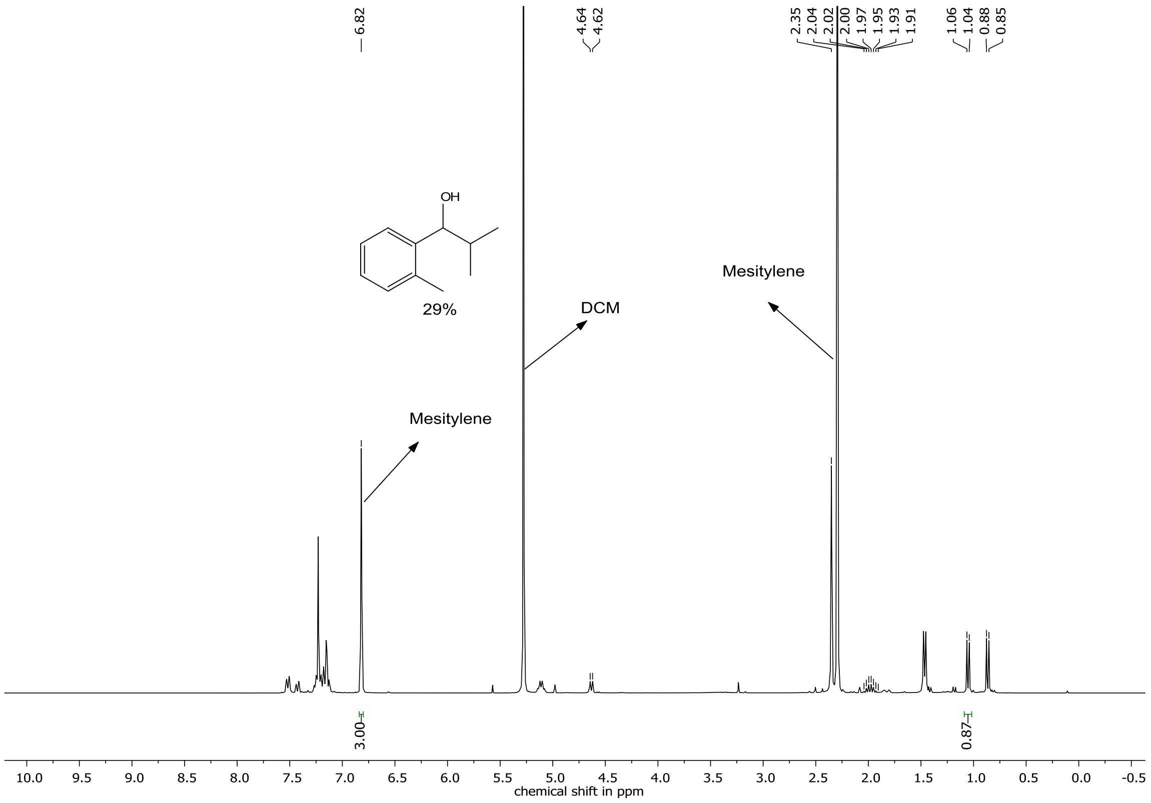

Figure S13: ^{1}H NMR (300 MHz, $CDCl_3$, 298 K) spectrum for 9d

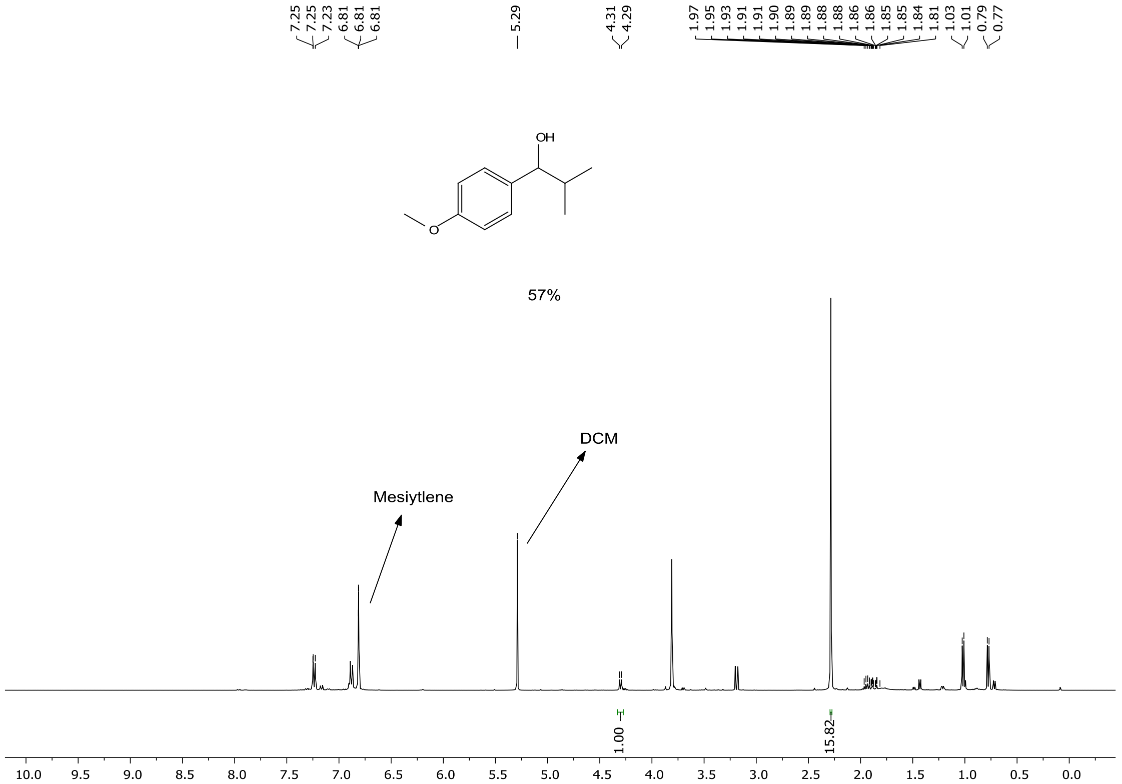

Figure S14: ^{1}H NMR (400 MHz, $CDCl_3$, 298 K) spectrum for 9e

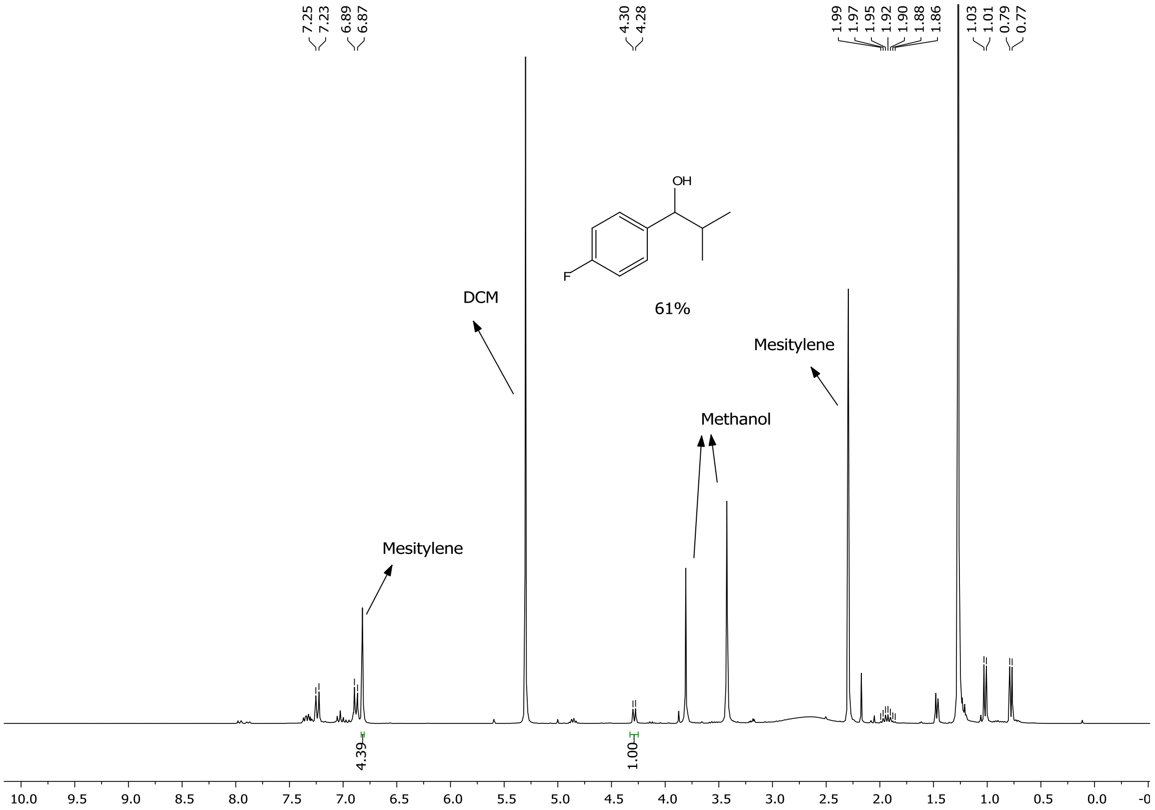

Figure S15: ^{1}H NMR (400 MHz, $CDCl_3$, 298 K) spectrum for 9f

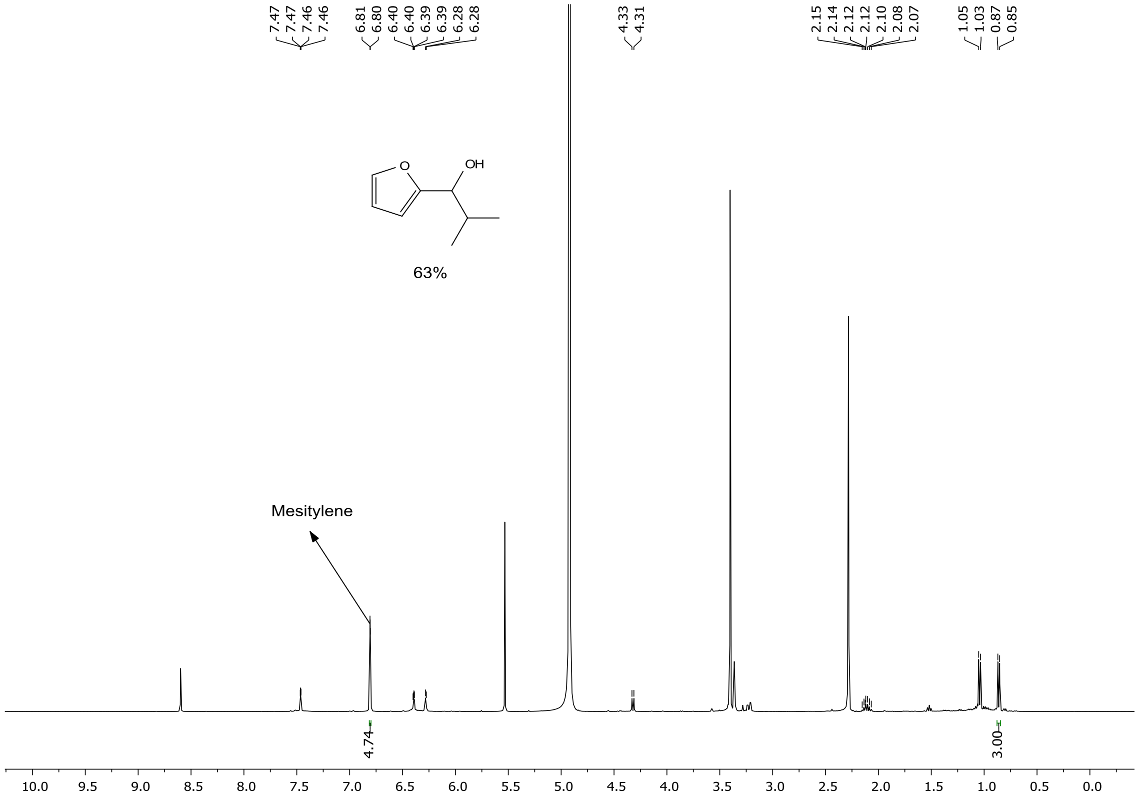

Figure S16: ^{1}H NMR (400 MHz, $CDCl_3$, 298 K) spectrum for 9g

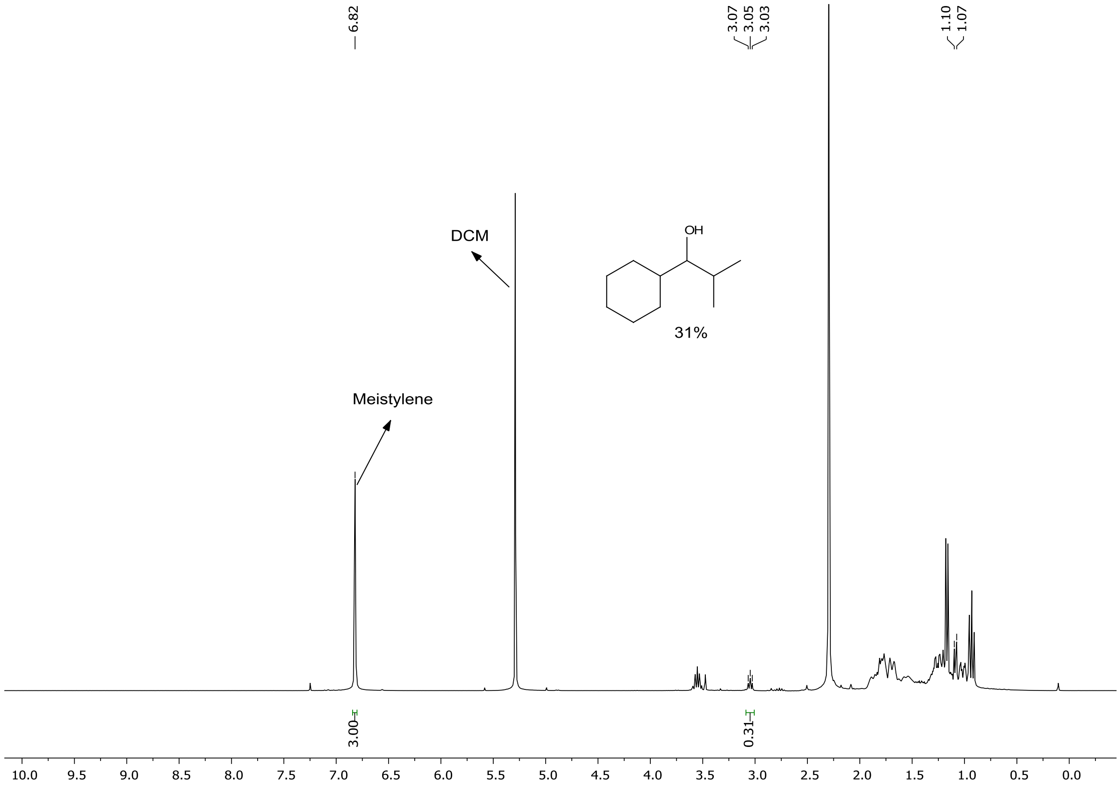

Figure S17: ^{1}H NMR (400 MHz, $CDCl_3$, 298 K) spectrum for 9h

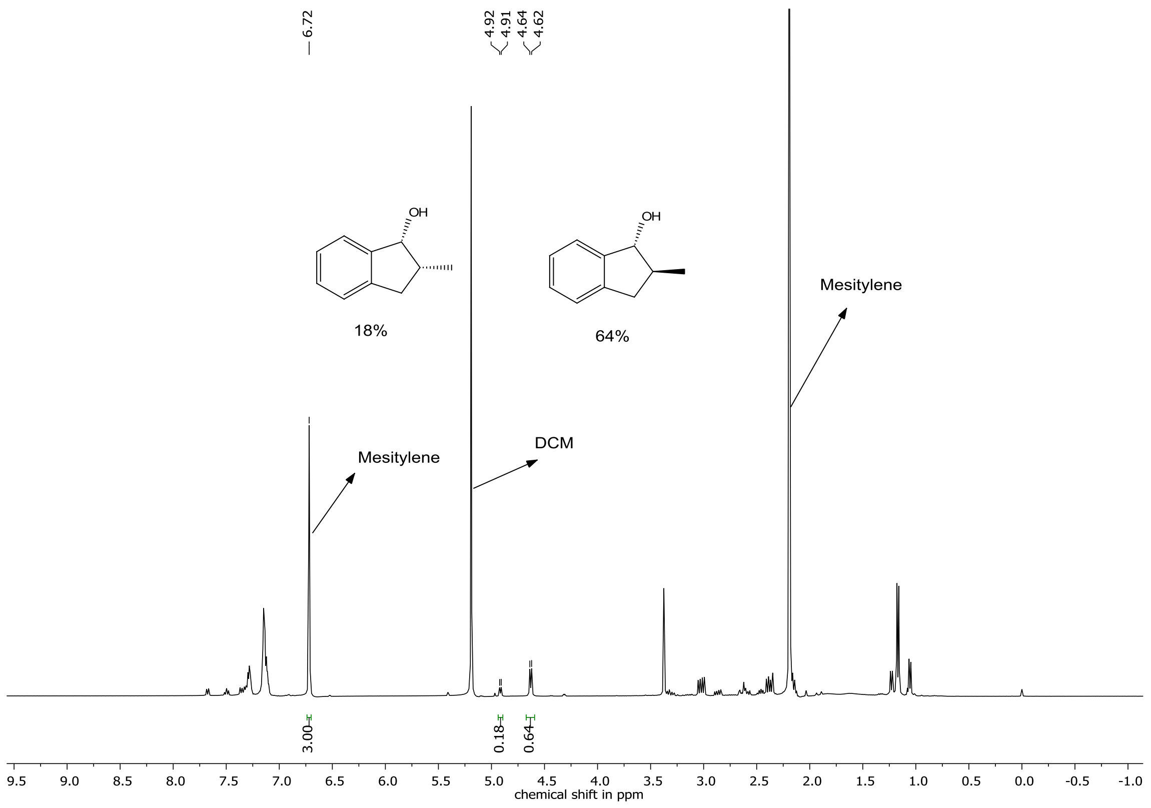

Figure S18: ^{1}H NMR (400 MHz, $CDCl_3$, 298 K) spectrum for 9i

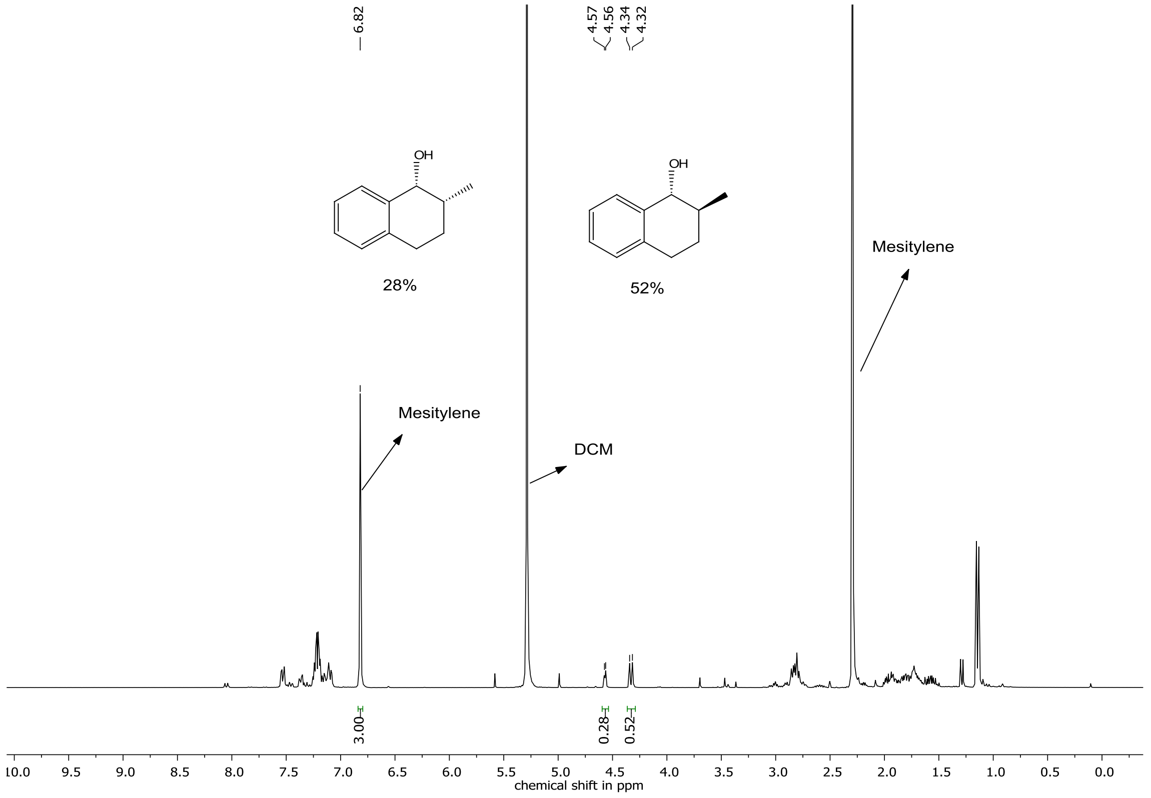

Figure S19: ^{1}H NMR (300 MHz, $CDCl_3$, 298 K) spectrum for 9j

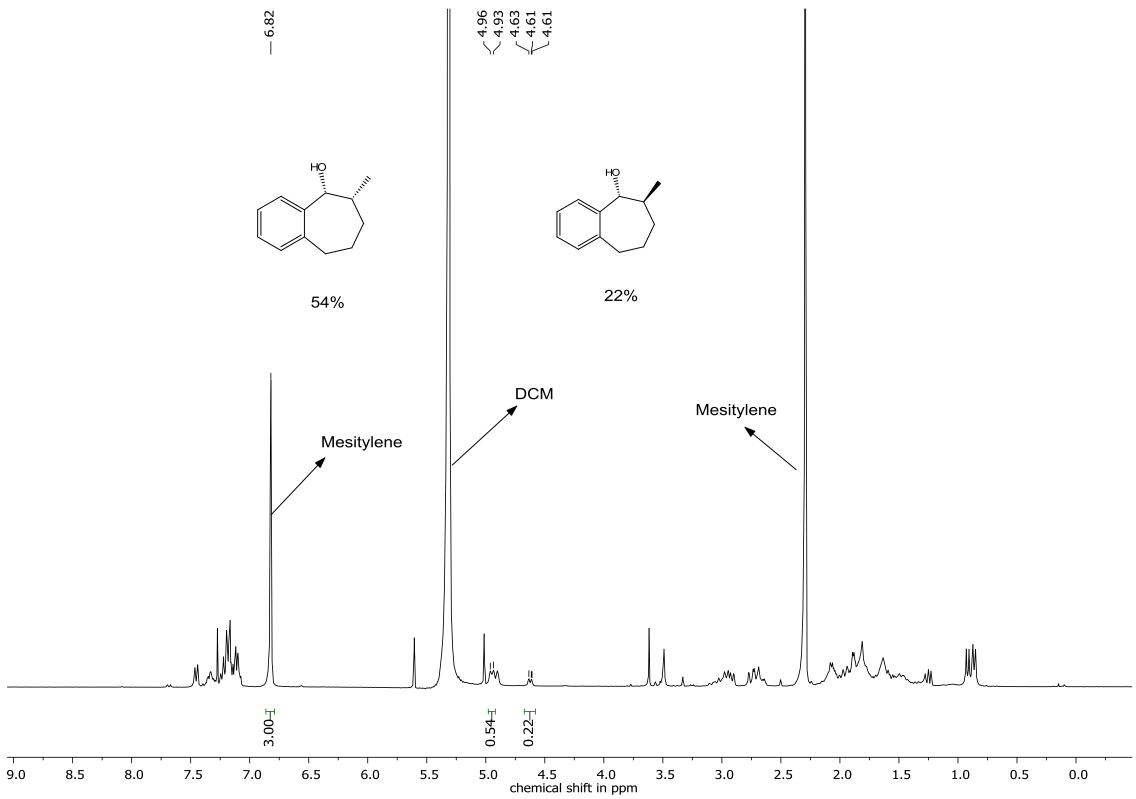

Figure S20: ^{1}H NMR (300 MHz, $CDCl_3$, 298 K) spectrum for 9k

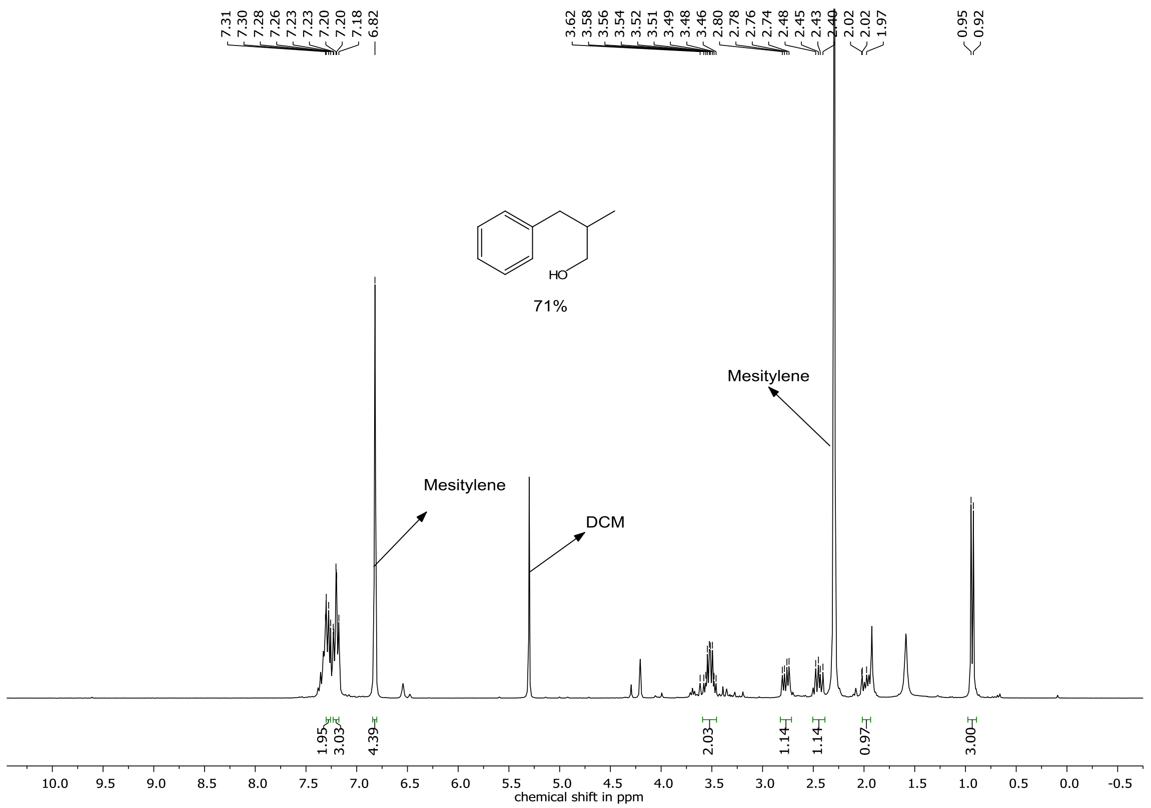

Figure S21: ^{1}H NMR (300 MHz, $CDCl_3$, 298 K) spectrum for 11a

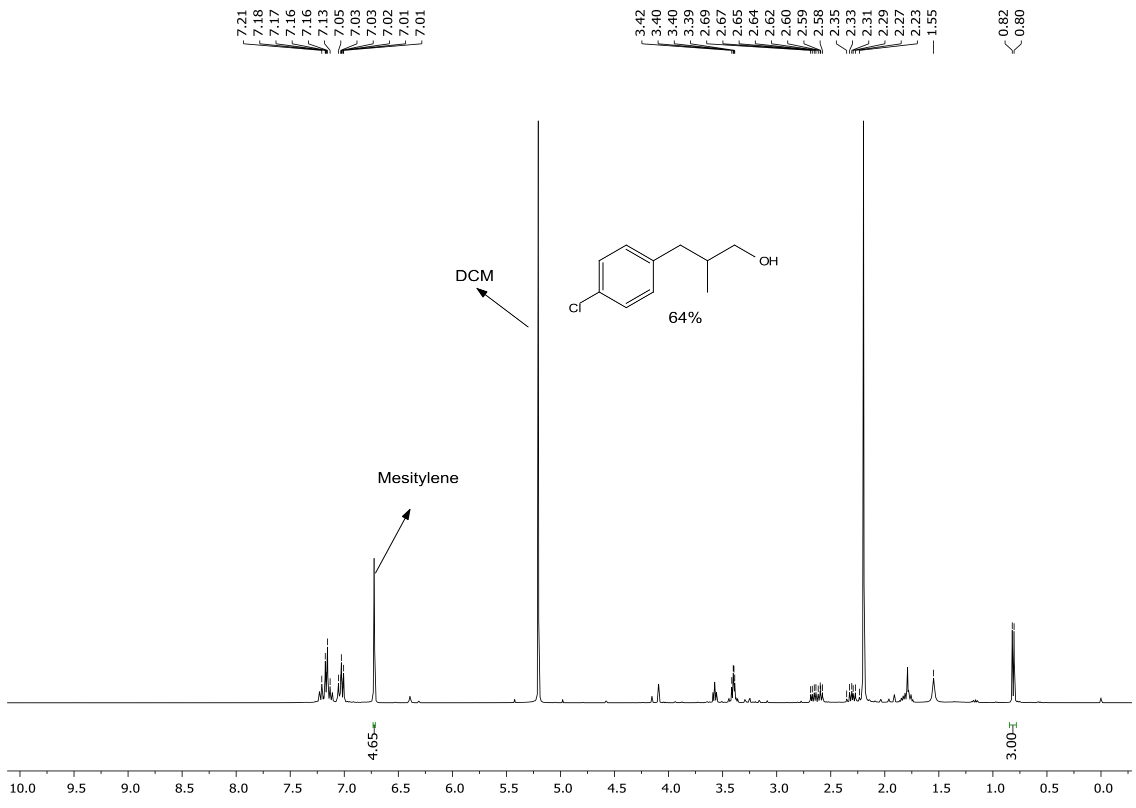

Figure S22: ^{1}H NMR (300 MHz, $CDCl_3$, 298 K) spectrum for 11b

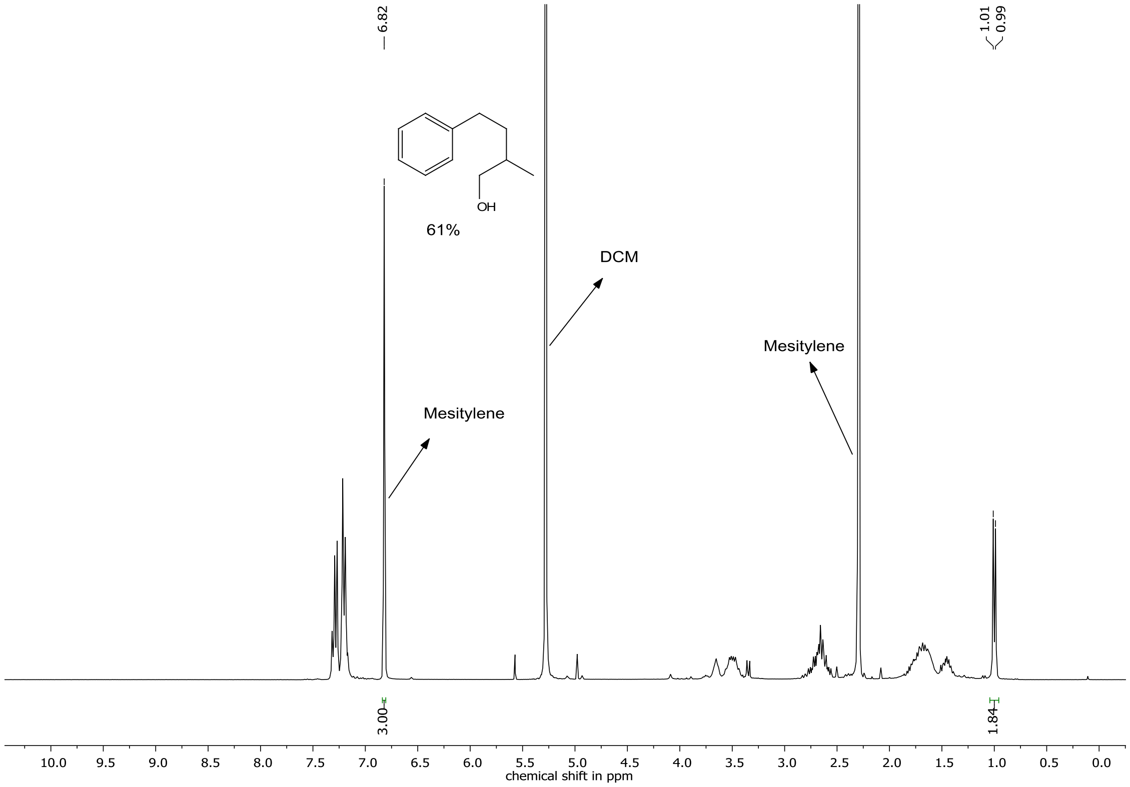

Figure S23: ^{1}H NMR (300 MHz, $CDCl_3$, 298 K) spectrum for 11c

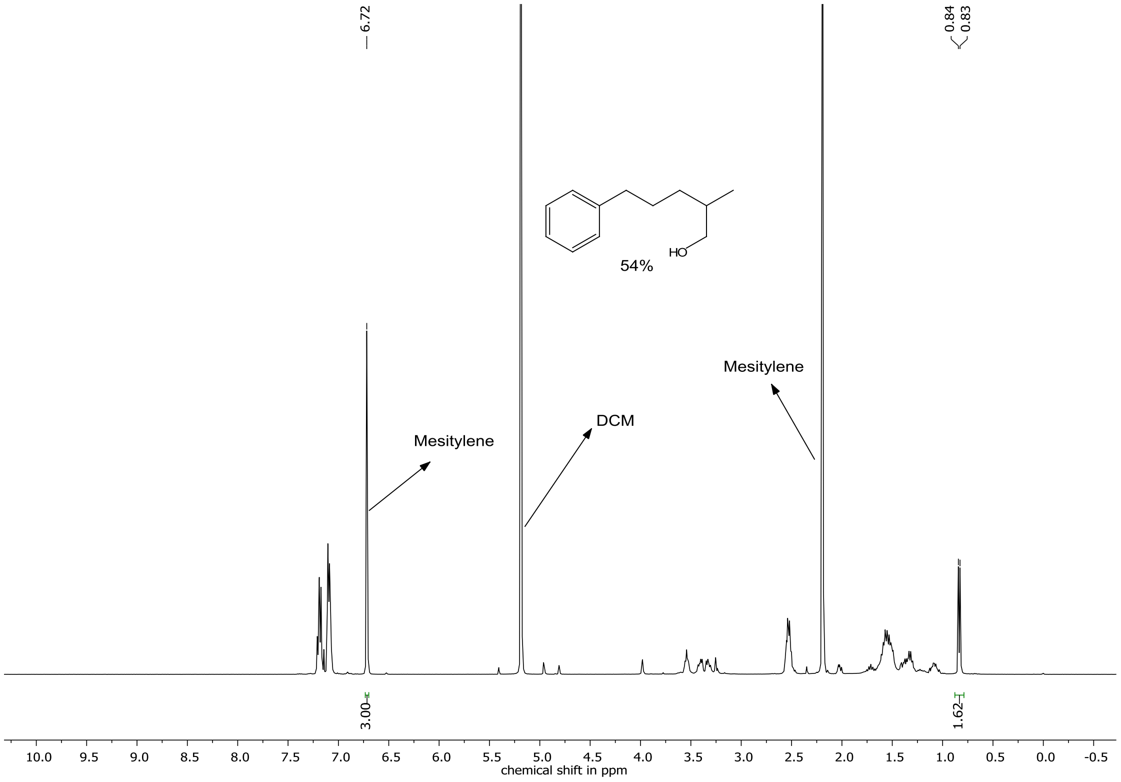

Figure S24: ^{1}H NMR (400 MHz, $CDCl_3$, 298 K) spectrum for 11d

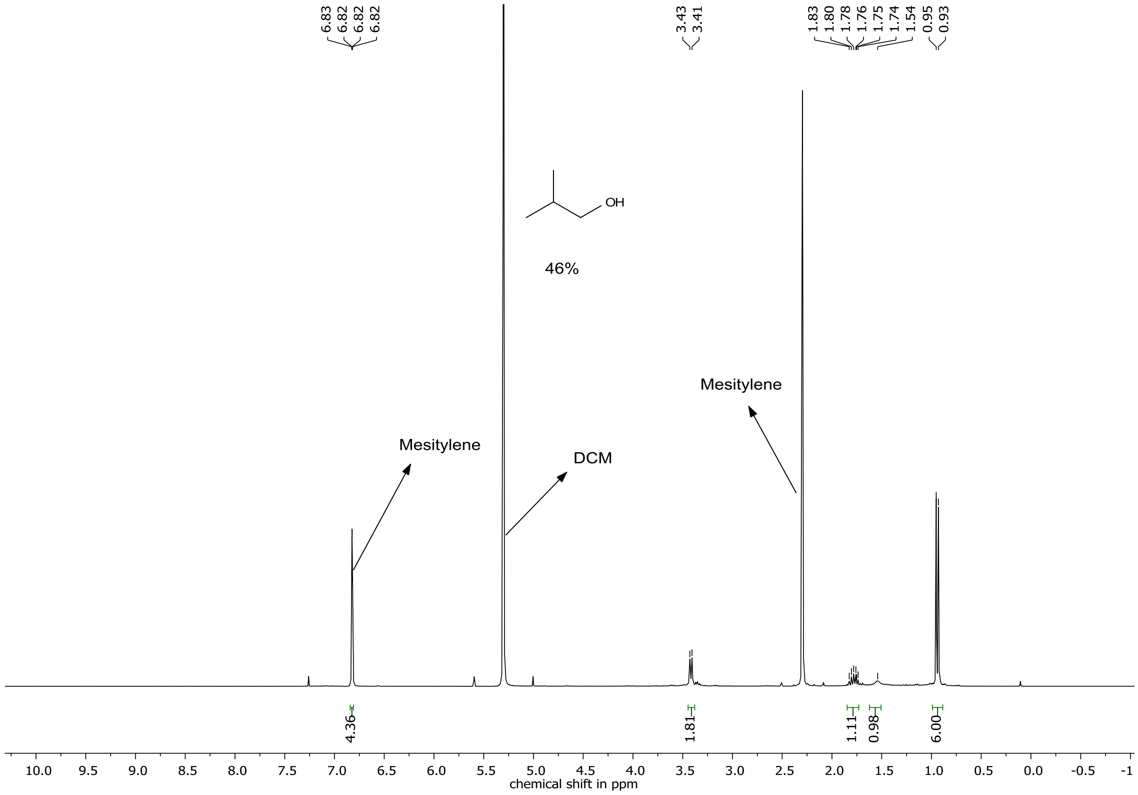

Figure S25: ^{1}H NMR (300 MHz, $CDCl_3$, 298 K) spectrum for 11e

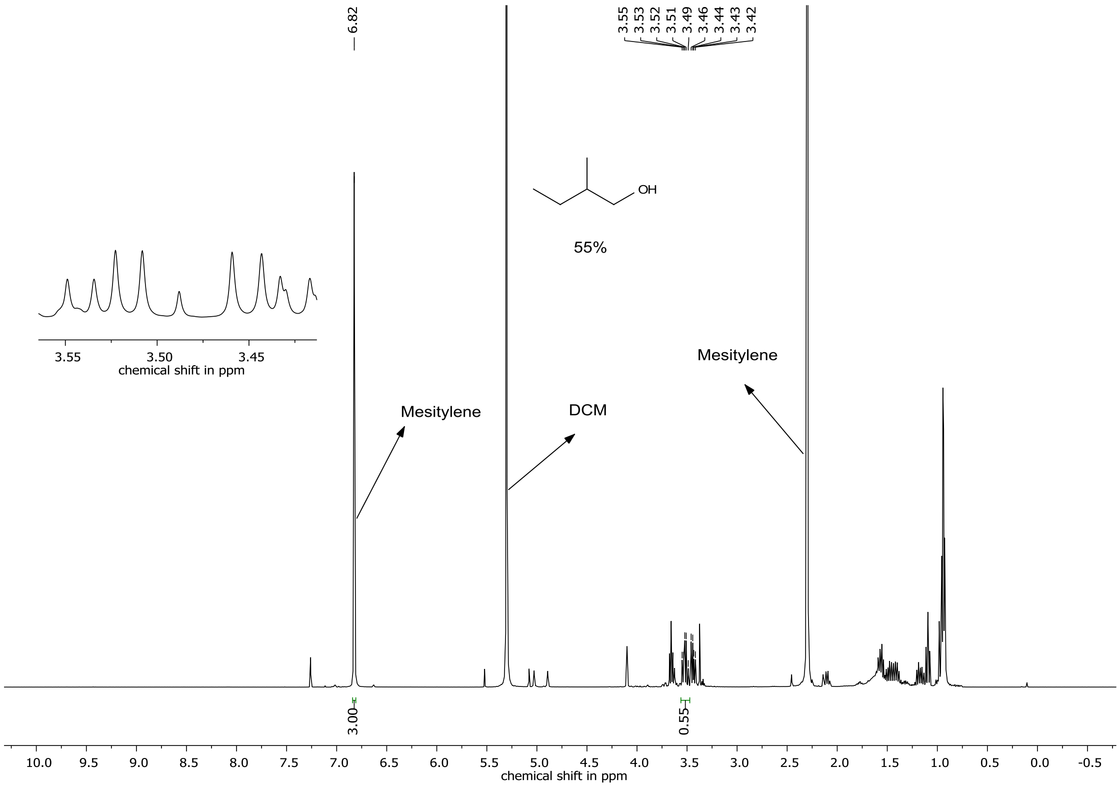

Figure S26: ^{1}H NMR (400 MHz, $CDCl_3$, 298 K) spectrum for 11f

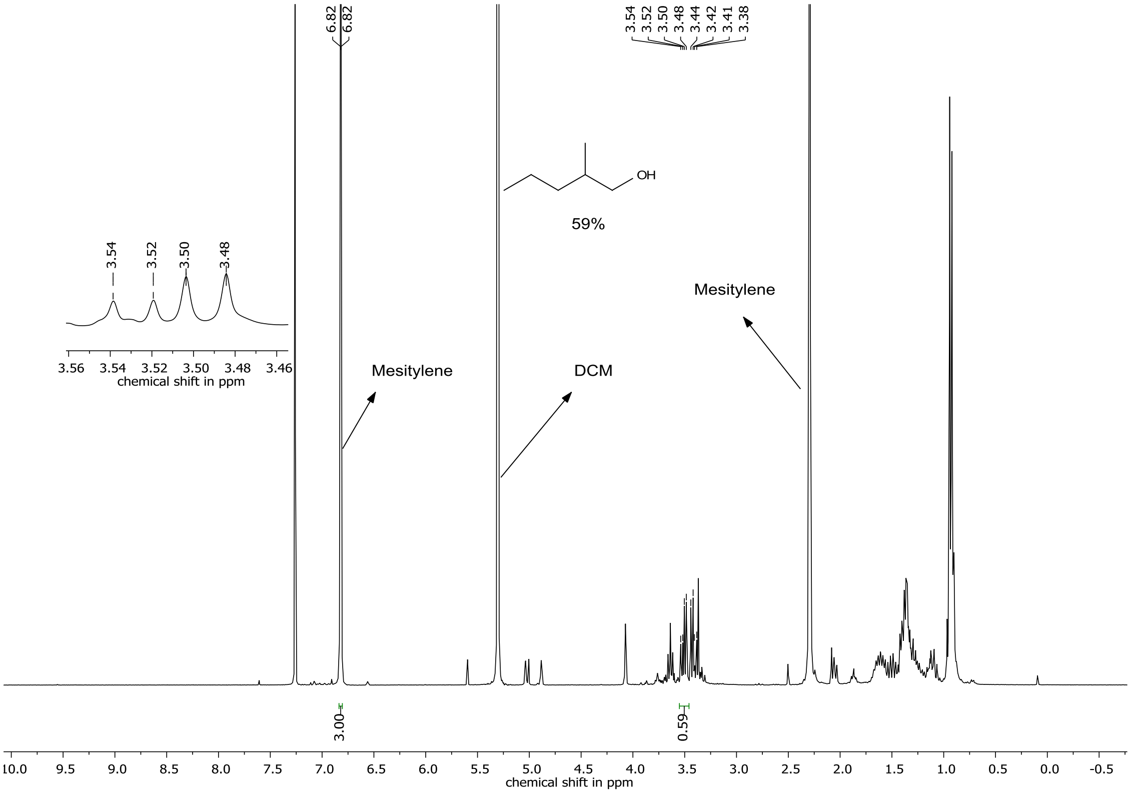

Figure S27: ^{1}H NMR (300 MHz, $CDCl_3$, 298 K) spectrum for 11g

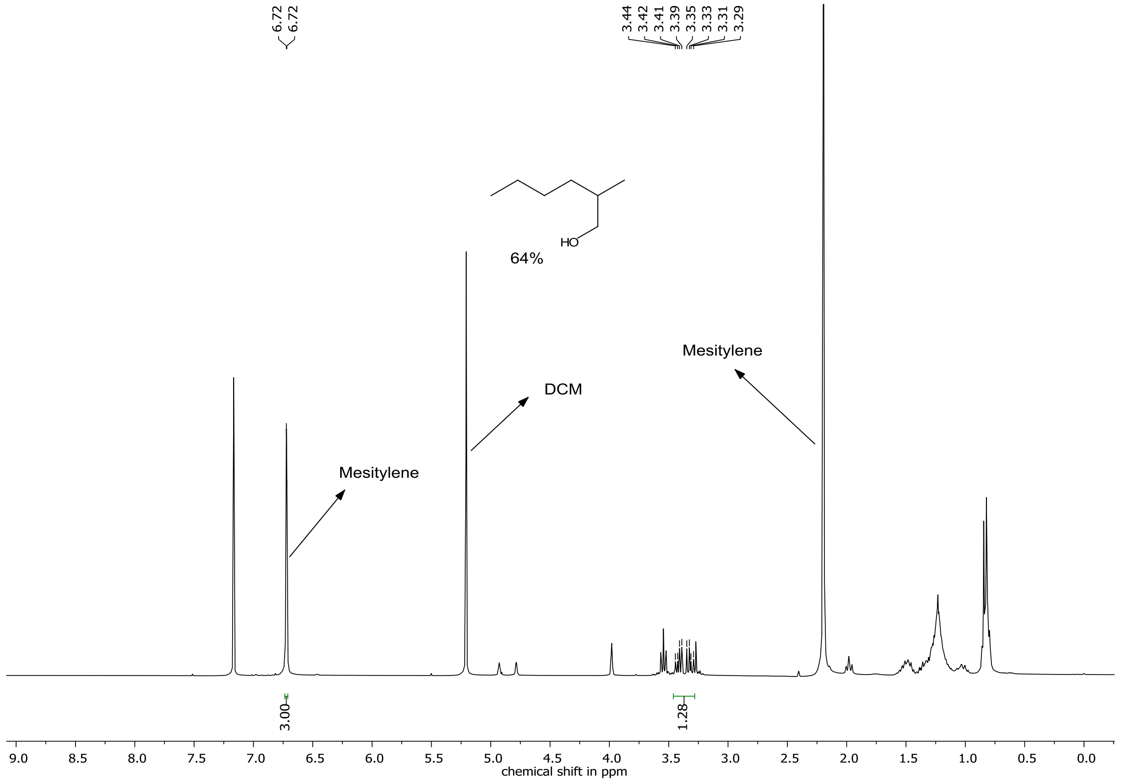

Figure S28: ^{1}H NMR (300 MHz, $CDCl_3$, 298 K) spectrum for 11h

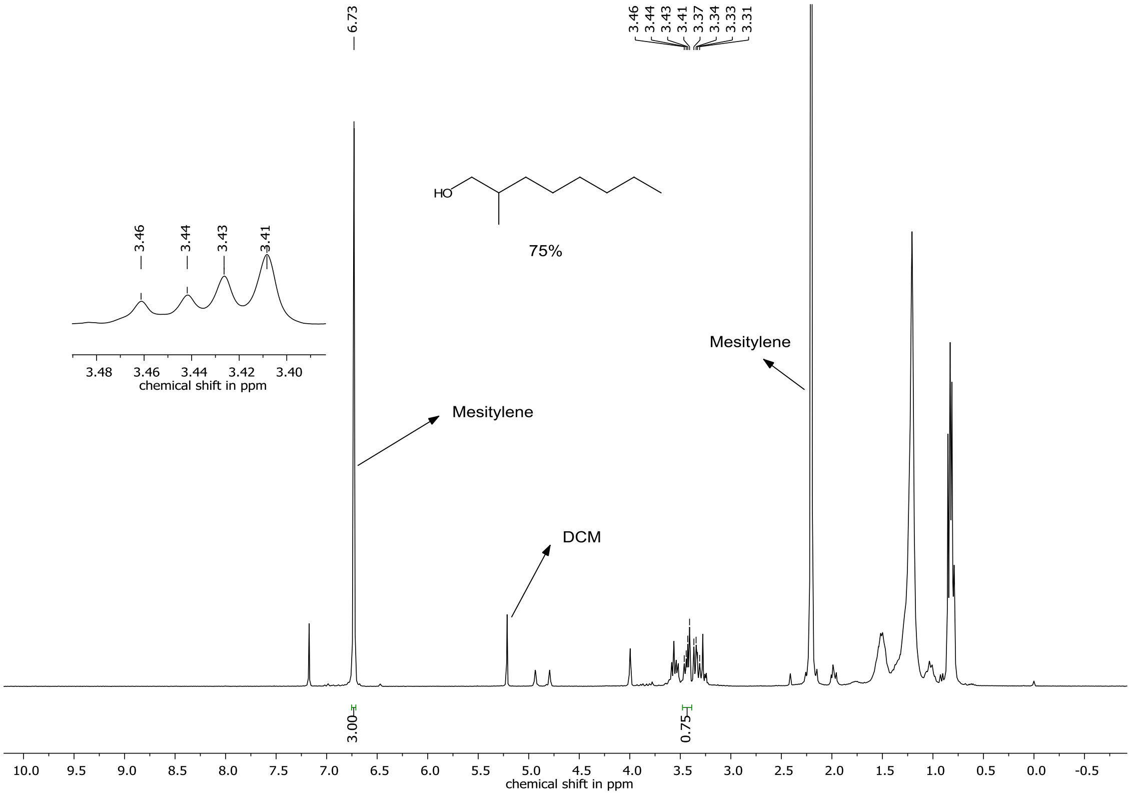

Figure S29: ^{1}H NMR (300 MHz, $CDCl_3$, 298 K) spectrum for 11i

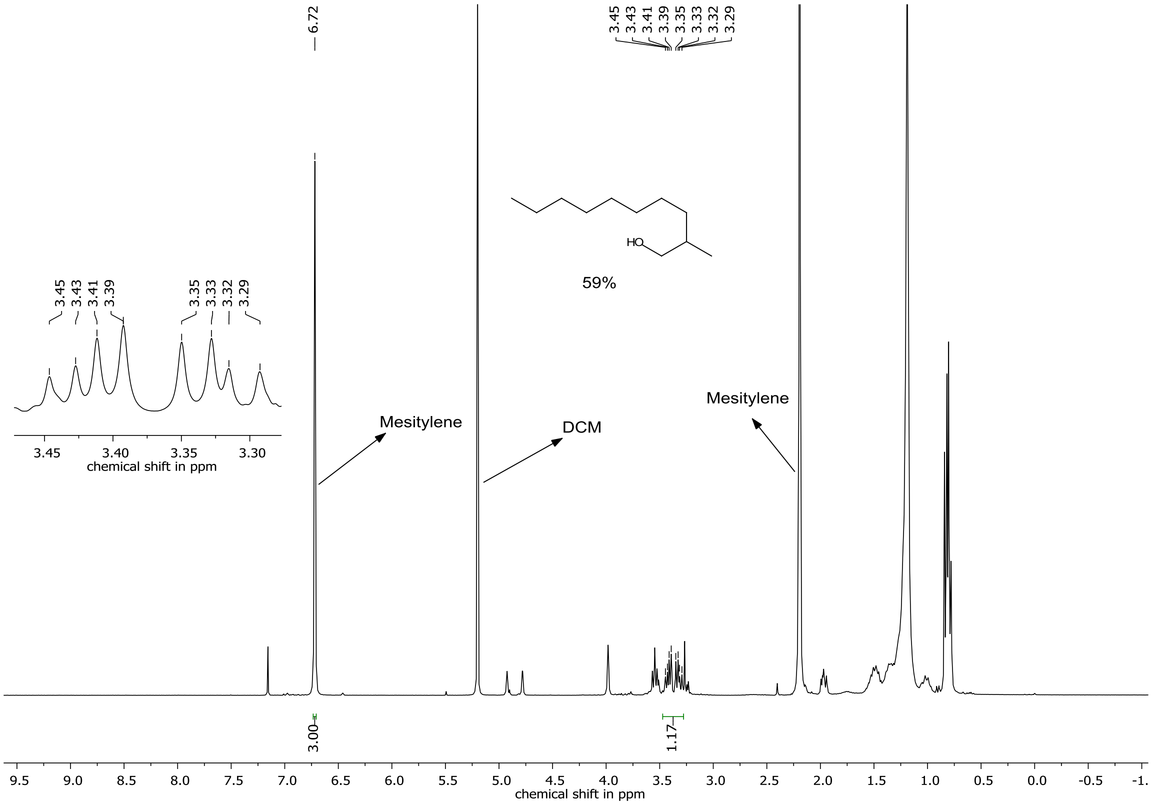

Figure S30: ^{1}H NMR (300 MHz, $CDCl_3$, 298 K) spectrum for 11j

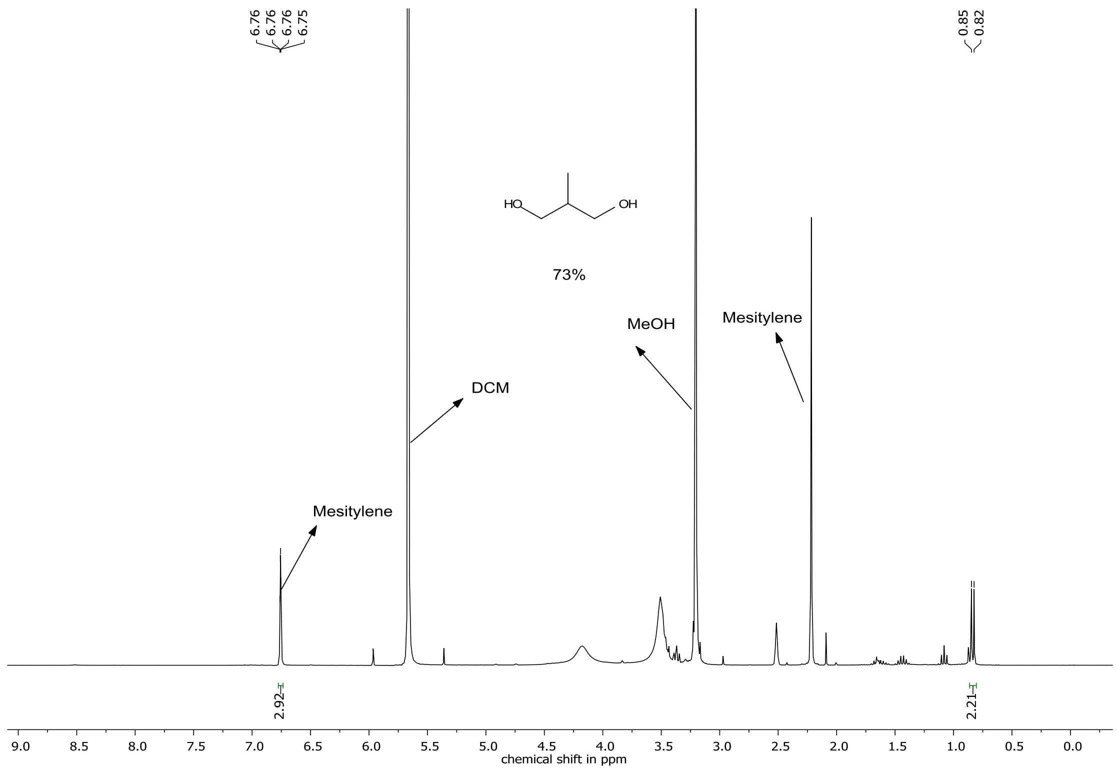

Figure S31: ^{1}H NMR (300 MHz, DMSO-d_6, 298 K) spectrum for 11k

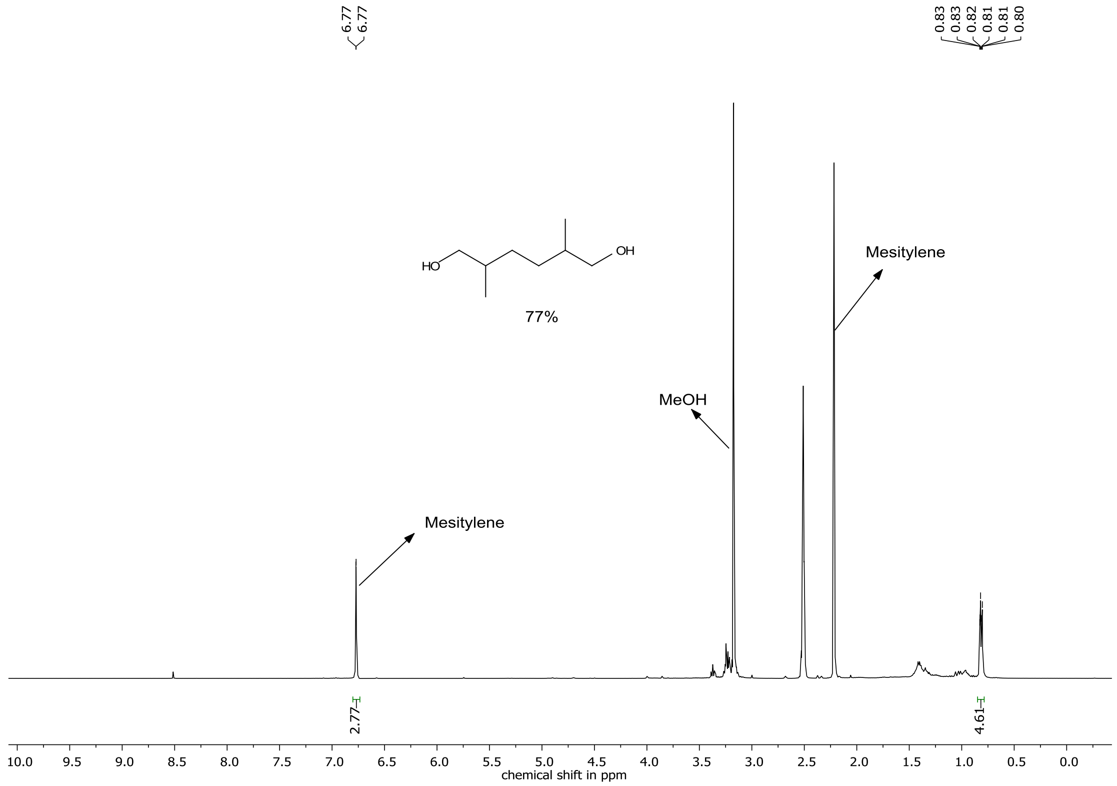

Figure S32: ^{1}H NMR (400 MHz, DMSO-d_6, 298 K) spectrum for 11l

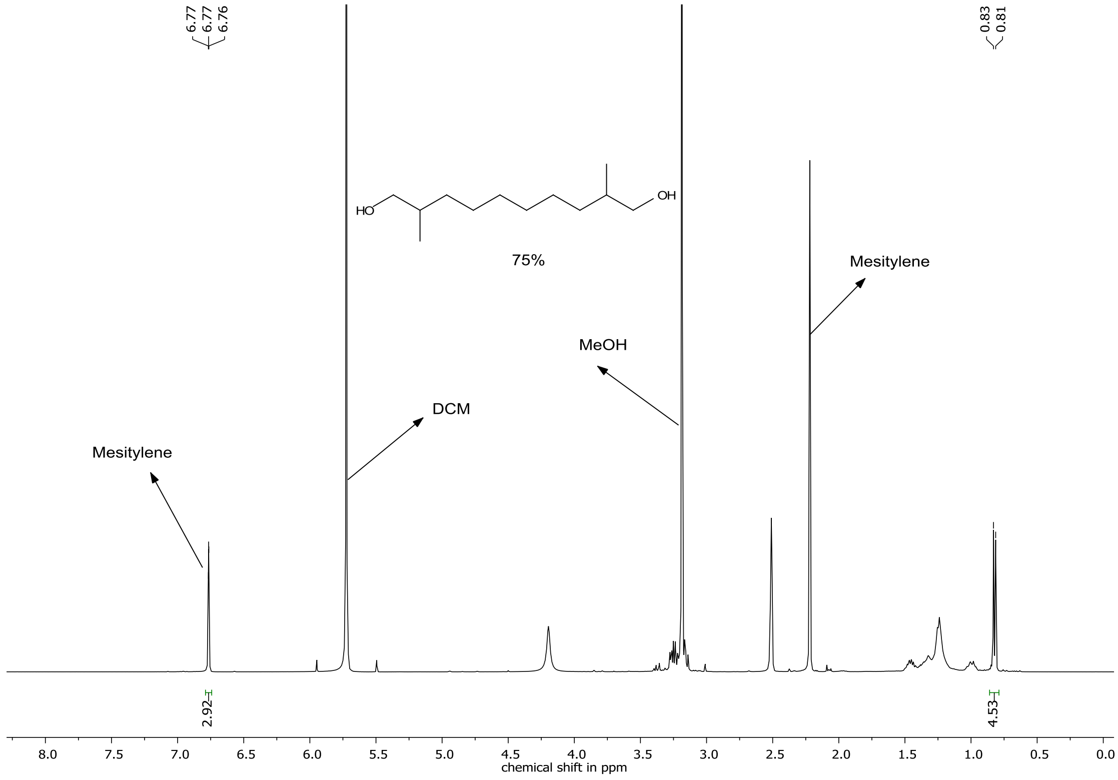

Figure S33: ^{1}H NMR (400 MHz, DMSO-d_6, 298 K) spectrum for 11m

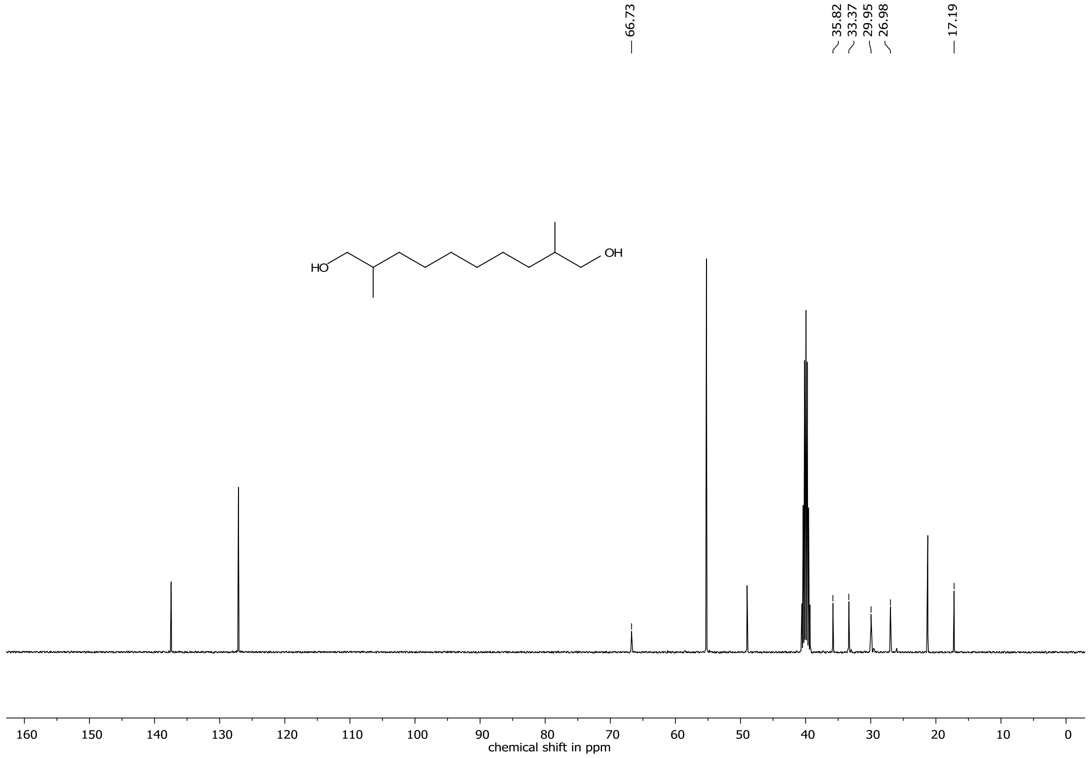

Figure S34: ^{13}C{^{1}H} NMR (101 MHz, DMSO-d_6, 298 K) spectrum for 11m

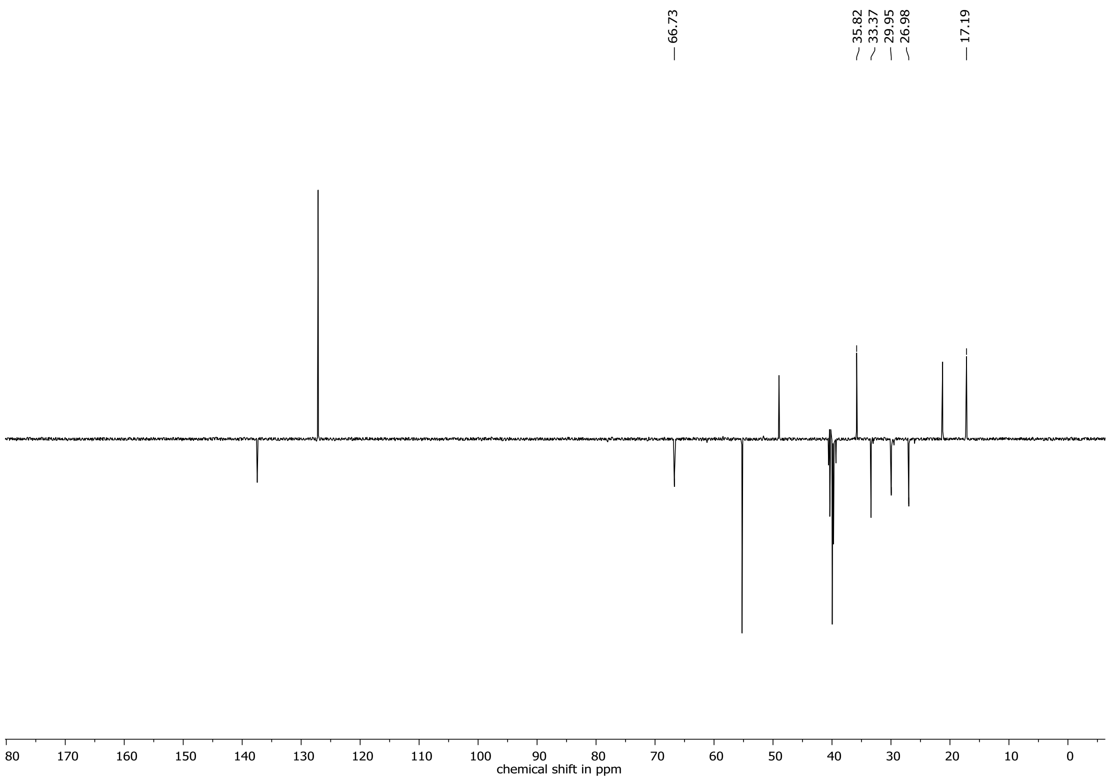

Figure S35: ^{13}C APT-NMR (101 MHz, DMSO-d_6, 298 K) spectrum for 11m

9. NMR spectra of isolated products

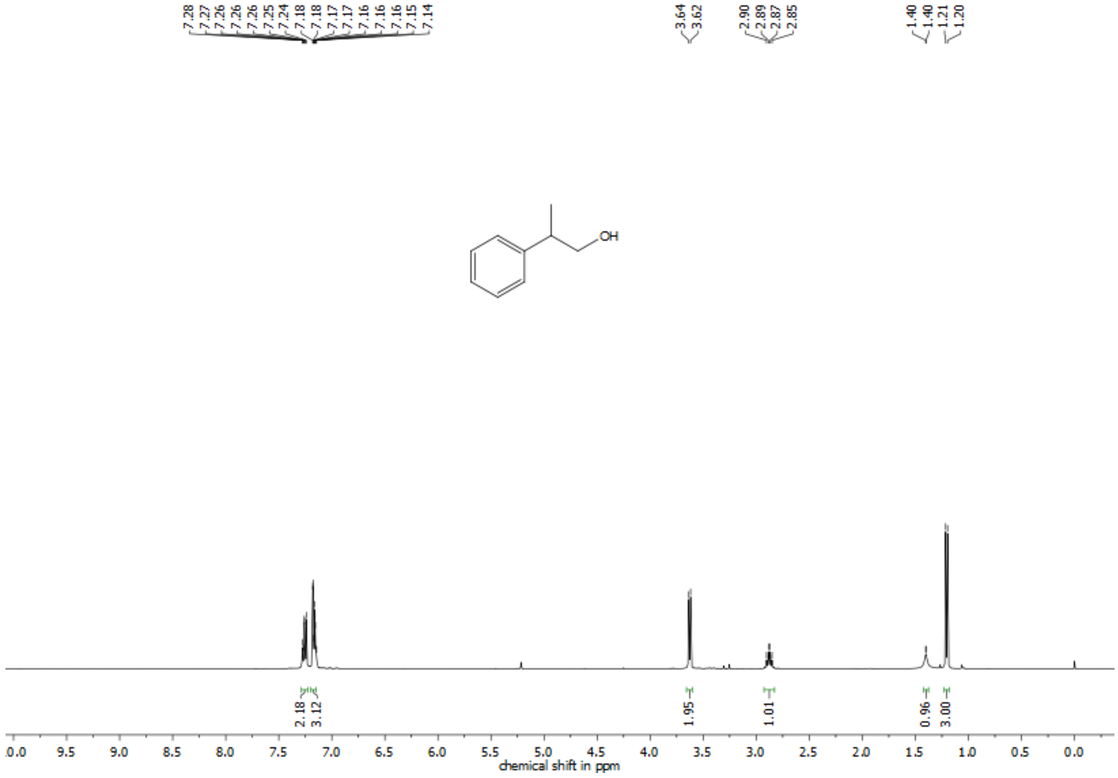

Figure S36: ^{1}H NMR (400 MHz, $CDCl_3$, 298 K) spectrum of 7a

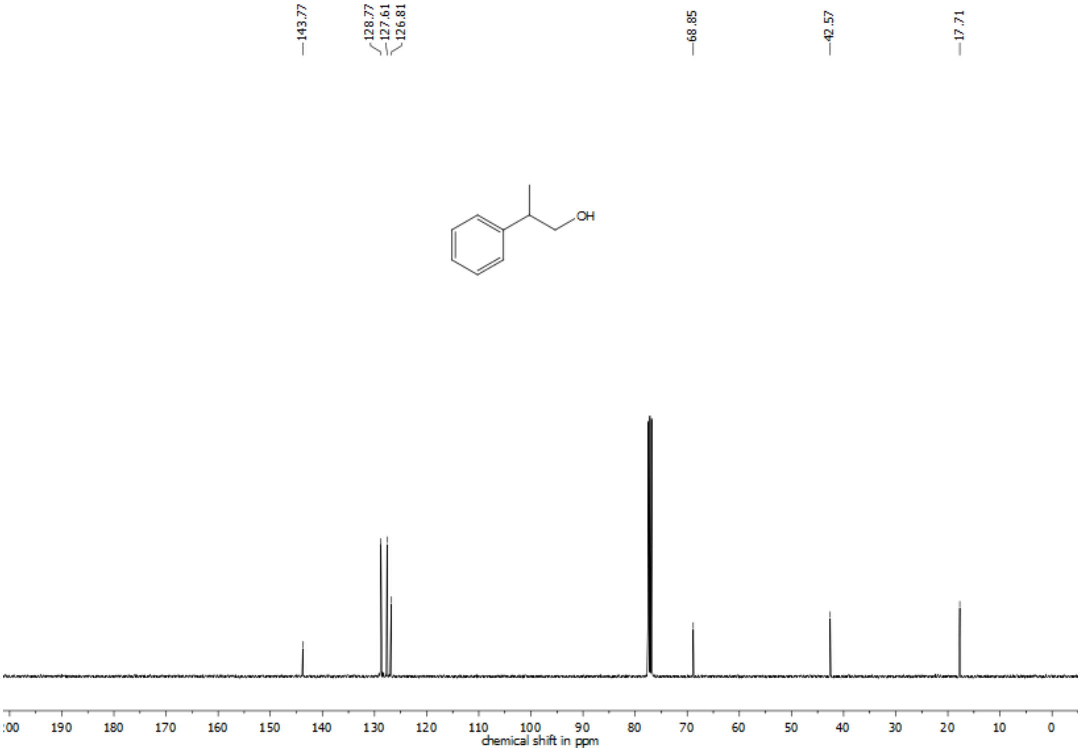

Figure S37: $^{13}C\{^{1}H\}$ NMR (101 MHz, $CDCl_3$, 298 K) spectrum of 7a

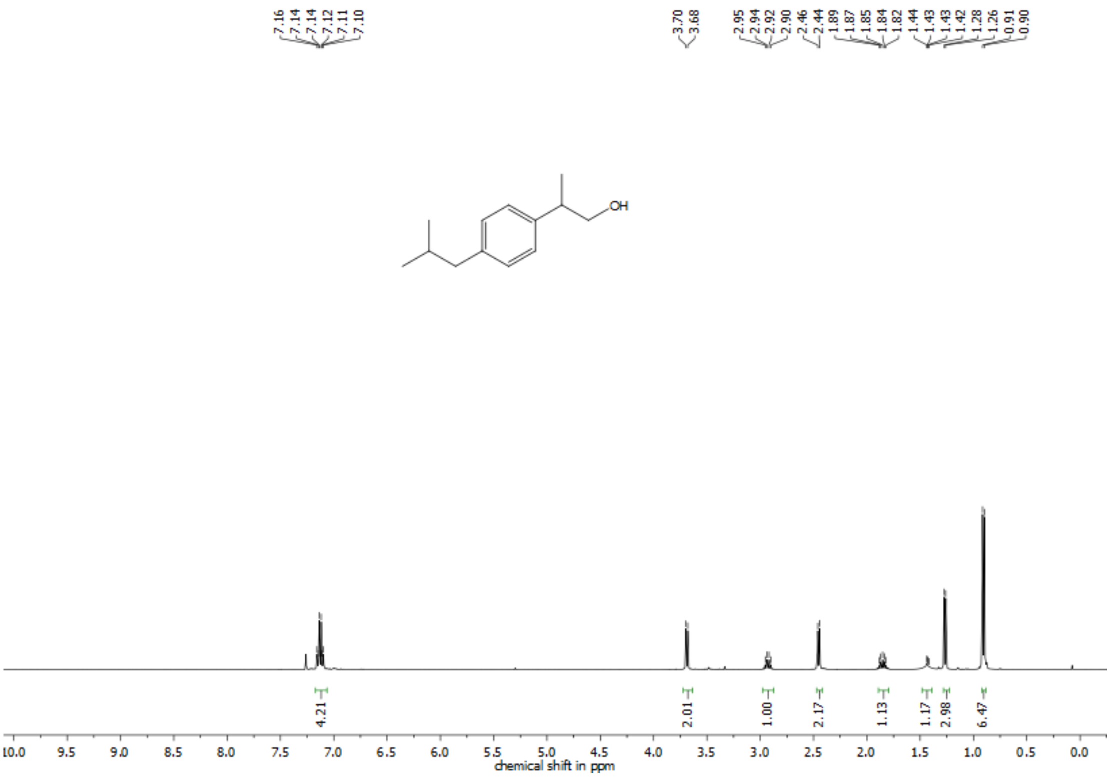

Figure S38: ^{1}H NMR (400 MHz, $CDCl_3$, 298 K) spectrum of 7c

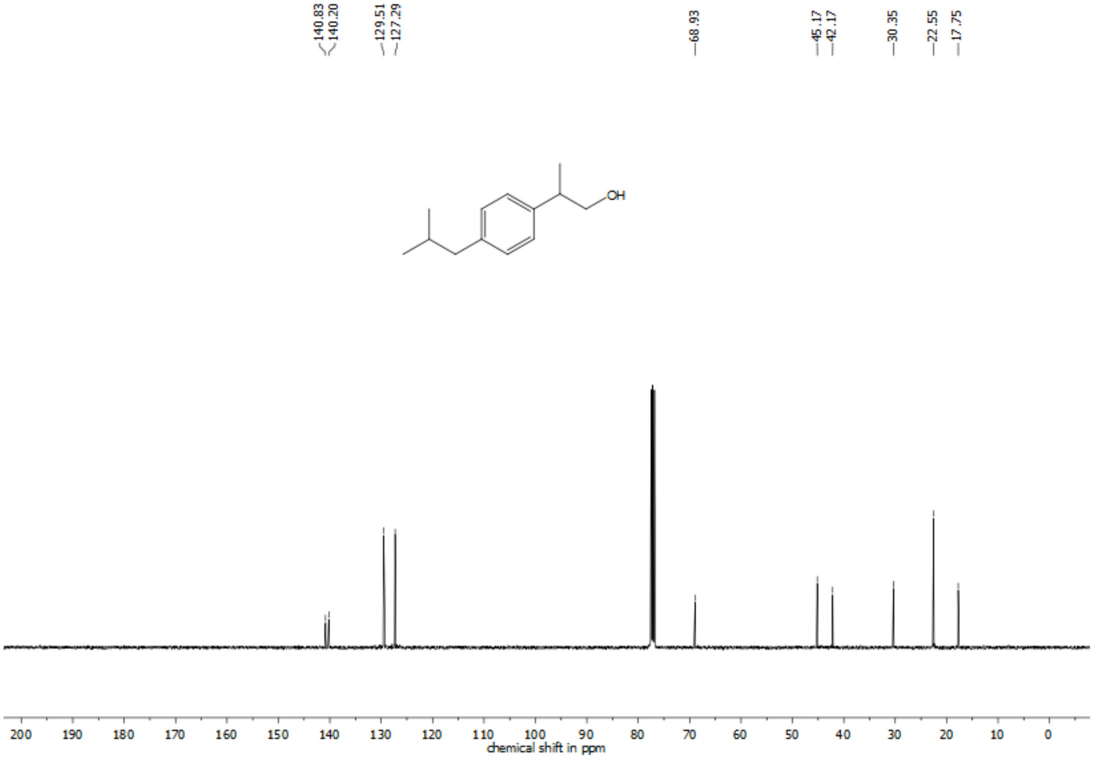

Figure S39: $^{13}C\{^{1}H\}$ NMR (101 MHz, $CDCl_3$, 298 K) spectrum of 7c

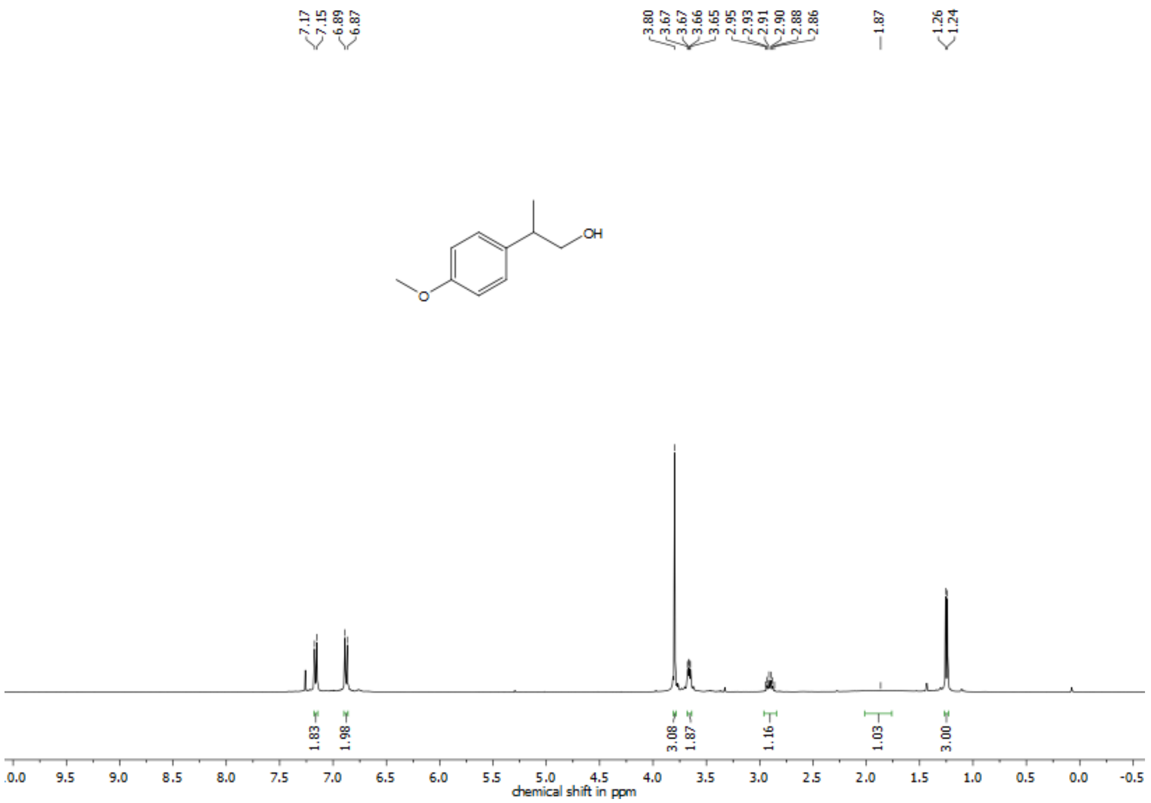

Figure S40: ^{1}H NMR (400 MHz, $CDCl_3$, 298 K) spectrum of 7d

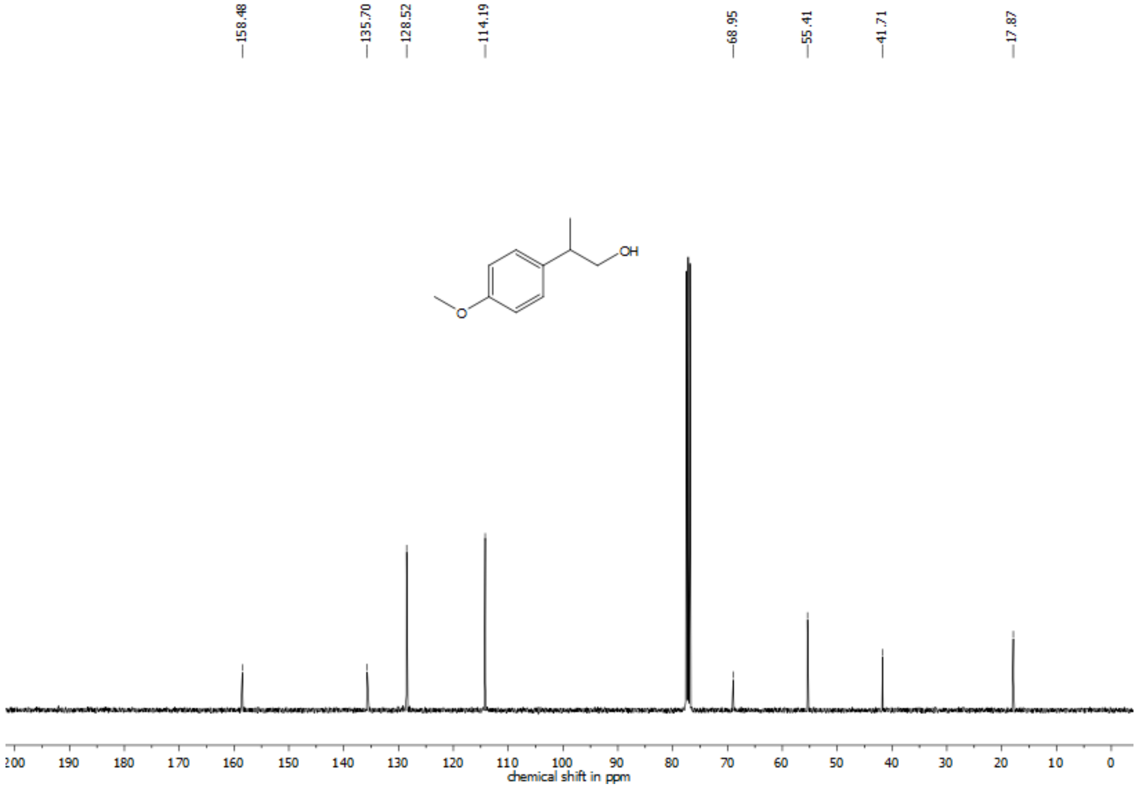

Figure S41: ^{13}C{^{1}H} NMR (101 MHz, $CDCl_3$, 298 K) spectrum of 7d

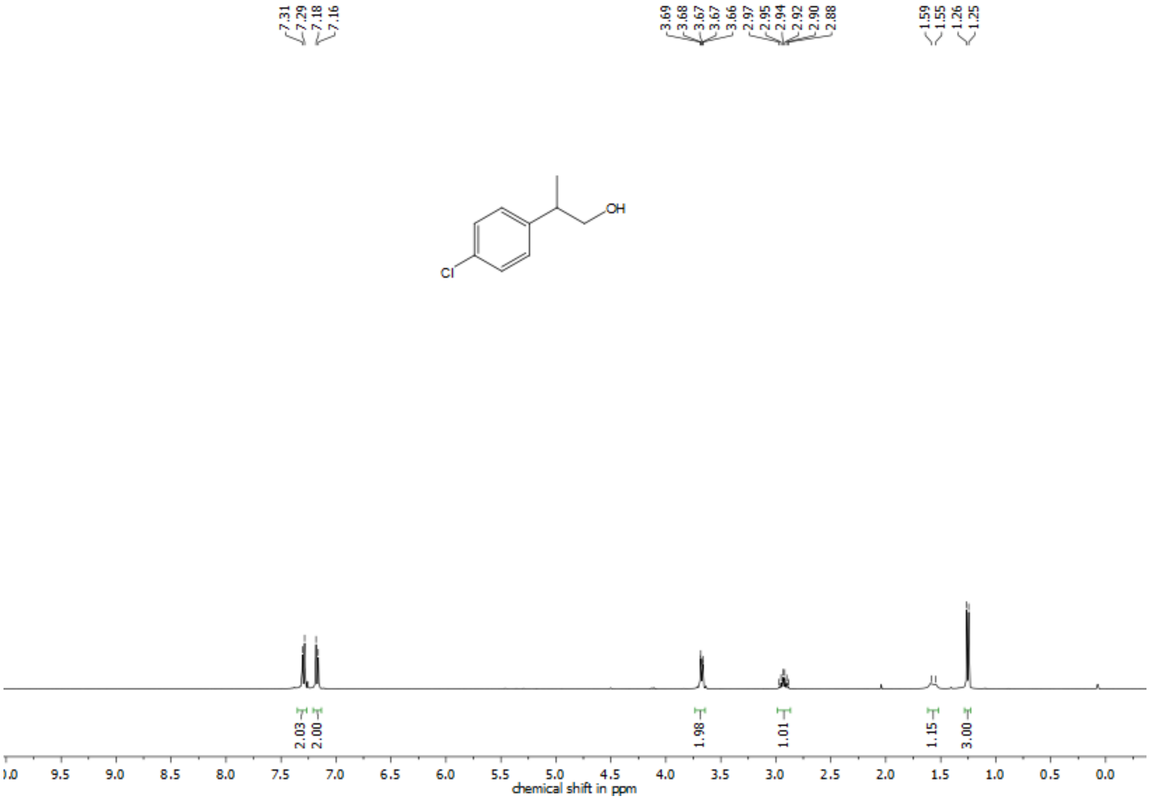

Figure S42: ^{1}H NMR (400 MHz, $CDCl_3$, 298 K) spectrum of 7g

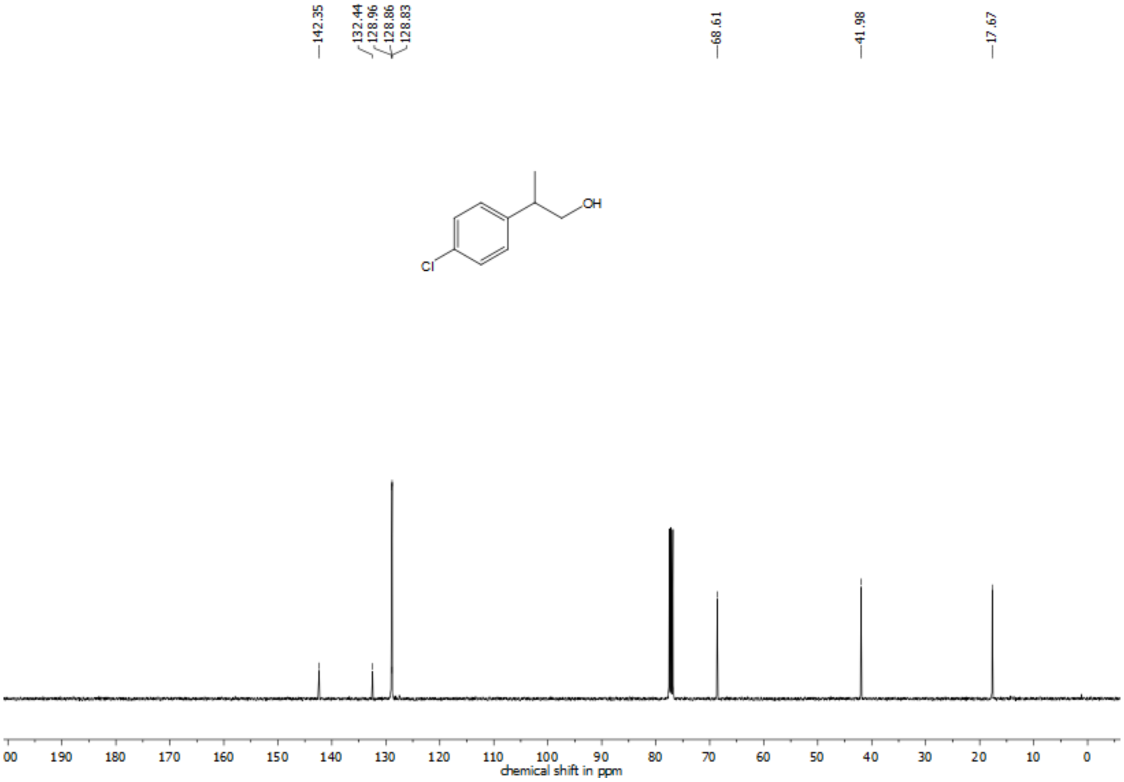

Figure S43: $^{13}C\{^{1}H\}$ NMR (101 MHz, $CDCl_3$, 298 K) spectrum of 7g

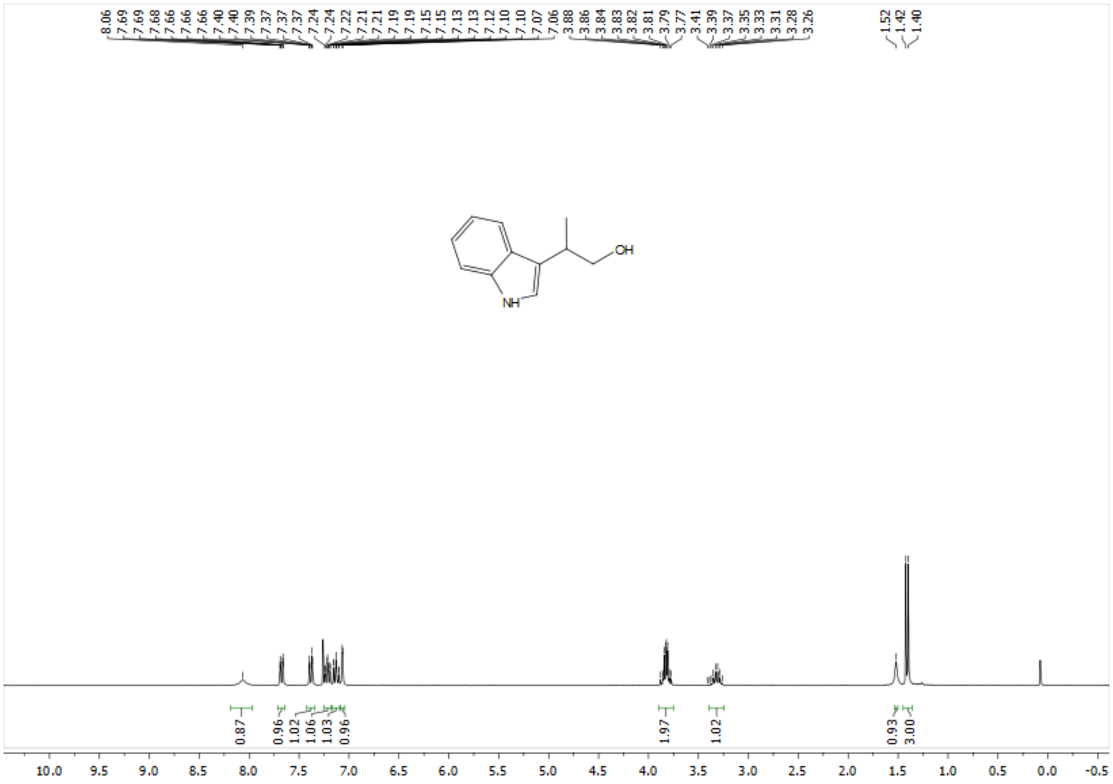

Figure S44: ^{1}H NMR (300 MHz, $CDCl_3$, 298 K) spectrum of 7j

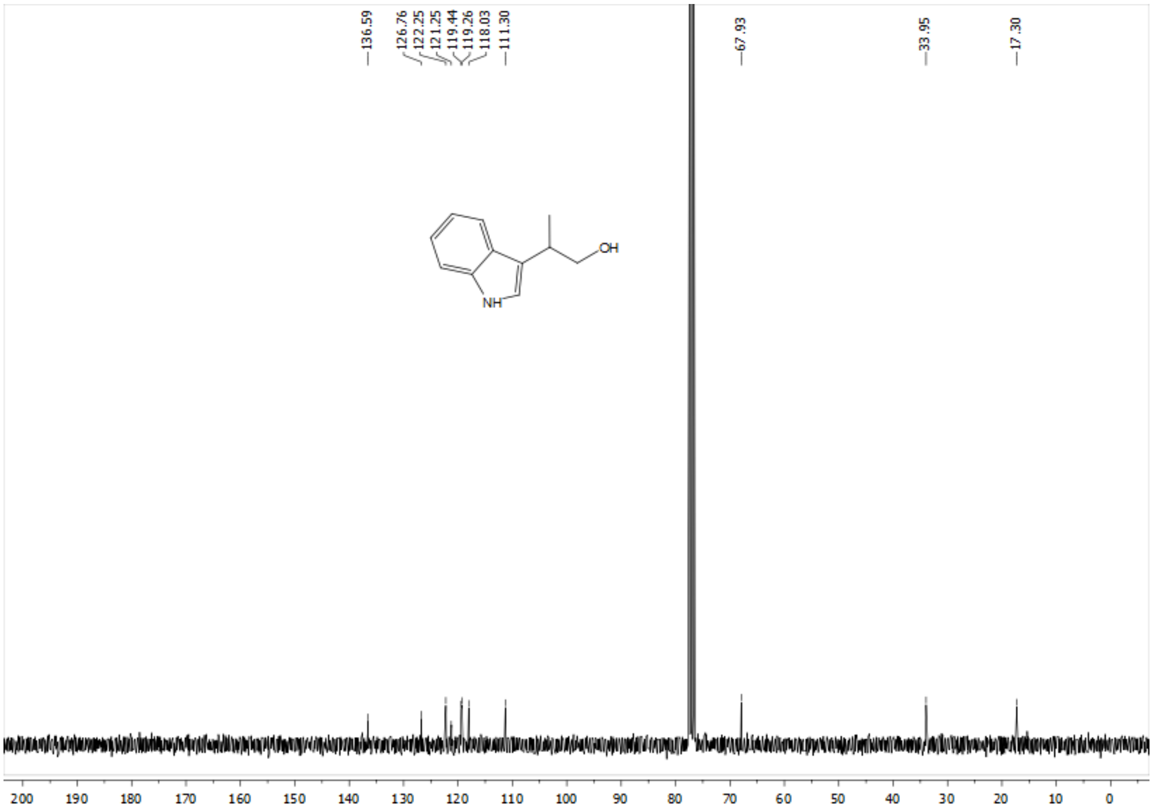

Figure S45: $^{13}C\{^{1}H\}$ NMR (75 MHz, $CDCl_3$, 298 K) spectrum of 7j

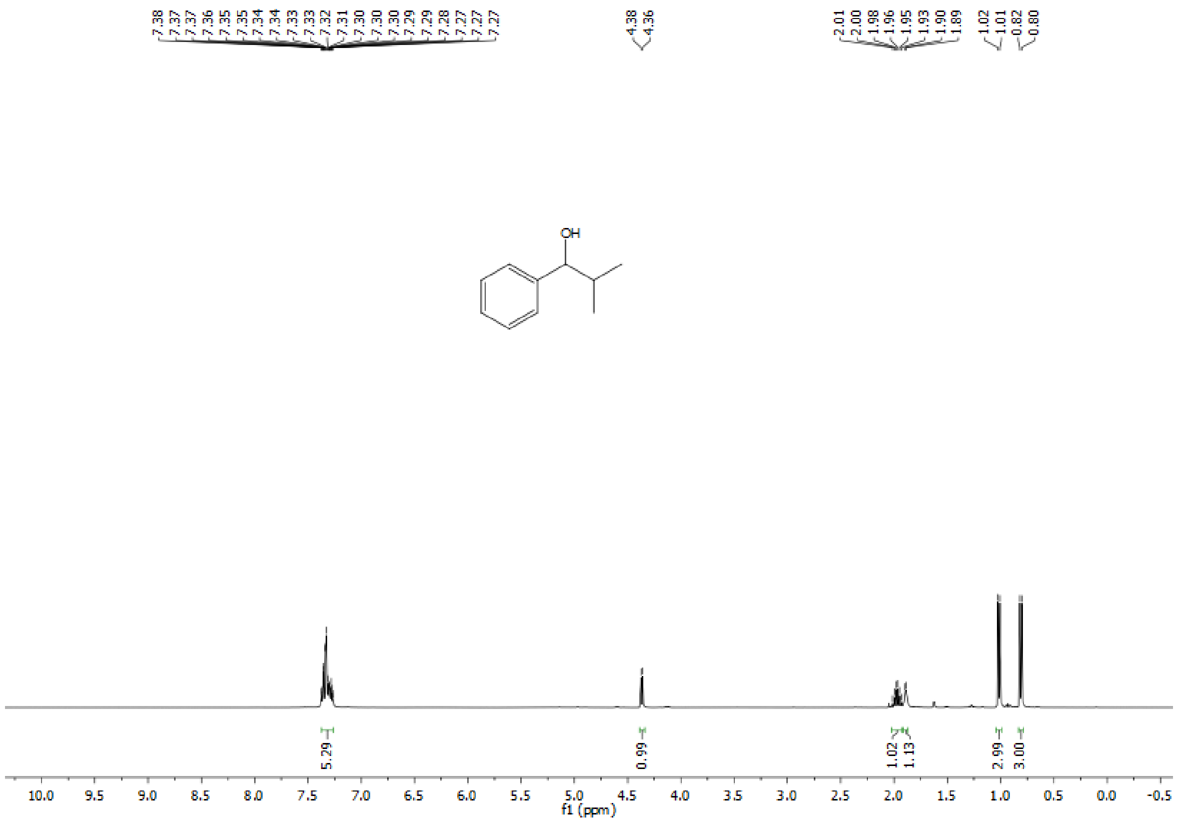

Figure S46: ^{1}H NMR (400 MHz, $CDCl_3$, 298 K) spectrum of 9a

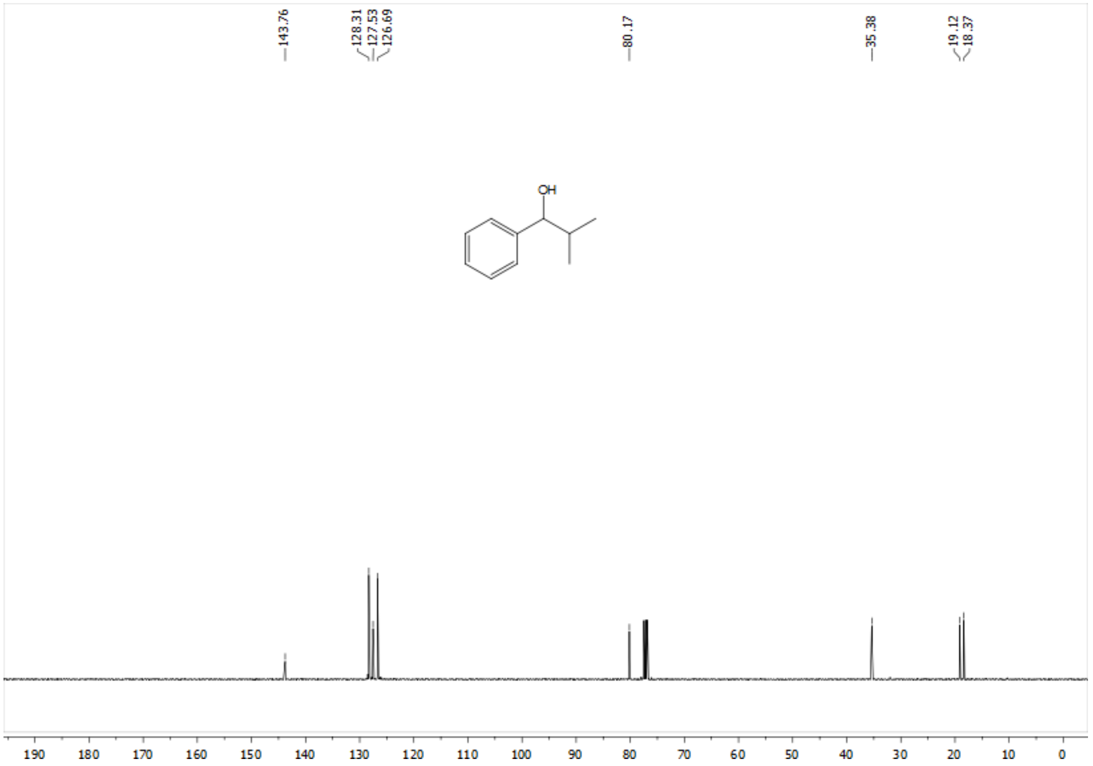

Figure S47: $^{13}C\{^{1}H\}$ NMR (101 MHz, $CDCl_3$, 298 K) spectrum of 9a

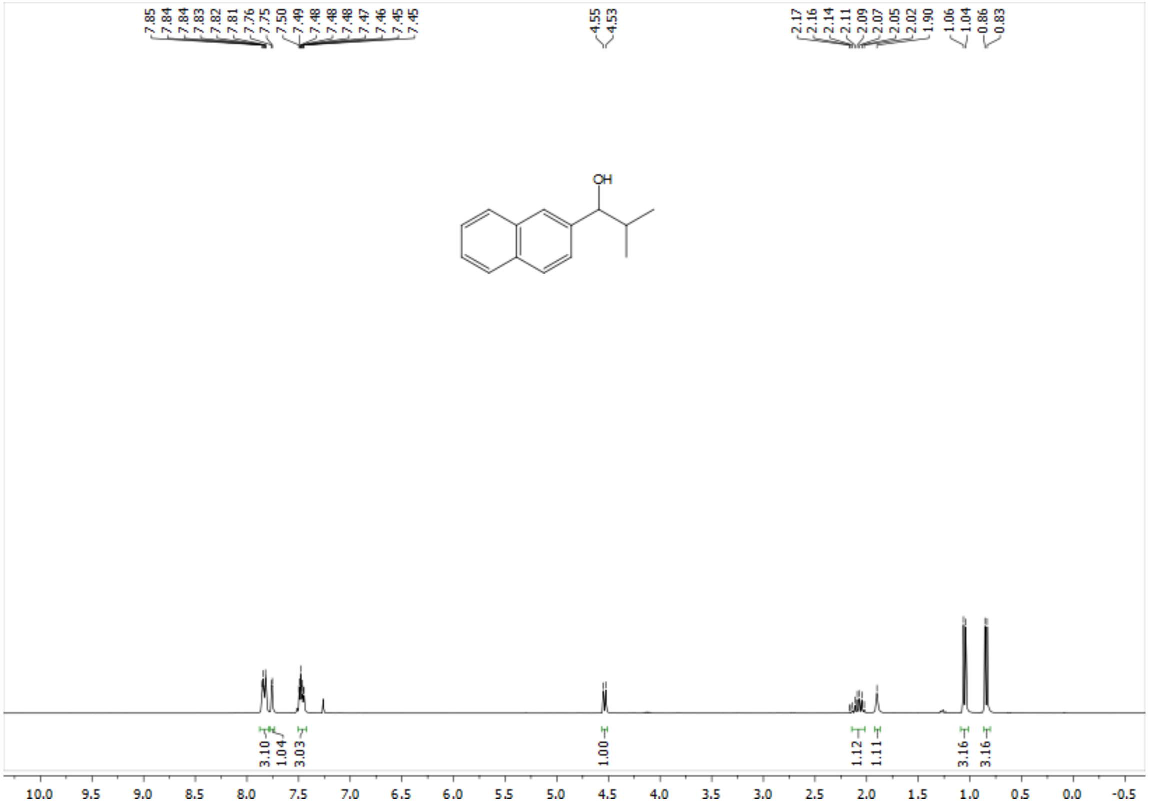

Figure S48: ^{1}H NMR (400 MHz, $CDCl_3$, 298 K) spectrum of 9b

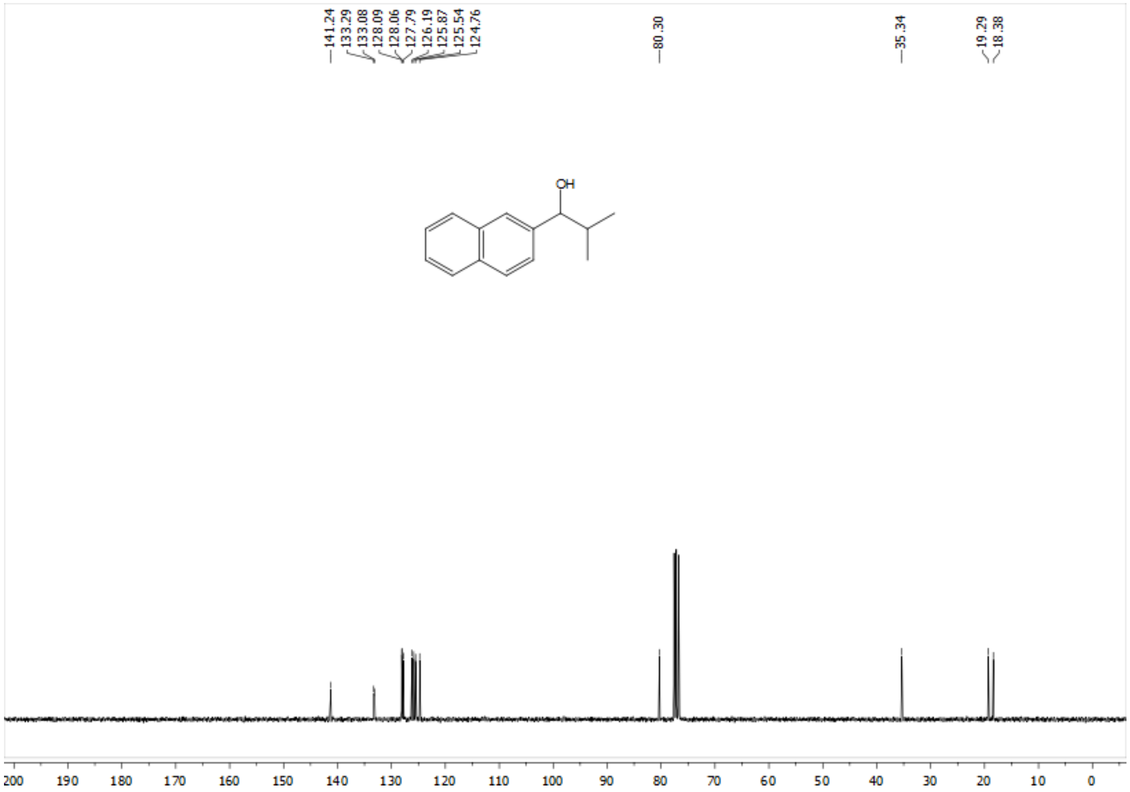

Figure S49: $^{13}C\{^{1}H\}$ NMR (101 MHz, $CDCl_3$, 298 K) spectrum of 9b

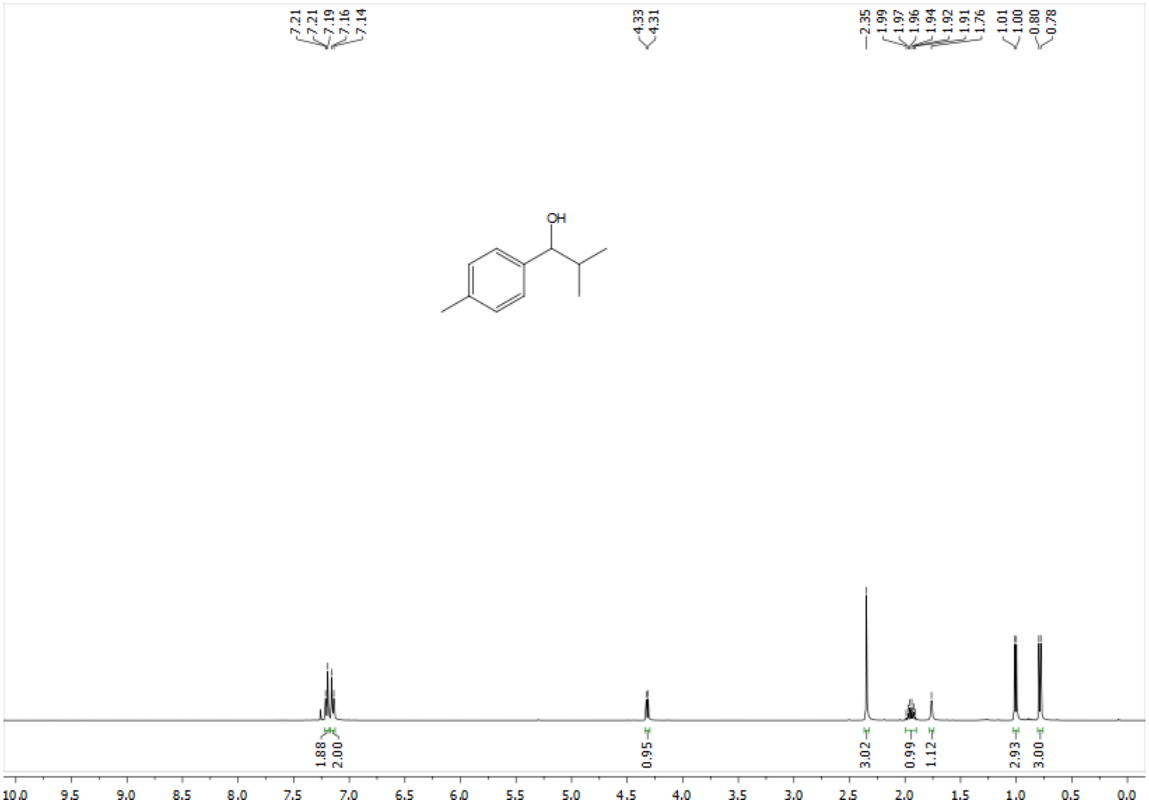

Figure S50: ^{1}H NMR (400 MHz, $CDCl_3$, 298 K) spectrum of 9c

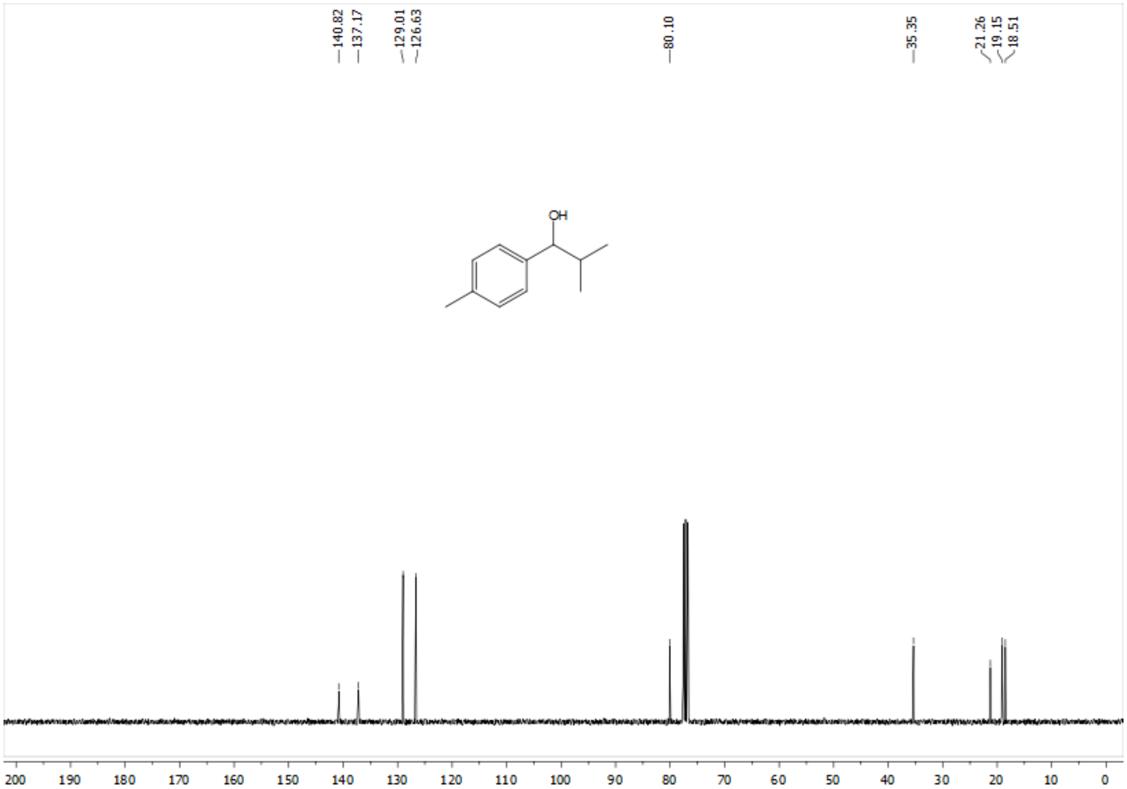

Figure S51: $^{13}C\{^{1}H\}$ NMR (101 MHz, $CDCl_3$, 298 K) spectrum of 9c

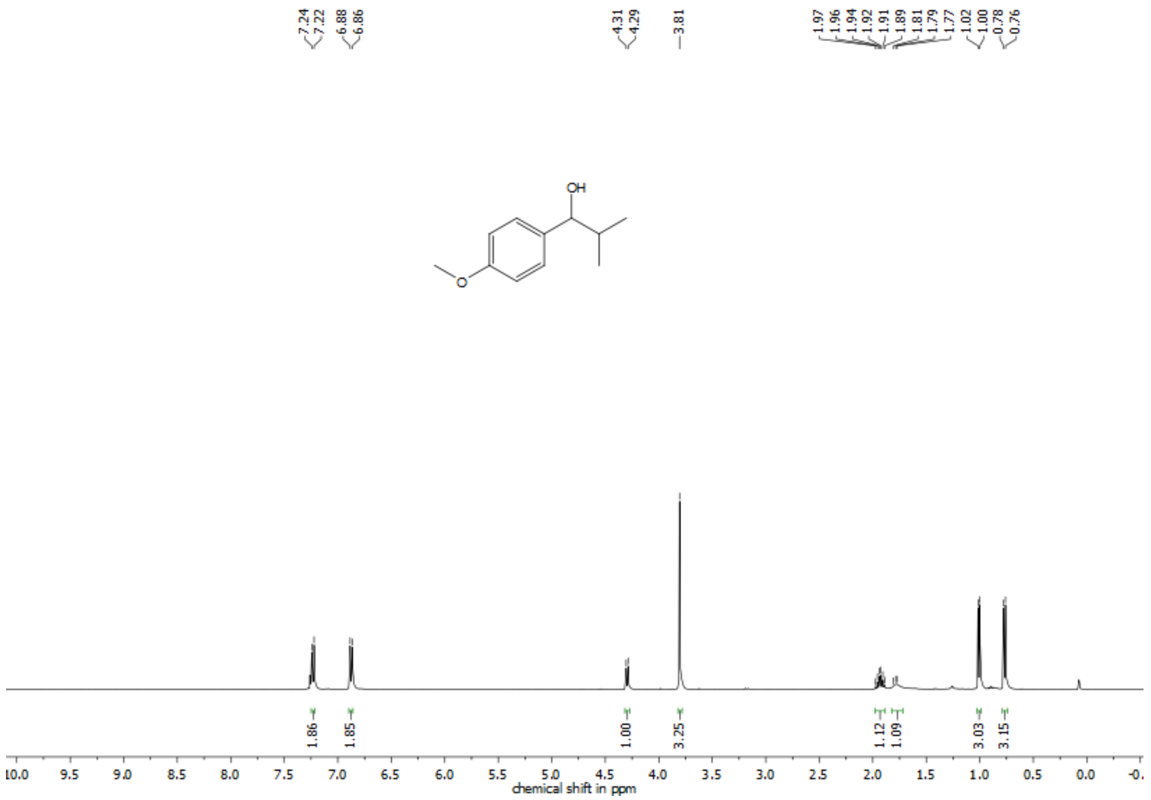

Figure S52: ^{1}H NMR (400 MHz, $CDCl_3$, 298 K) spectrum of 9e

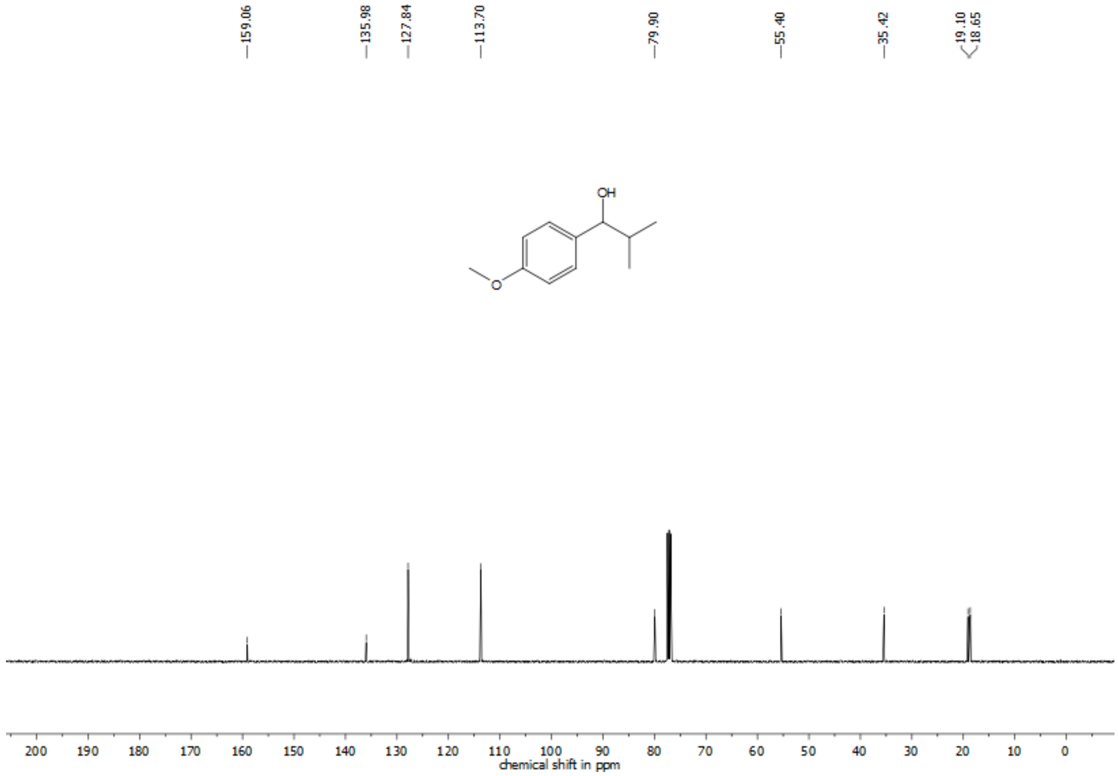

Figure S53: $^{13}C\{^{1}H\}$ NMR (101 MHz, $CDCl_3$, 298 K) spectrum of 9e

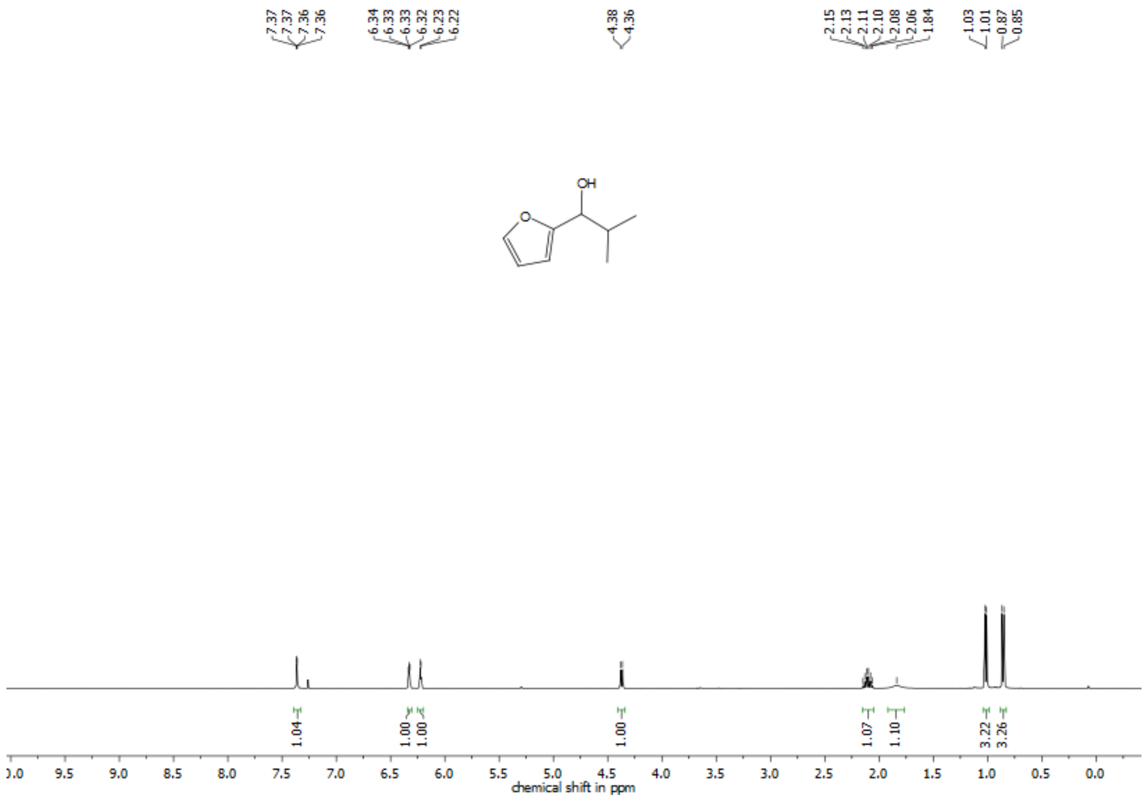

Figure S54: ^{1}H NMR (400 MHz, $CDCl_3$, 298 K) spectrum of 9g

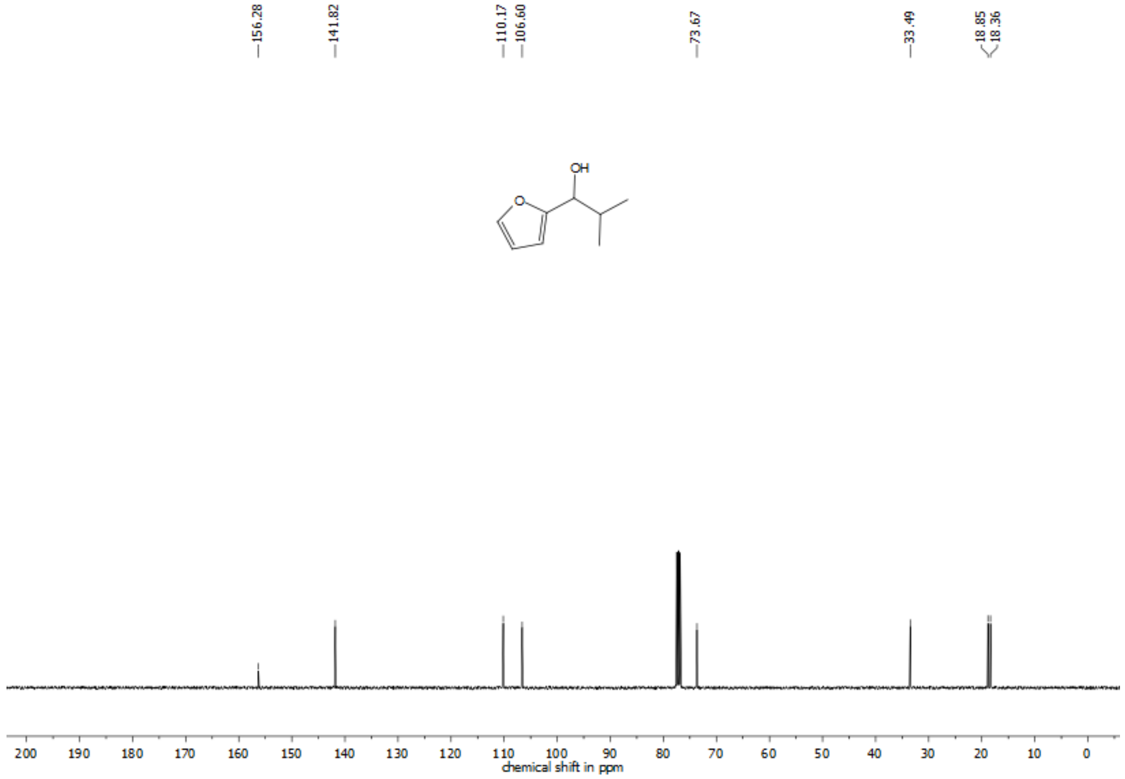

Figure S55: $^{13}C\{^{1}H\}$ NMR (101 MHz, $CDCl_3$, 298 K) spectrum of 9g

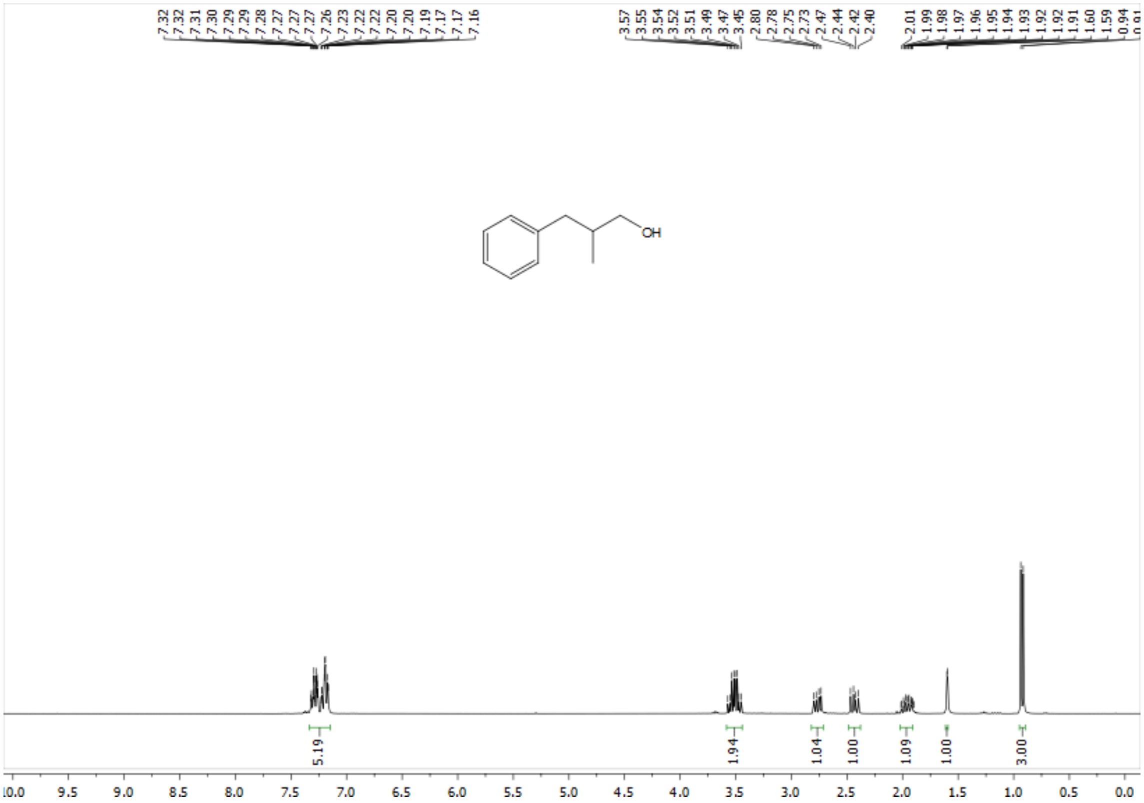

Figure S56: ^{1}H NMR (400 MHz, $CDCl_3$, 298 K) spectrum of 11a

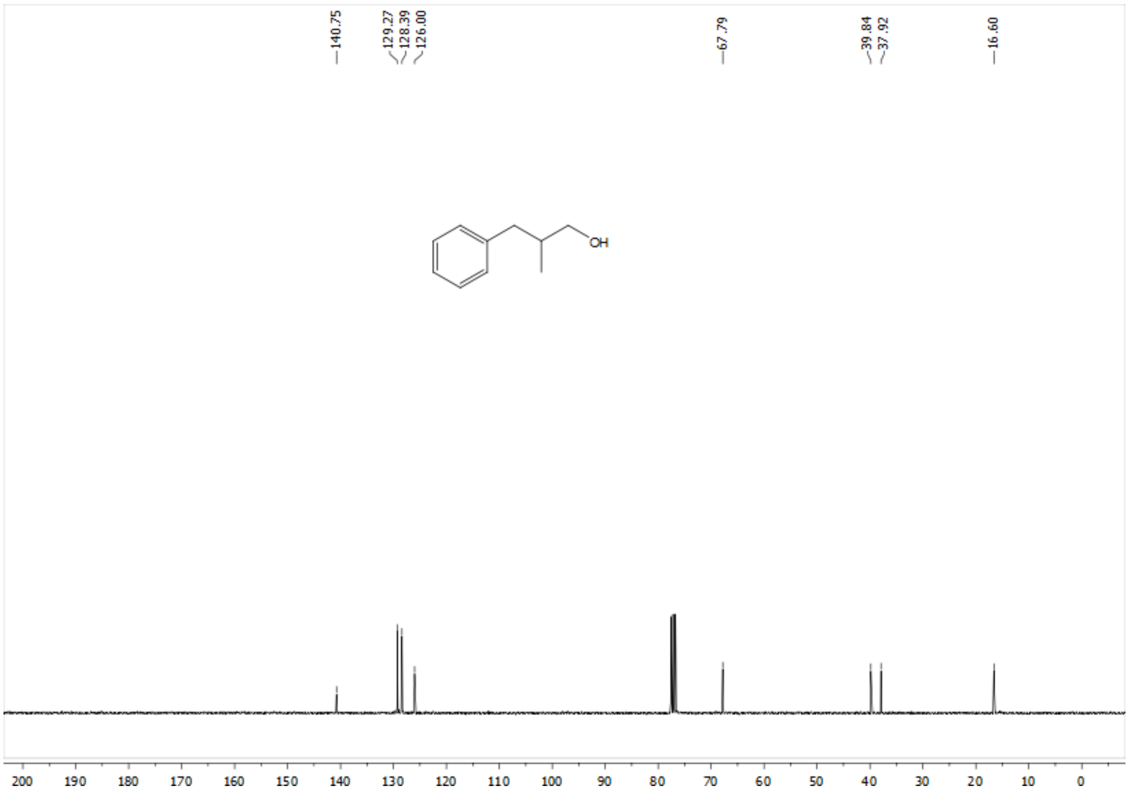

Figure S57: $^{13}C\{^{1}H\}$ NMR (101 MHz, $CDCl_3$, 298 K) spectrum of 11a

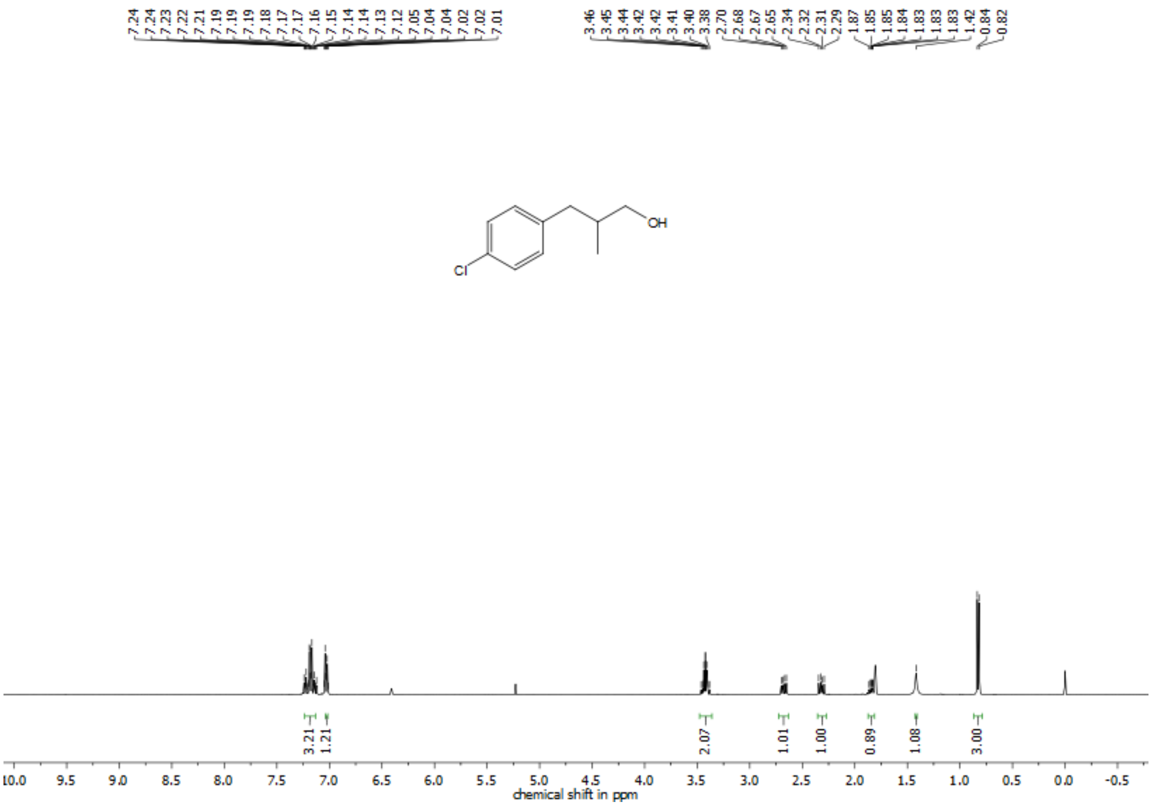

Figure S58: ^{1}H NMR (400 MHz, $CDCl_3$, 298 K) spectrum of 11b

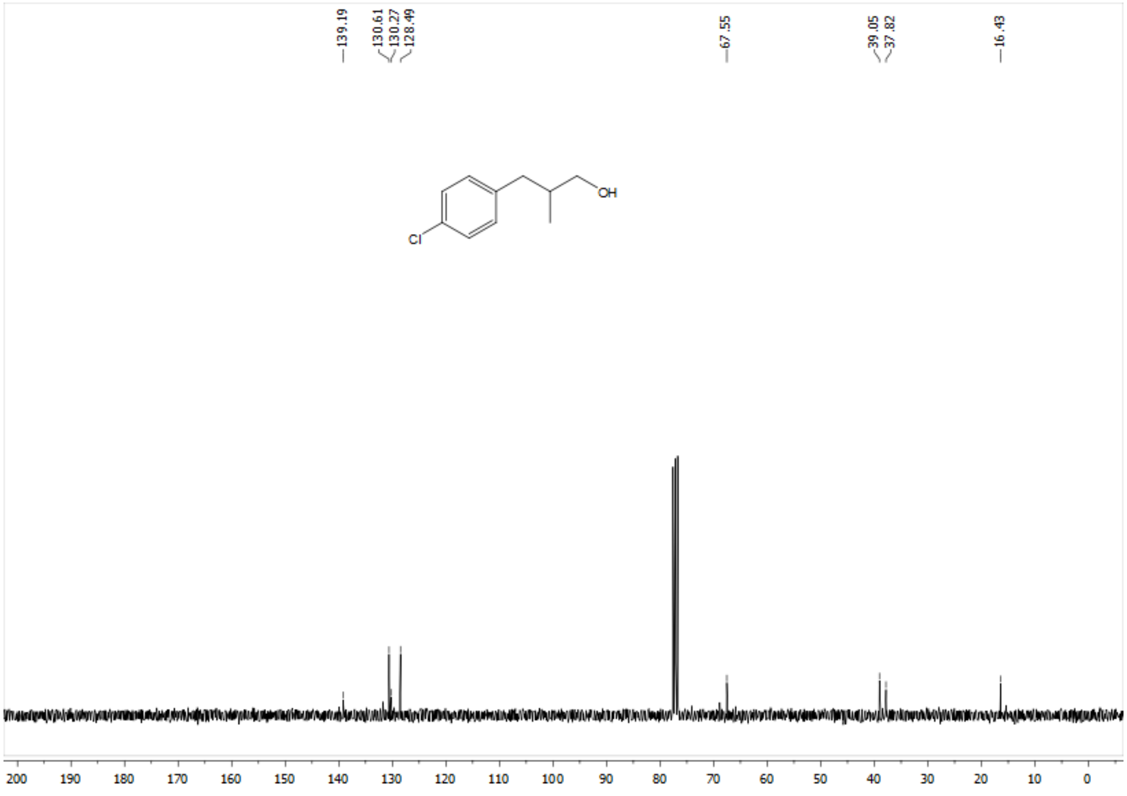

Figure S59: $^{13}C\{^{1}H\}$ NMR (75 MHz, $CDCl_3$, 298 K) spectrum for 11b

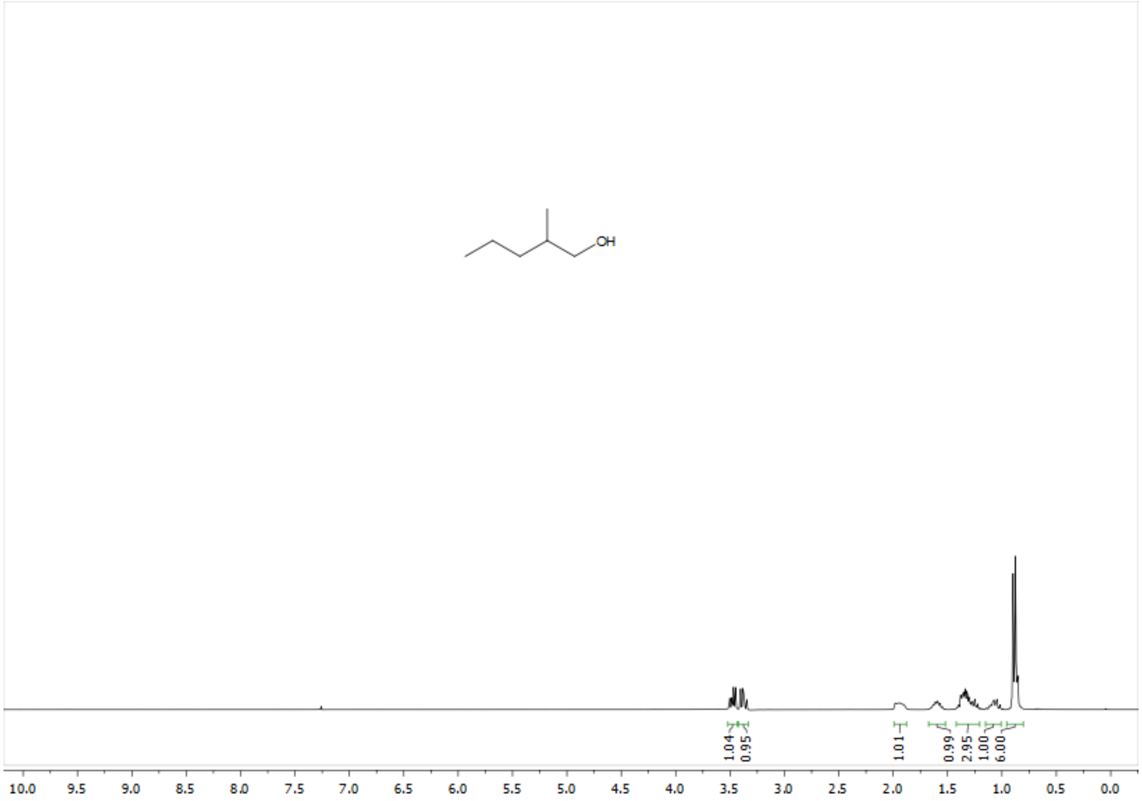

Figure S60: ^{1}H **NMR (300 MHz,** $CDCl_3$**, 298 K) spectrum for 11g**

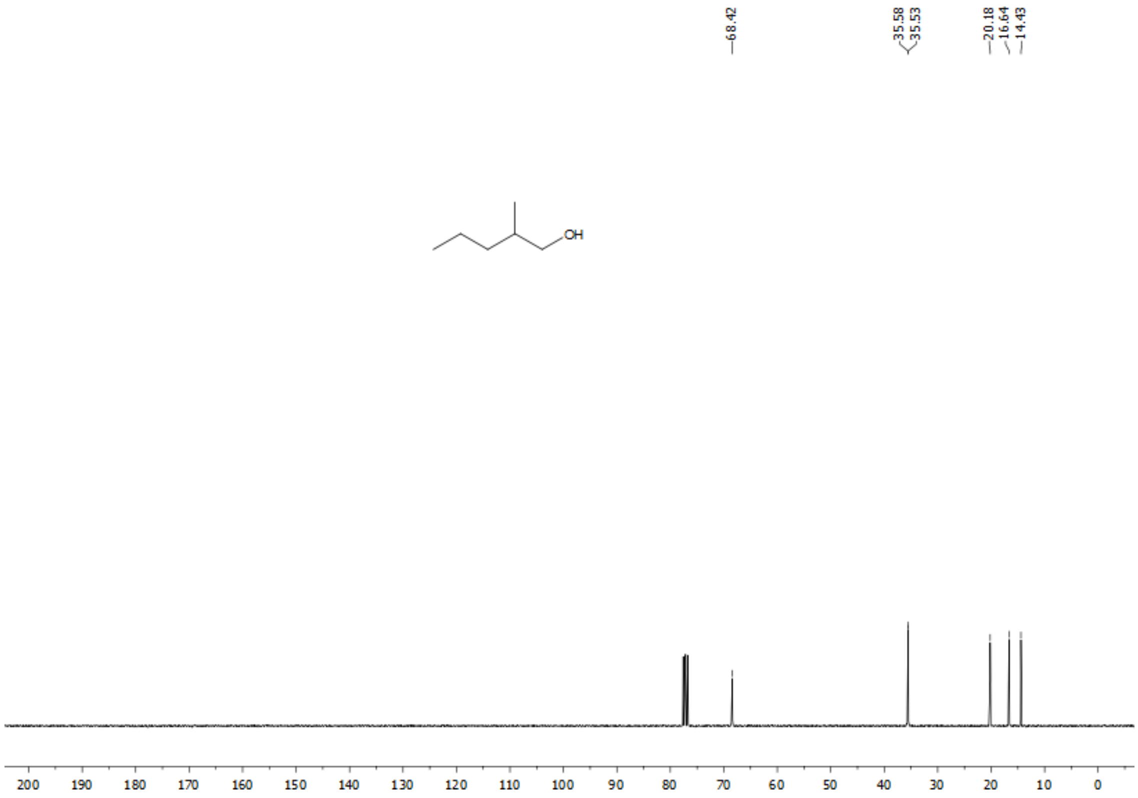

Figure S61: $^{13}C\{^{1}H\}$ **NMR (75 MHz,** $CDCl_3$**, 298 K) spectrum for 11g**

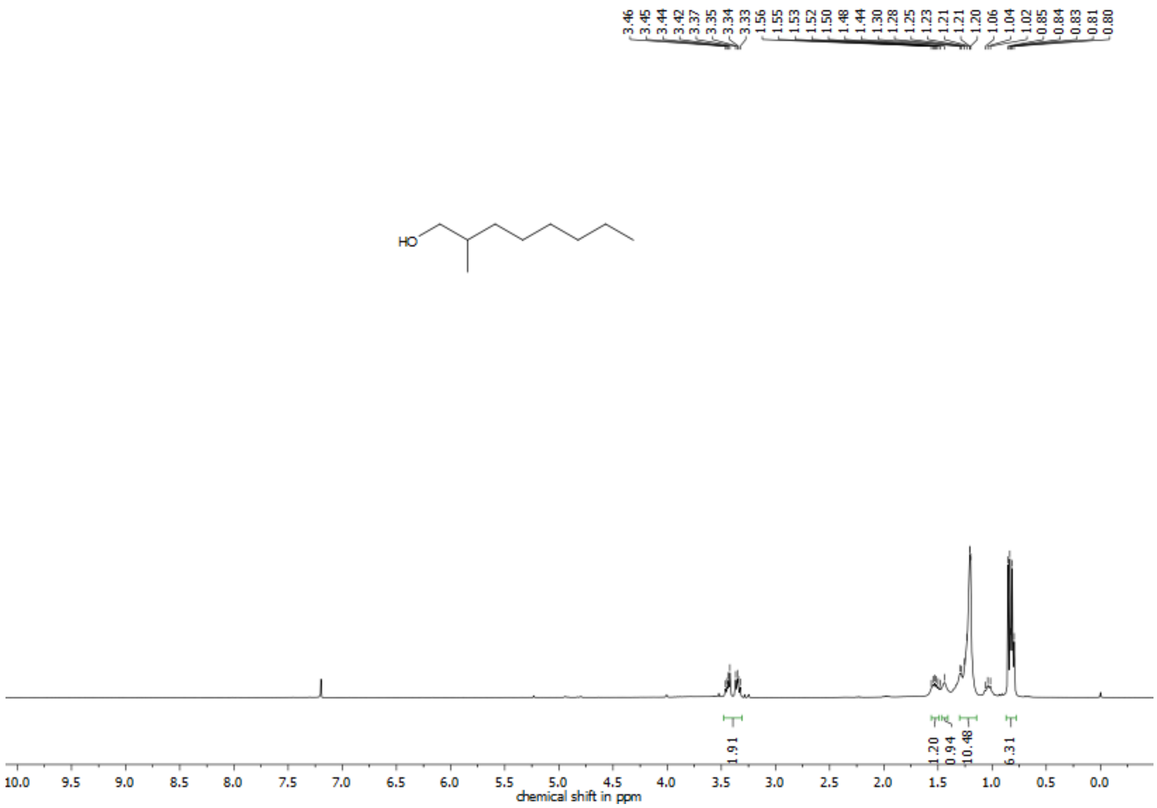

Figure S62: ^{1}H NMR (400 MHz, $CDCl_3$, 298 K) spectrum for 11i

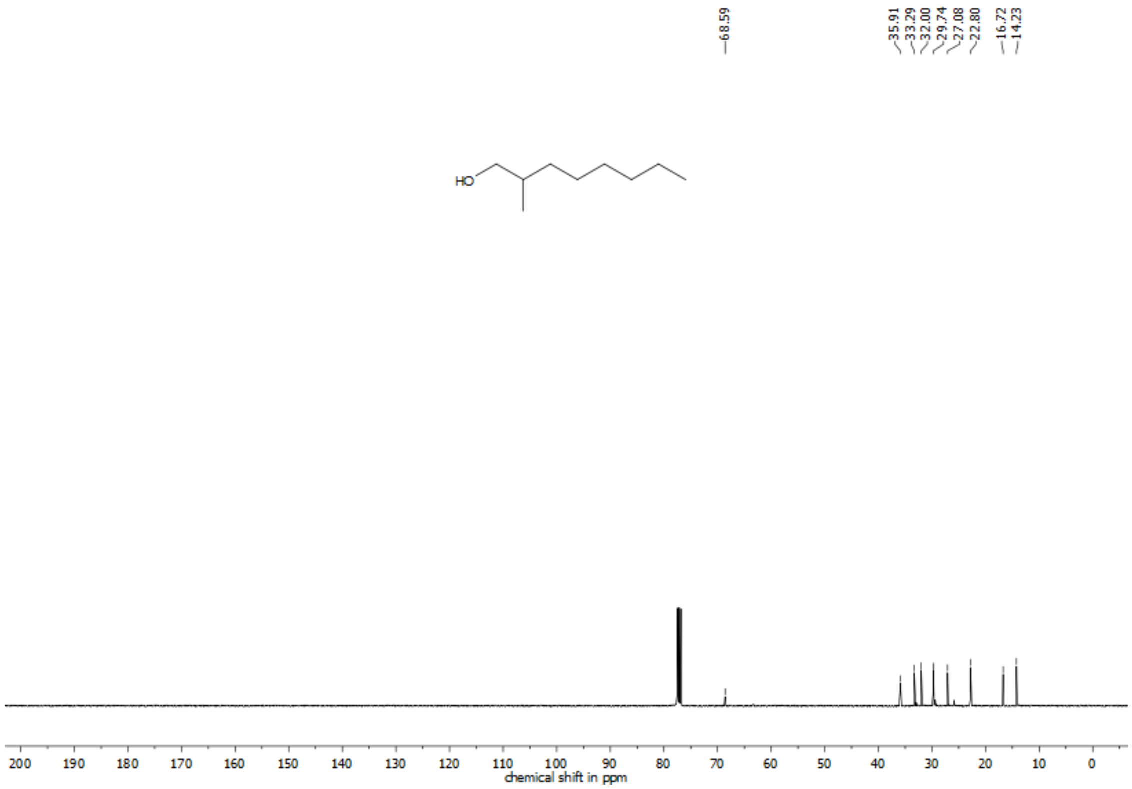

Figure S63: $^{13}C\{^{1}H\}$ NMR (101 MHz, $CDCl_3$, 298 K) spectrum for 11i

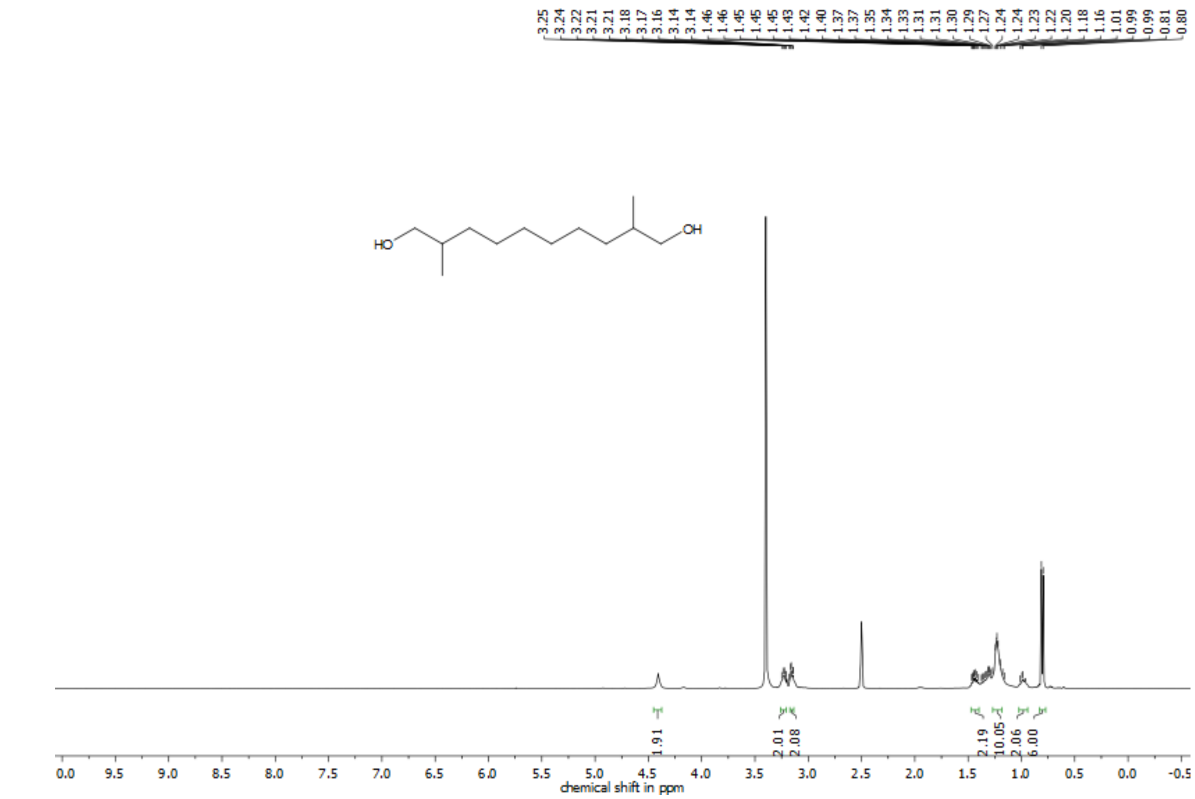

Figure S64: ^{1}H NMR (400 MHz, DMSO-d_6, 298 K) spectrum for 11l

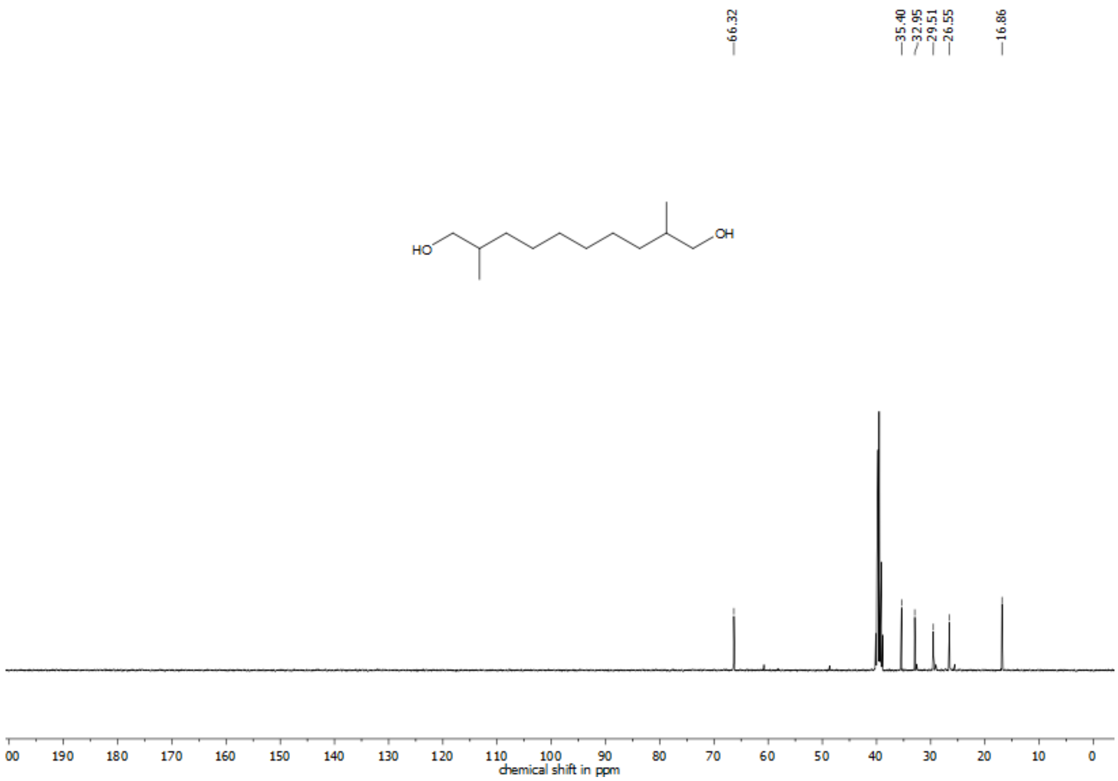

Figure S65: $^{13}C\{^{1}H\}$ NMR (101 MHz, DMSO-d_6, 298 K) spectrum for 11l

10. Conversion/time profile from experiments for the β-methylation of 6a with MeOH at different time intervals

Nine reactions were performed for the reaction progress experiments at different time intervals. Mn-MACHO **1** (2.48 mg, 0.5 mol%) and NaOMe (108.04 mg, 2 mmol) were measured into a glass inlet equipped with a stirring bar inside a glovebox. The glass inlet was closed with a septum and transferred into the bottom part of the 10 mL steel autoclave, where it was opened under a stream of argon. After sealing, the autoclave was purged with argon three times. **6a** (122.1 mg, 1 mmol), and MeOH (1 mL) were added at room temperature through a valve under argon. The autoclave was sealed and heated to 150 ^{o}C temperature. After the desired reaction time, the autoclave was cooled to room temperature and slowly vented while stirring continued. Mesitylene was added as an internal standard to the reaction mixture that was then passed through a short path of acidic alumina before the composition was analyzed by NMR spectroscopy. Yield was determined by ^{1}H NMR spectrum using mesitylene as an internal standard ($\delta_{Mesitylene(standard)}$ = 6.72 (s, 3H), $\delta_{product}$ = 3.58 (d, 2H)). The graph is shown in the manuscript which corresponds to Figure 1.

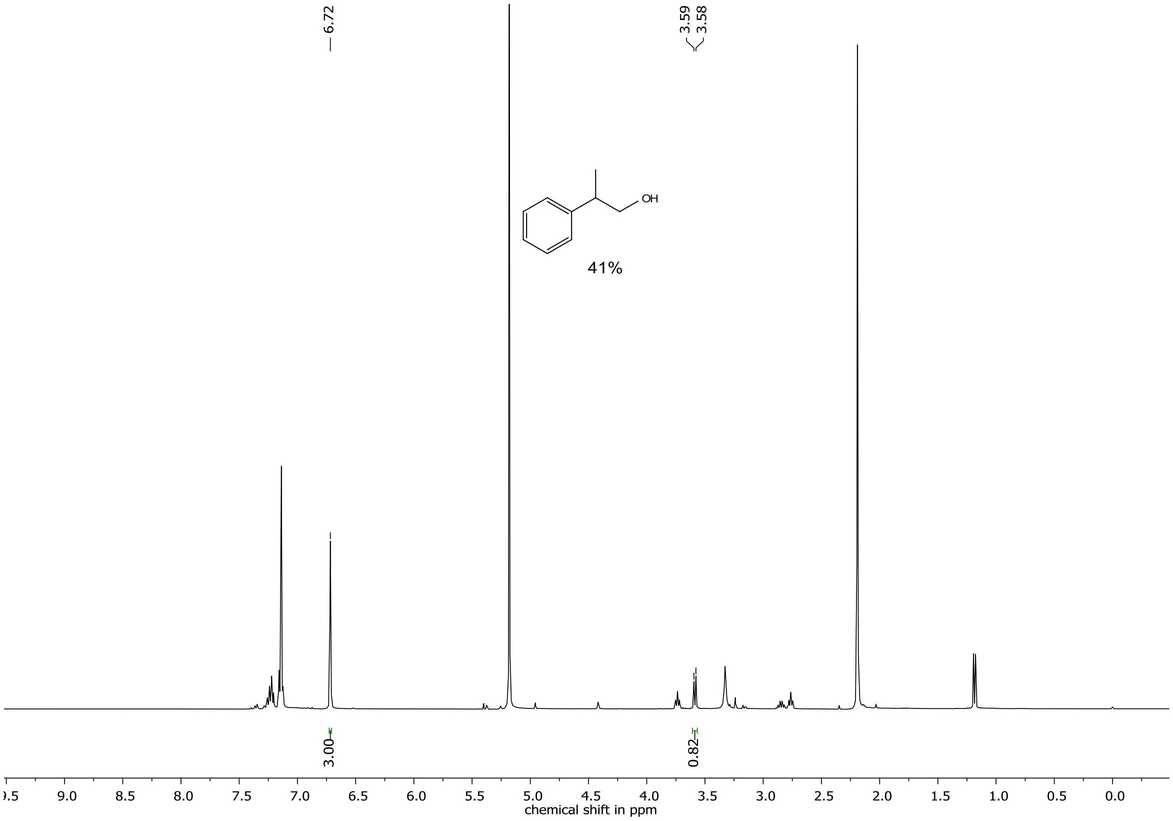

Figure S66: ^{1}H NMR (300 MHz, $CDCl_3$, 298 K) spectrum of 6a with MeOH after 2.00 h as example

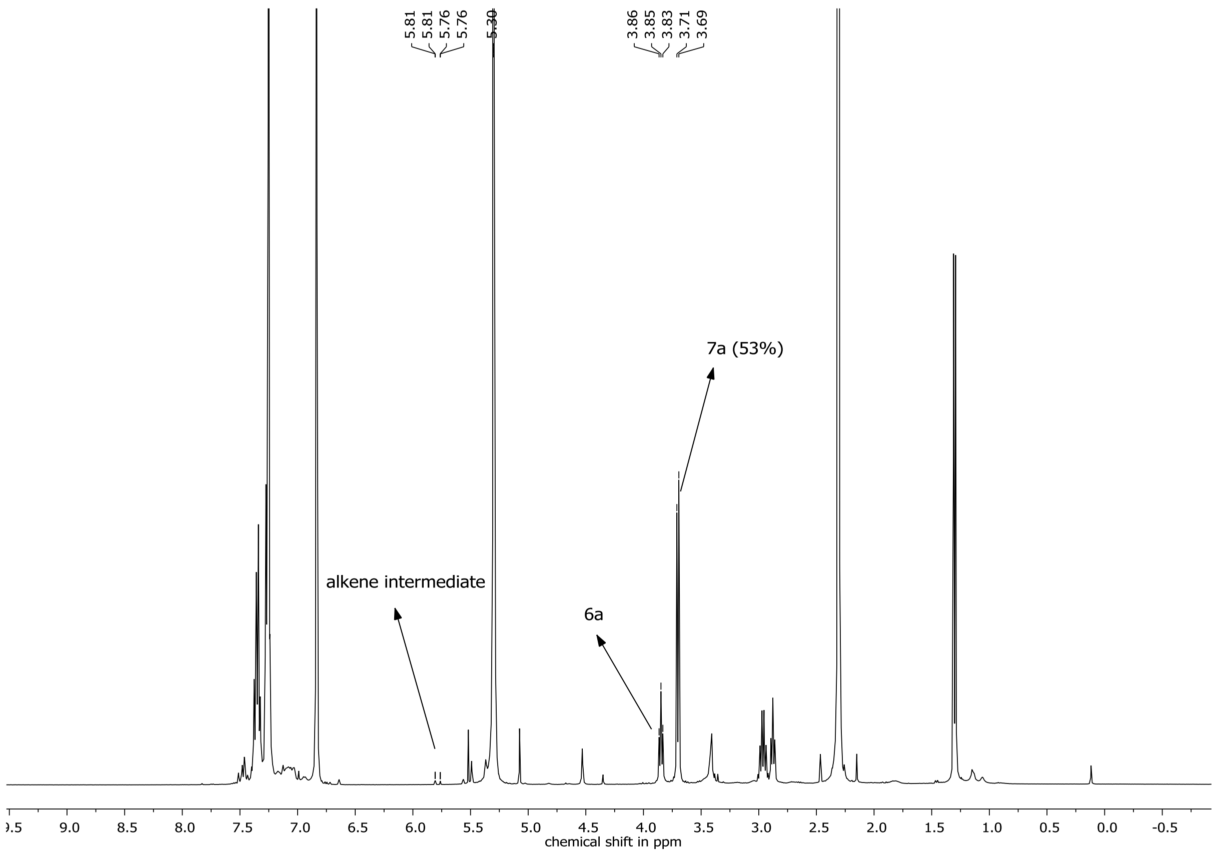

Figure S67: ^{1}H NMR (300 MHz, $CDCl_3$, 298 K) spectrum of 6a with MeOH after 4.00 h as example

11. Labeling experiments and observation of potential intermediates

11.1. Procedure for β-methylation of 6a with $^{13}CH_3OH$

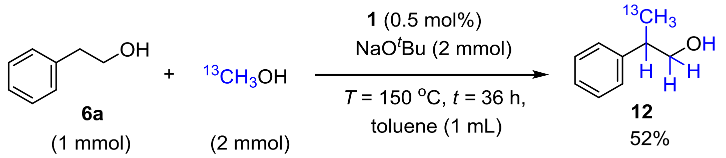

Mn-MACHO **1** (2.48 mg, 0.5 mol%) and NaO^{t}Bu (192.3 mg, 2 mmol) were measured into a glass inlet equipped with a stirring bar inside a glovebox. The glass inlet was closed with a septum and transferred into the bottom part of the 10 mL steel autoclave, where it was opened under a stream of argon. After sealing, the autoclave was purged with argon three times. **6a** (122.1 mg, 1 mmol), $^{13}CH_3OH$ (66.06 mg, 2 mmol) and toluene (1 mL) were added at room temperature through a valve under argon. The autoclave was sealed and heated to 150 °C temperature for 36 h. After completion of the reaction, the autoclave was cooled to room temperature and slowly vented while stirring continued. Mesitylene (120 mg, 1 mmol) was added as an internal standard to the reaction mixture that was then passed through a short path of acidic alumina before the composition was analyzed by NMR spectroscopy. Yield was determined by ^1H NMR spectrum using mesitylene as an internal standard ($\delta_{Mesitylene(standard)}$ = 6.98 (s, 3H), $\delta_{product}$ = 3.80-3.84 (m, 2H)).

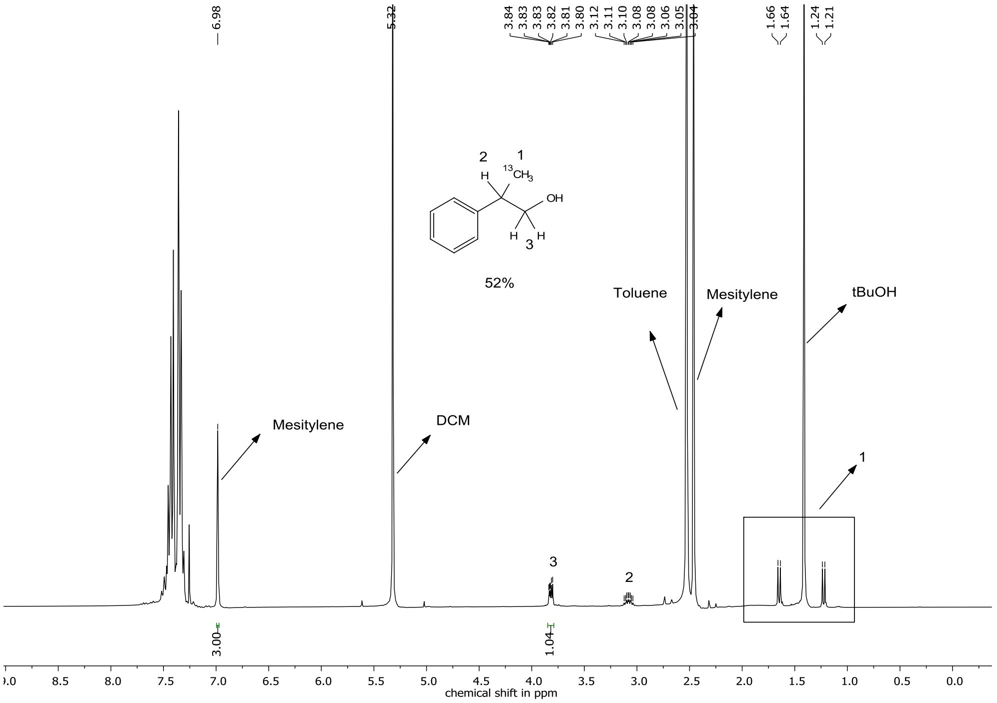

Figure S68: ^1H NMR (300 MHz, $CDCl_3$, 298 K) spectrum for the β-Methylation of 6a with $^{13}CH_3OH$

11.2. Procedure for selective β-methylation of 6a with CD_3OD

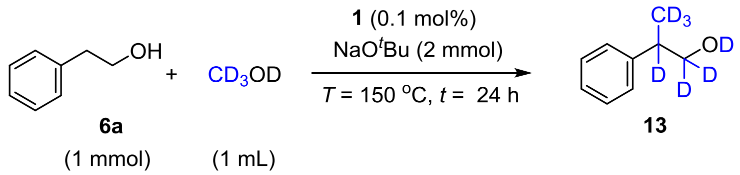

Mn-MACHO **1** (2.48 mg, 0.5 mol%) and NaO^tBu (192.3 mg, 2 mmol) were measured into a glass inlet equipped with a stirring bar inside a glovebox. The glass inlet was closed with a septum and transferred into the bottom part of the 10 mL steel autoclave, where it was opened under a stream of argon. After sealing, the autoclave was purged with argon three times. **6a** (122.1 mg, 1 mmol) and CD_3OD (1 mL) were added at room temperature through a valve under argon. The autoclave was sealed and heated to 150 °C temperature for 24 h. After 24 h, the autoclave was cooled to room temperature and slowly vented while stirring continued. Mesitylene (120 mg, 1 mmol) was added as an internal standard to the reaction mixture that was then passed through a short path of acidic alumina before the composition was analyzed by NMR spectroscopy. **$^{13}C\{^1H\}$-NMR** (101 MHz, $CDCl_3$) δ 143.72 (quat-*C*), 128.64 (Ar*C*H), 127.51 (Ar*C*H), 126.66 (quat-*C*), 67.80-68.48 (m, *C*D_2), 41.42-42.12 (m, *C*D), 16.47-16.93 (m, *C*D_3).

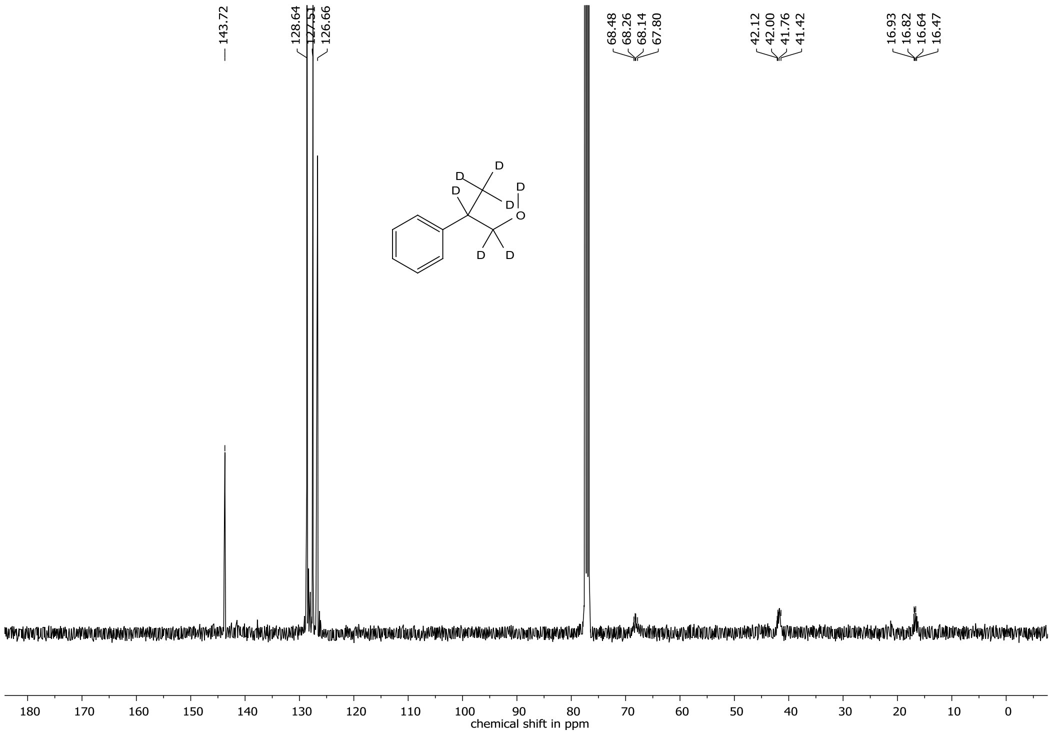

Figure S69: $^{13}C\{^1H\}$ NMR (75 MHz, $CDCl_3$, 298 K) spectrum for the β-Methylation of 6a with CD_3OD

11.3. Procedure for β-methylation of 6a with paraformaldehyde and H_2

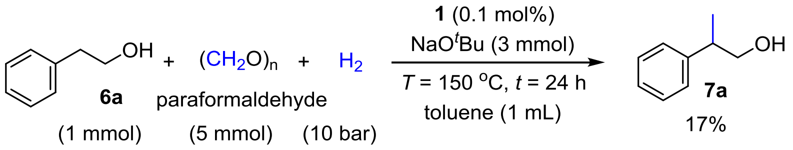

Mn-MACHO **1** (2.48 mg, 0.5 mol%), NaO^{t}Bu (192.3 mg, 2 mmol) and paraformaldehyde (150.1 mg, 5 mmol) were measured into a glass inlet equipped with a stirring bar inside a glovebox. The glass inlet was transferred to a 10 mL stainless autoclave that was evacuated and refilled with argon at least three times. **6a** (122.1 mg, 1 mmol) and toluene (1 mL) were added at room temperature through a valve under argon. The autoclave was sealed and pressurized with 10 bar of hydrogen. The autoclave was heated to 150 °C temperature for 24 h. After 24 h, the autoclave was cooled to room temperature and slowly vented while stirring continued. Mesitylene (120 mg, 1 mmol) was added as an internal standard to the reaction mixture that was then passed through a short path of acidic alumina before the composition was analyzed by NMR spectroscopy. Yield was determined by ^{1}H NMR spectrum using mesitylene as an internal standard ($\delta_{Mesitylene(standard)}$=6.84 (s, 3H), $\delta_{product}$= 3.71 (d, 2H)).

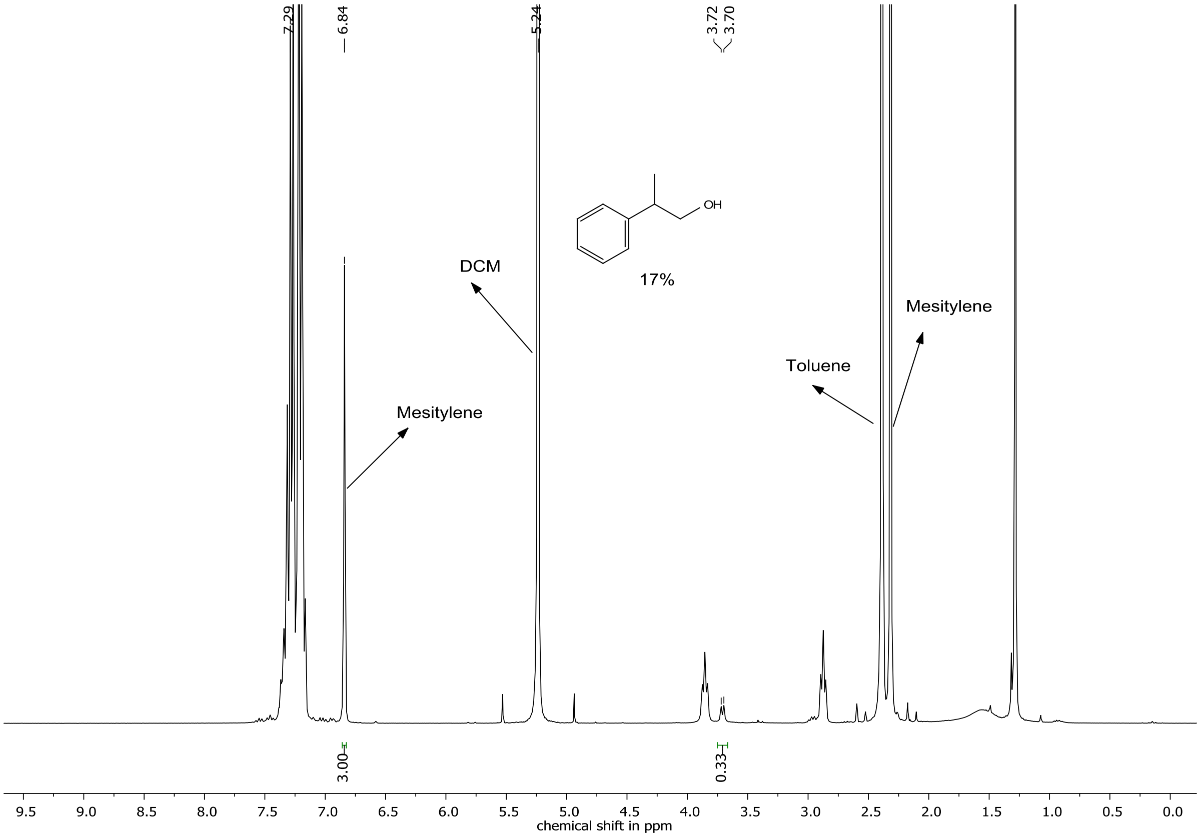

Figure S70: ^{1}H NMR (300 MHz, $CDCl_3$, 298 K) spectrum for the β-Methylation of 6a with paraformaldehyde and H_2

11.4. Procedure for β-methylation of Acetophenone (14) with methanol

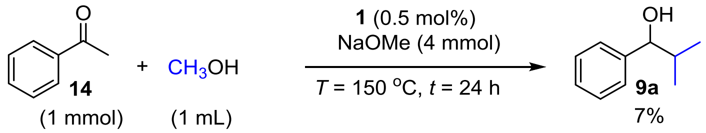

Mn-MACHO **1** (2.48 mg, 0.5 mol%) and NaOMe (216.08 mg, 4 mmol) were measured into a glass inlet equipped with a stirring bar inside a glovebox. The glass inlet was closed with a septum and transferred into the bottom part of the 10 mL steel autoclave, where it was opened under a stream of argon. After sealing, the autoclave was purged with argon three times. Acetophenone **14** (120.2 mg, 1 mmol) and methanol (1 mL) were added at room temperature through a valve under argon. The autoclave was sealed and heated to 150 °C temperature. After 24 h, the autoclave was cooled to room temperature and slowly vented while stirring continued. Mesitylene was added as an internal standard to the reaction mixture that was then passed through a short path of acidic alumina before the composition was analyzed by NMR spectroscopy.

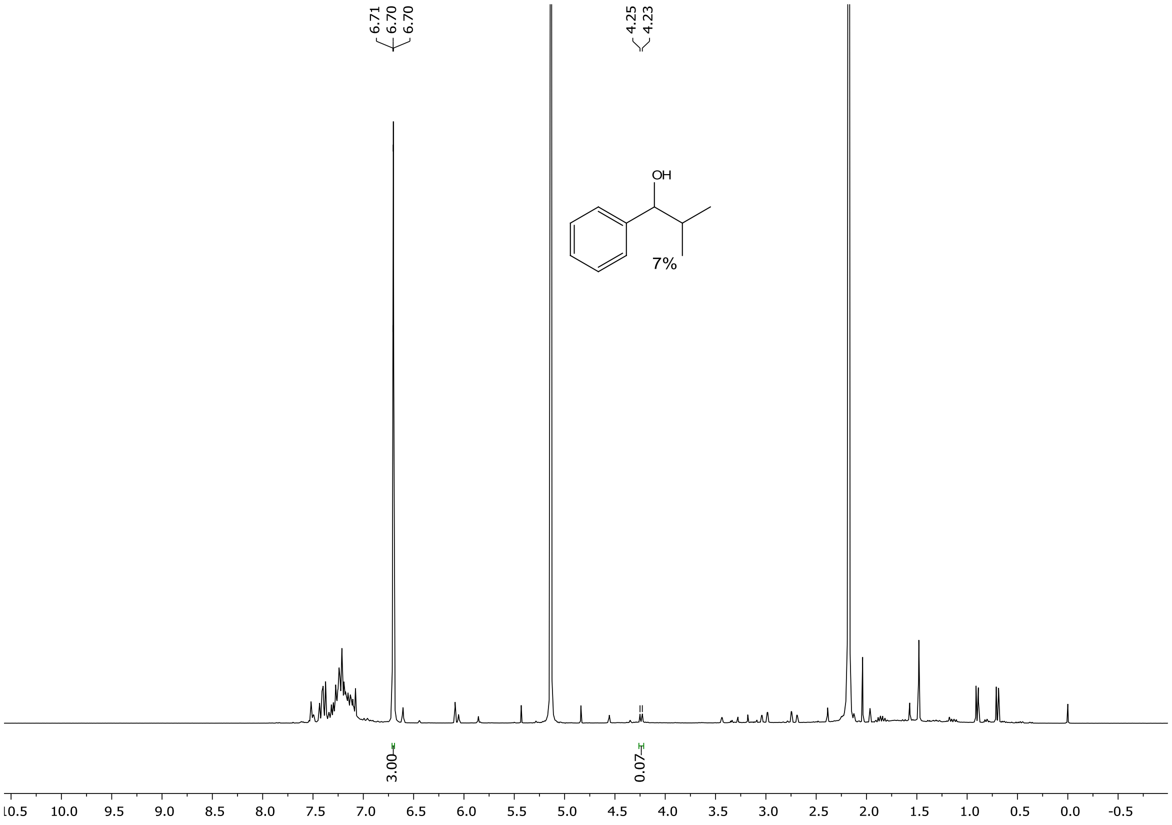

Figure S71: ^{1}H NMR (300 MHz, $CDCl_3$, 298 K) spectrum for the β-Methylation of Acetophenone 14 with methanol

11.5. Preparation of complex 15

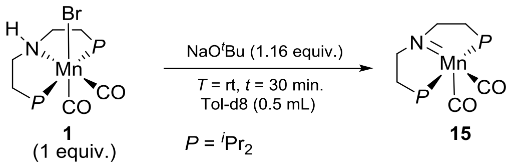

A high pressure NMR tube was charged with complex **1** (15 mg, 0.03 mmol), NaO^{t}Bu (3.36 mg, 0.035 mmol) and Tol.-d8 (0.4 mL). The resulting solution was stirred for 30 minutes at room temperature leading to a color change from yellow to orange red. ^{1}H-NMR (300 MHz, Tol-d8, 298 K) and ^{31}P{^{1}H}-NMR (121 MHz, Tol-d8, 298 K) spectroscopies are consistent with the formation of complex **15**. [1]

11.6. Reaction of Mn-complex (1) with 2-phenyl ethanol (6a)

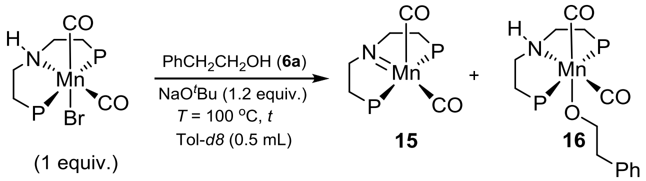

6a (1.22 mg, 0.01 mmol), Mn-complex **1** (4.95 mg, 0.01 mmol), NaO^{t}Bu (1.2 mg, 0.012 mmol) and Tol-d8 (0.5 ml as a solvent) were charged in a Teflon tapped NMR tube under argon atmosphere. The NMR tube was heated at 100 °C temperature.^{31}P{^{1}H} NMR of the reaction mixture of **1** and **6a** were measured at different time intervals which confirmed the formation of Mn(I) intermediate **15** and **16**.[1]

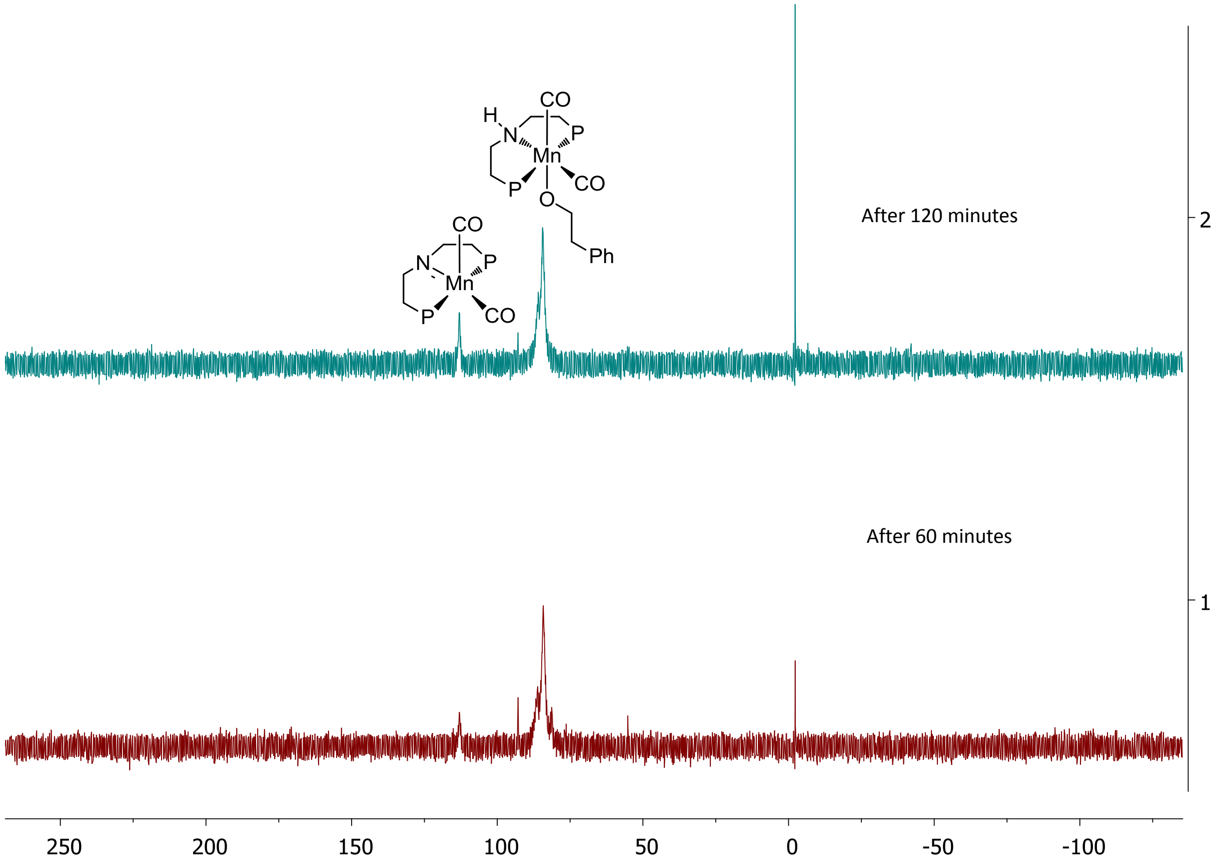

Figure S72: Superposed ^{31}P{^{1}H}-NMR spectrum of Mn-intermediates 15 and 16 at different time intervals

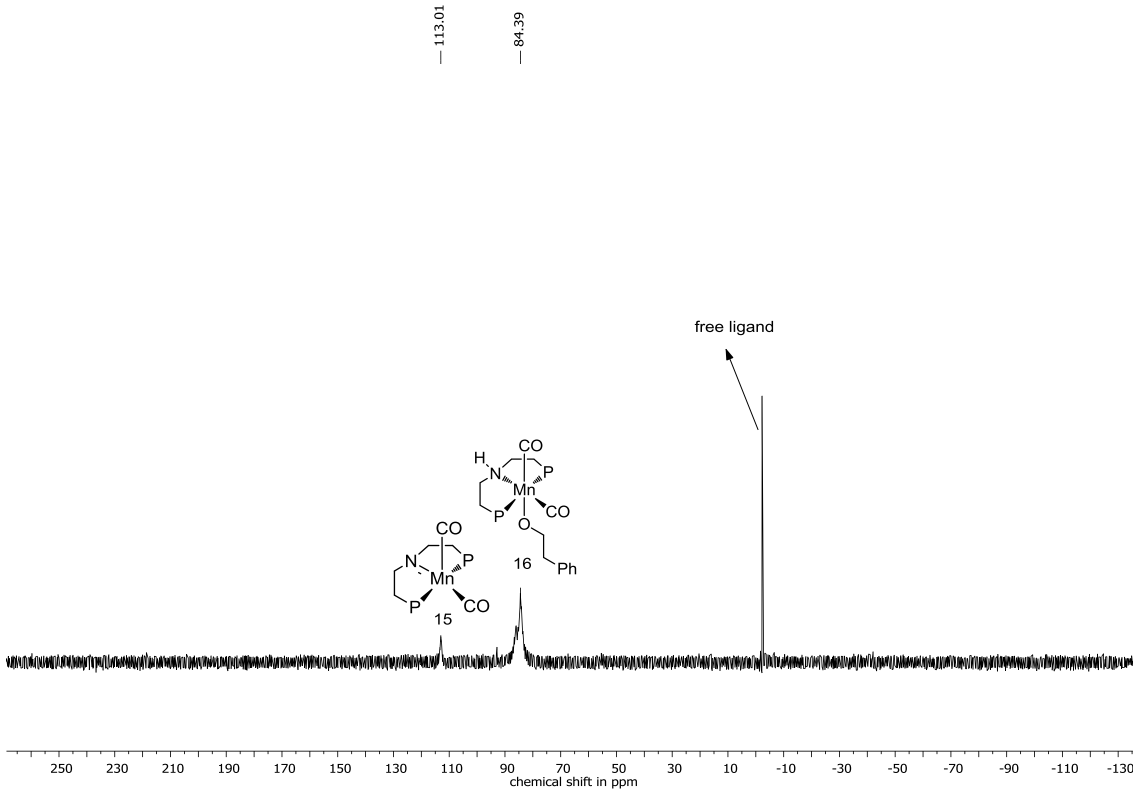

Figure S73: $^{31}P\{^1H\}$-NMR (121 MHz, Toluene-d_8, 298 K) spectrum of Mn-intermediates 15 and 16

11.7. Reaction of Mn-complex (15) with methanol

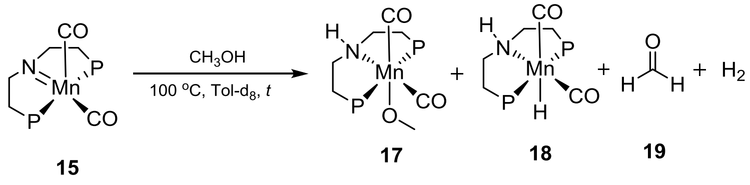

Mn-complex **15** (4.15 mg, 0.01 mmol), MeOH (1.6 mg, 0.05 mmol) and Tol-d8 (0.4 ml as a solvent) were charged in a teflon tapped NMR tube under argon atmosphere. The NMR tube was heated at 100 °C temperature. ^1H NMR of the reaction mixture of **15** and methanol were measured at different time intervals which confirmed the formation of formaldehyde, H_2 and Mn(I) intermediates **17** and **18** respectively.[1]

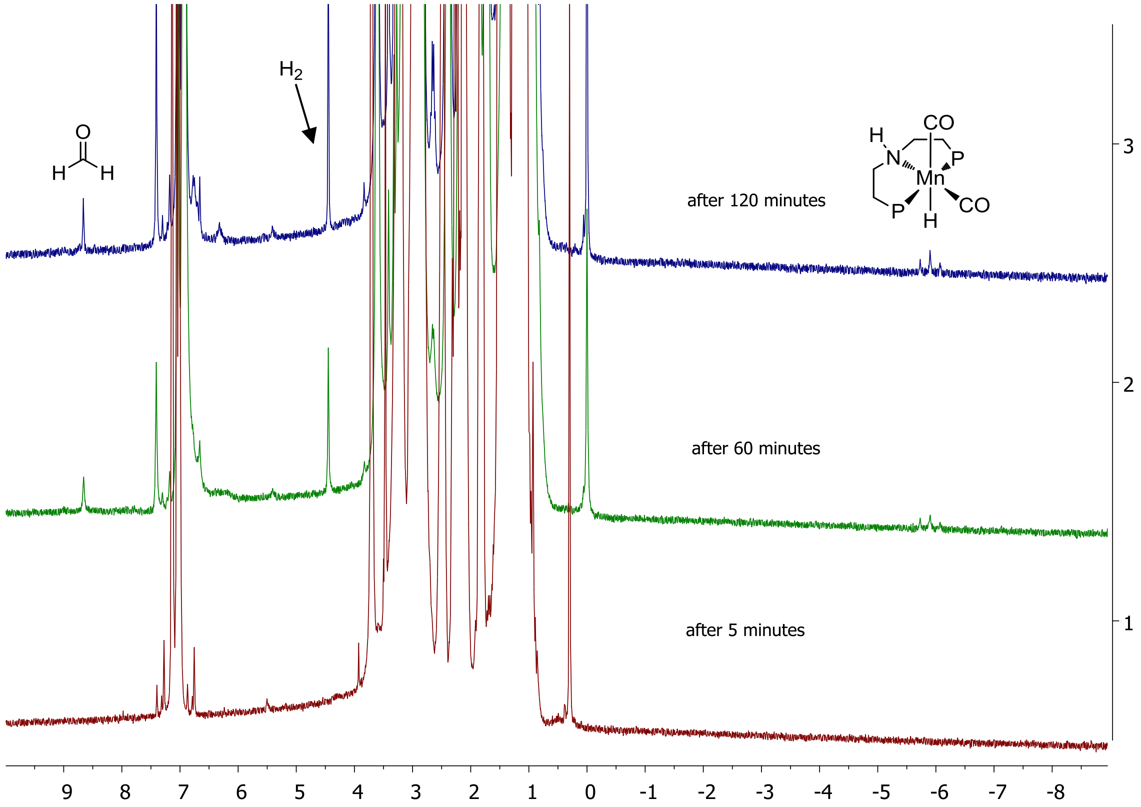

Figure S74: Superposed ^1H-NMR spectra of formaldehyde, H_2 and intermediate 18 at different time intervals

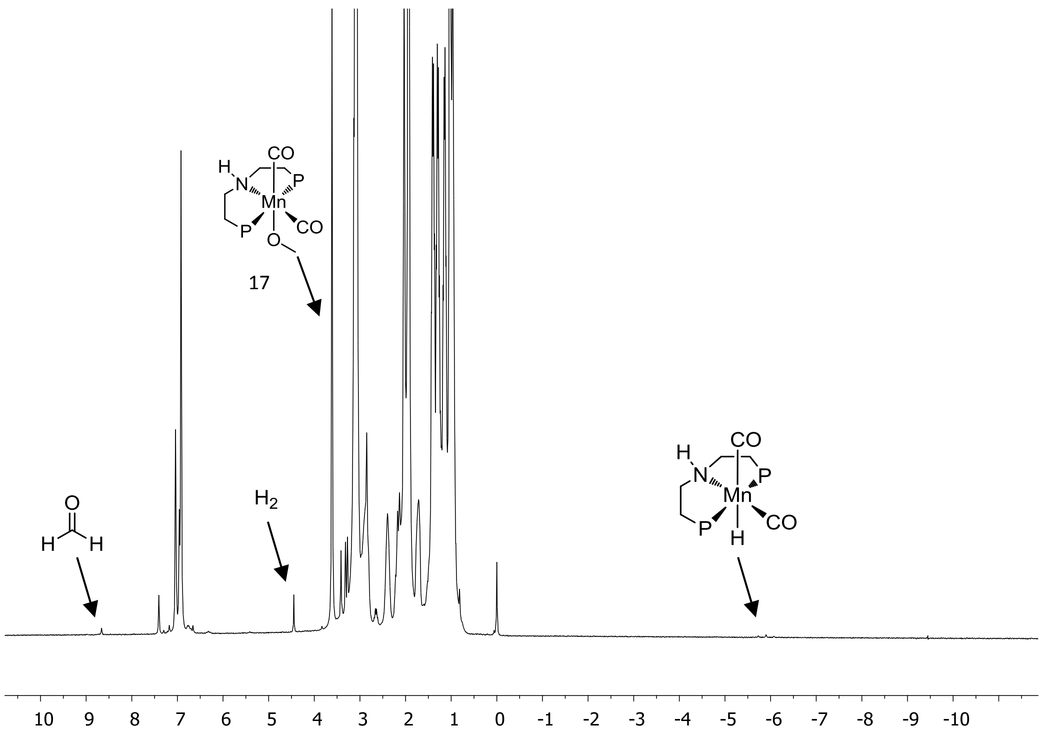

Figure S75: ^{1}H-NMR (300 MHz, tol-*d8*, 298 K) spectrum of intermediates 17 and 18 after 120 minutes at 100 °C

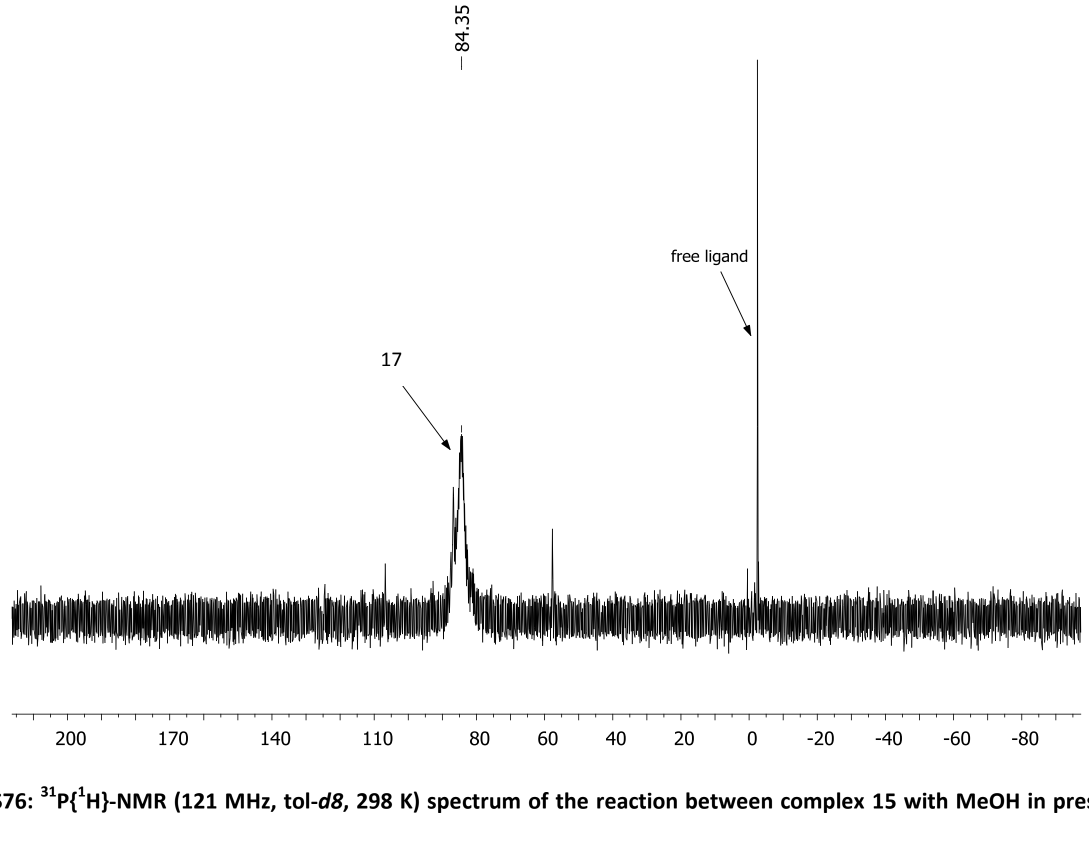

Figure S76: $^{31}P\{^{1}H\}$-NMR (121 MHz, tol-*d8*, 298 K) spectrum of the reaction between complex 15 with MeOH in presence of base

References

[1] A. Kaithal, M. Hölscher, W. Leitner, *Angew. Chem. Int. Ed.* **2018**, *57*, 13449-13453.

[2] A. Kaithal, S. Sen, C. Erken, T. Weyhermüller, M. Hölscher, C. Werlé, W. Leitner, *Nat. Commun.* **2018**, *9*, 4521.

[3] K. Oikawa, S. Itoh, H. Yano, H. Kawasaki, Y. Obora, *Chem. Commun.* **2017**, *53*, 1080-1083.

[4] J. C. Bardhan, D. N. Mukherji, *J. Chem. Soc.* **1956**, 4629-4633.

[5] S. Koul, J. L. Koul, B. Singh, M. Kapoor, R. Parshad, K. S. Manhas, S. C. Taneja, G. N. Qazi, *Tetrahedron: Asymmetry* **2005**, *16*, 2575-2591.

[6] A. Gansäuer, M. Klatte, G. M. Brändle, J. Friedrich, *Angew. Chem. Int. Ed.* **2012**, *51*, 8891-8894.

[7] Y. Li, H. Li, H. Junge, M. Beller, *Chem. Commun.* **2014**, *50*, 14991-14994.

[8] K. Polidano, J. M. J. Williams, L. C. Morrill, *ACS Catal.* **2019**, *9*, 8575-8580.

[9] A. Kaithal, M. Schmitz, M. Hölscher, W. Leitner, *ChemCatChem* **2019**, DOI: 10.1002/cctc.201900788.

[10] T. Slagbrand, T. Kivijärvi, H. Adolfsson, *ChemCatChem* **2015**, *7*, 3445-3449.

[11] G. V. M. Sharma, K. L. Reddy, P. S. Lakshmi, R. Ravi, A. C. Kunwar, *J. Org. Chem.* **2006**, *71*, 3967-3969.

[12] K. Polidano, B. G. Reed-Berendt, A. Basset, A. J. A. Watson, J. M. J. Williams, L. C. Morrill, *Org. Lett.* **2017**, *19*, 6716-6719.

[13] G. Zhao, R. Tong, *Green Chemistry* **2019**, *21*, 64-68.

[14] M. Garbe, Z. Wei, B. Tannert, A. Spannenberg, H. Jiao, S. Bachmann, M. Scalone, K. Junge, M. Beller, *Adv. Synth. Catal.* **2019**, *361*, 1913-1920.

[15] a) D. S. Rao, T. R. Reddy, K. Babachary, S. Kashyap, *Org. Biomol. Chem* **2016**, *14*, 7529-7543; b) R. Fernández, A. Ros, A. Magriz, H. Dietrich, J. M. Lassaletta, *Tetrahedron* **2007**, *63*, 6755-6763.

[16] a) A. Kišić, M. Stephan, B. Mohar, *Adv. Synth. Catal.* **2015**, *357*, 2540-2546; b) S. Rodríguez, B. Qu, K. R. Fandrick, F. Buono, N. Haddad, Y. Xu, M. A. Herbage, X. Zeng, S. Ma, N. Grinberg, H. Lee, Z. S. Han, N. K. Yee, C. H. Senanayake, *Adv. Synth. Catal.* **2014**, *356*, 301-307.

[17] W. J. Jang, S. M. Song, J. H. Moon, J. Y. Lee, J. Yun, *J. Am. Chem. Soc.* **2017**, *139*, 13660-13663.

[18] Q. Wang, X. Liu, X. Liu, B. Li, H. Nie, S. Zhang, W. Chen, *Chem. Commun.* **2014**, *50*, 978-980.

[19] J. E. Roque Pena, E. J. Alexanian, *Org. Lett.* **2017**, *19*, 4413-4415.

[20] J. Cao, P. Perlmutter, *Org. Lett.* **2013**, *15*, 4327-4329.

[21] G. R. Dowson, M. F. Haddow, J. Lee, R. L. Wingad, D. F. Wass, *Angew. Chem. Int. Ed.* **2013**, *52*, 9005-9008.

[22] P. H. Galebach, D. J. McClelland, N. M. Eagan, A. M. Wittrig, J. S. Buchanan, J. A. Dumesic, G. W. Huber, *ACS Sustain. Chem. Eng.* **2018**, *6*, 4330-4344.

[23] S. R. Tamang, M. Findlater, *J Org Chem* **2017**.

[24] I. Burkhardt, J. S. Dickschat, *Eur. J. Org. Chem.* **2018**, *2018*, 3144-3157.

[25] P. Wang, D.-L. Wang, H. Liu, X.-L. Zhao, Y. Lu, Y. Liu, *Organometallics* **2017**, *36*, 2404-2411.

[26] B. Cao, X. Chen, Y. Yamaryo-Botte, M. B. Richardson, K. L. Martin, G. N. Khairallah, T. W. T. Rupasinghe, R. M. O'Flaherty, R. A. J. O'Hair, J. E. Ralton, P. K. Crellin, R. L. Coppel, M. J. McConville, S. J. Williams, *J. Org. Chem.* **2013**, *78*, 2175-2190.

[27] H. Ohta, H. Tetsukawa, N. Noto, *J. Org. Chem.* **1982**, *47*, 2400-2404.

[28] K. Shin, S. Joung, Y. Kim, S. Chang, *Adv. Synth. Catal.* **2017**, *359*, 3428-3436.
